# Efficiency of different strategies to mitigate ascertainment bias when using SNP panels in diversity studies

**DOI:** 10.1186/s12864-017-4416-9

**Published:** 2018-01-05

**Authors:** Dorcus Kholofelo Malomane, Christian Reimer, Steffen Weigend, Annett Weigend, Ahmad Reza Sharifi, Henner Simianer

**Affiliations:** 1 Animal Breeding and Genetics Group, Department of Animal Sciences, University of Goettingen, Albrecht-Thaer-Weg 3, 37075 Goettingen, Germany; 2 Institute of Farm Animal Genetics, Friedrich-Loeffler-Institut, Höltystraße 10, 31535 Neustadt, Germany

**Keywords:** SNP filtering, Ascertainment bias, LD based pruning, SNP panels

## Abstract

**Background:**

Single nucleotide polymorphism (SNP) panels have been widely used to study genomic variations within and between populations. Methods of SNP discovery have been a matter of debate for their potential of introducing ascertainment bias, and genetic diversity results obtained from the SNP genotype data can be misleading. We used a total of 42 chicken populations where both individual genotyped array data and pool whole genome resequencing (WGS) data were available. We compared allele frequency distributions and genetic diversity measures (expected heterozygosity (*H*_*e*_), fixation index (*F*_*ST*_) values, genetic distances and principal components analysis (PCA)) between the two data types. With the array data, we applied different filtering options (SNPs polymorphic in samples of two *Gallus gallus* wild populations, linkage disequilibrium (LD) based pruning and minor allele frequency (MAF) filtering, and combinations thereof) to assess their potential to mitigate the ascertainment bias.

**Results:**

Rare SNPs were underrepresented in the array data. Array data consistently overestimated *H*_*e*_ compared to WGS data, however, with a similar ranking of the breeds, as demonstrated by Spearman’s rank correlations ranging between 0.956 and 0.985. LD based pruning resulted in a reduced overestimation of *H*_*e*_ compared to the other filters and slightly improved the relationship with the WGS results. The raw array data and those with polymorphic SNPs in the wild samples underestimated pairwise *F*_*ST*_ values between breeds which had low *F*_*ST*_ (<0.15) in the WGS, and overestimated this parameter for high WGS *F*_*ST*_ (>0.15). LD based pruned data underestimated *F*_*ST*_ in a consistent manner. The genetic distance matrix from LD pruned data was more closely related to that of WGS than the other array versions. PCA was rather robust in all array versions, since the population structure on the PCA plot was generally well captured in comparison to the WGS data.

**Conclusions:**

Among the tested filtering strategies, LD based pruning was found to account for the effects of ascertainment bias in the relatively best way, producing results which are most comparable to those obtained from WGS data and therefore is recommended for practical use.

**Electronic supplementary material:**

The online version of this article (doi: 10.1186/s12864-017-4416-9) contains supplementary material, which is available to authorized users.

## Background

Following the process of animal domestication, evolutionary forces such as selection and genetic drift have played a critical role in animal diversification. Such forces led to genomic alterations such as fixation of favorable alleles within a breed or species and differentiation from the ancestral state due to successful selection programs or adaptation. This concept of domestication and its subsequent impact on diversity of animal species, breeds or strains was well explored by Darwin [1, 2]. So, phylogenetic studies aim to assess these variations.

The wild, unselected native and village chicken populations retain a reservoir of and exhibit more genetic variability [3–5]. Commercial breeds are known for being intensely selected for economic purposes, i.e. meat and egg type production. Successful egg type selection programs within the commercial layers have resulted in a reduced genetic variability within these lines. In Europe, an organized and systematic breeding in chickens was developed during the nineteenth century. Selection programs in this case were based on producing attractive features (for entertainment) in line with the breed standards; because of this, many fancy breeds were heavily selected for their attractiveness. To date such heavily selected breeds exhibit reduced genetic diversity and high average genetic distances to other breeds [3–5]. Major components for the reduced variability within both the commercial and the fancy breeds are due to the fact that the selection was certainly based on small number of founders, small effective population size and/or high degree of inbreeding.

Using whole genome resequencing (WGS) data is considered as the best way of doing association or diversity studies [6, 7]. It provides a high resolution of the genome information capturing most (and even the finer) details underlying genomic variations. However the cost of whole genome sequencing still is high for application in larger sample sets. Additionally, limitations such as infrastructure (e.g. WGS requires good reference genomes), work effort and time poses further constraints. So, generating WGS data for the required sample size in such studies is challenging [6].

Genotyping tools have been developed to overcome these constraints and have made genotype data available in sufficient numbers. Single nucleotide polymorphism (SNP) panels have been widely used in studies of genomic variation within species [8, 9]. For the construction of such SNP sets, a limited number of individuals selected from populations of interest (the so-called ascertainment group) are used as discovery panels. These individuals are sequenced and provide the basis to select polymorphic loci targeted for further genotyping in a larger set of individuals [9, 10]. SNPs are often selected based on quality, with predefined spacing (e.g. equally spaced) and desired frequency distribution [10], among other criteria.

These methods of SNP discovery may introduce ascertainment bias, hindering classical population genetic methods to provide correct results when applied with SNP genotype data [11, 12]. Ascertainment bias is a systematic deviation of population genetic statistics from a theoretical ‘true’ value, which arises from a non-random selection of set of individuals or biased marker discovery protocols [6, 13].

If the level of ascertainment bias is high, results of population genetic studies could be widely misinterpreted [14]. Thus, exploring the potential systematic effects that the ascertained genotype data can have on the results of diversity studies and finding a way to minimize these effects is crucial.

Differences in the allele frequency distribution between SNP genotype data and WGS data have been commonly used to assess ascertainment bias [6, 11, 15]. An easily verifiable indicator of a potential ascertainment bias is a complete absence of SNPs or an underrepresentation of rare SNPs. Discovery of SNPs is driven by the allele frequency, and with an often small size of the discovery panel, discovering rare SNPs is mostly limited [14]. With the missing rare SNPs, the SNP data may not be an adequate representation of the WGS data. Gorlov et al. [16] argue that missing rare SNPs can lead to loss of valuable information and lessen the ability to detect those rare SNPs in association studies, which may be critical e.g. in the context of rare causal SNPs for rare diseases.

Effects of ascertainment bias on genetic diversity analysis within and between populations have been reported in several studies [9, 13, 17]. One of the assertions is that selection of subpopulations for discovery panels tends to over-represent variability of that ascertainment group. Consequently, effects of ascertainment bias on heterozygosity estimates [18, 19], fixation index (*F*_*ST*_) values and phylogenetic relationships [9] have been reported. Herrero-Medrano et al. [18] and Albrechtsen et al. [15] observed that ascertainment bias affected some populations more than others when studying their genetic diversity with SNP chip data. McTavish & Hillis [9] concluded that both the *F*_*ST*_ and principal components analysis (PCA) estimated from SNP chip data were distorted when ascertainment bias was not accounted for. Principal components analysis is a statistical technique that captures patterns of high dimensional data and projects them into a lower dimensional space, allowing to determine key variables that explain the observations [20, 21]. PCA has been used in many studies to capture genetic structures of populations [22–26]. In contrary to McTavish & Hillis [9], McVean [27] reported that the PCA is less affected by ascertainment bias. He claims that effects of ascertainment bias on PCA are easy to predict and only have little impact on the structuring of populations unless the bias is very severe.

Despite the available proposed schemes and several suggestions made on how to address the issue of ascertainment bias in population genetic analysis [6, 12, 15], there are still challenges on the definite measures to deal with this issue [17]. Clark et al. [14] concluded that it is not always easy to correct for ascertainment bias, success is not guaranteed, and mostly the suggested corrections are not applicable to every study [15]. Most of the suggestions were also tested using simulated data, which may miss out some of the complexities encountered when using real data.

In this study, we tried to assess the impact of ascertainment bias and the efficiency of various strategies to account for it in a chicken diversity panel, which is based on a diverse set of chicken populations for which both pooled WGS data and individual SNP genotype data obtained with a high density SNP array were available. For most of the studied populations, there is no sufficient documentation on the breed history and/or background and we are skeptic that the material used allows to identify the mechanisms causing ascertainment bias. Therefore, we based our primary focus on identifying strategies to mitigate ascertainment bias rather than to do a full analytical (or empirical) study to understand the causes of ascertainment bias. With the SNP genotyping array [10] that was used, the SNP panel was established by selecting a few populations (for details please see the “Methods” section) which are not representative for all the other populations used in our study. In addition, the SNP selection criterion included discarding low minor allele frequency (MAF) SNPs which potentially causes an underrepresentation of SNPs under selection [28]. Criteria used in our study to assess the impact of ascertainment bias and the various strategies to mitigate its effects were similarity of allele frequency spectra, expected heterozygosity, *F*_*ST*_, PCA, distance measures and topologies of phylogenetic trees. In general, the results obtained from the WGS data were considered as the ‘reference standard’ and strategies to correct for ascertainment bias were considered based on how good the WGS-based results were met.

## Methods

### Animals

A total of 42 chicken populations were used in this study. For each of the populations, both whole genome resequencing data based on pooled samples and individual genotype data obtained with a 600 K SNP Affymetrix® Axiom® High Density Chicken Genotyping Array were available. A list of the 42 populations with their abbreviations and population sizes as used in the study is provided in Table 1. Samples used in this study were collected under the umbrella of the SYNBREED project (www.synbreed.tum.de) from chicken fancy breeds in Germany between 2010 and 2012. The collection was completed by samples of two Red Jungle fowl populations, *Gallus gallus gallus* (GGg) and *Gallus gallus spadiceus* (GGsc) taken from previous EU project AVIANDIV (see [29]).

**Table 1:** List of breeds, their abbreviations and sample sizes as used in the study

Breed and abbreviation	Array data (n)	WGS data (n)
Commercial breeds:		
WL_A – White Leghorn line A	20^a^	10^a^
BL_A – Rhode Island Red line A	20^a^	15^a^
BL_D – White Rock line D	20^a^	15^a^
Wild populations:		
GGg – *Gallus Gallus Gallus*	10 (10)	10
GGsc – *Gallus Gallus spadiceus*	9 (10)	9
European populations:		
ABwa – Barbue d’Anvers quail	10 (10)	10
ARsch – Rumpless Araucana black	9 (11)	9
BAsch – Rosecomb Bantam black	10 (10)	10
BKschg – Bergische Crower	10 (22)	10
DZgh – German Bantam gold partridge	10 (10)	10
FZgpo – Booted Bantam millefleur	10 (10)	10
HOxx – Dutch White Crested	10 (7)	10
ITrh – Leghorn brown	10 (10)	10
KAsch – Castilians black	9 (11)	9
KRsch – Creeper black	10 (20)	10
KRw – Creeper white	10 (20)	10
LER11- White Leghorn line R11	9 (13)	9 (1)
OMsschg - East Friesian Gulls silver penciled	10 (10)	10
PAxx - Poland any colour	11 (12)	11
SBsschs - Sebright Bantam silver	10 (10)	10
WTs - Westphalian Chicken silver	10 (10)	10
Asian populations:		
ASrb – Aseel red mottled	10 (10)	10
BHrg – Brahma gold	10 (10)	10
CHgesch – Japanese Bantam black tailed buff	10 (12)	10
CHschw – Japanese Bantam black mottled	10 (19)	10
COsch – Cochin black	10 (11)	10
DLIa – German Faverolles salmon	10 (10)	10
KSgw – Ko Shamo black-red	9 (13)	9
MAxx – Malay black red	10 (21)	10
MRschk – Marans copper black	10 (10)	10
NHL68 – New Hampshire line 68	9 (14)	9 (1)
OFrbx – Orloff red spangled	10 (15)	10
OHsh - Ohiki silver duckwing	10 (10)	10
ORge - Orpington buff	10 (10)	10
SAsch - Sumatra black	9 (11)	9
SEw - Silkies white	10 (10)	10
SHsch - Shamo black	9 (11)	9
SNwsch - Sundheimer light	10 (10)	10
TOgh - Toutenkou black breasted red	10 (11)	10
WYw - Wyandotte white	10 (9)	10
YOwr - Yokohama red saddled white	10 (10)	10
ZCw - Pekin Bantam white	10 (10)	10

n is number, in brackets () are additional individuals added to the population (not present in the other data type)

^a^completely different individuals in the two data sets

For the WGS pooled data, equal amounts of DNA of the individuals of each population were pooled using *PicoGreen®* quantitation assay except for the WL_A. In the case of WL_A, 10 birds were sequenced individually and virtual pooling was performed. Thirty nine of the 42 populations in the WGS consisted of 385 individuals of which 383 were also genotyped individually. The other 3 populations (WL_A, BL_A and BL_D) were commercial lines (see Table 1) and consisted of different individuals in the two data sets. In the array data set, in addition to the 383 individuals, 461 more individuals were added and their distribution is also shown in Table 1. So, when comparisons were made between array and WGS data with commercial breeds included, the 383 plus 461 individuals’ version of array data was used. For the commercial breeds, each breed contained 20 individuals in the array data. In the WGS data, each breed contained 9–10 individuals for the non-commercials and 10–15 individuals for the commercial breeds. The commercial breeds were among the breeds used in the discovery panel for the development of the 600K Affymetrix genotyping array.

Collection of blood samples for this study was performed in accordance with the German Animal Protection Law and was submitted to and approved by the Committee of Animal Welfare at the Institute of Farm Animal Genetics (Friedrich-Loeffler-Institut) and the Lower Saxony State Office for Consumer Protection and Food Safety (No. 33.9-42502-05-10A064).

### WGS data and preparation

Pools of the 42 populations comprising in total 425 individuals were resequenced with 20X target coverage. The sequence reads were aligned to the chicken reference genome (galGal4) [30] using Burrows-wheeler alignment algorithm implemented in BWA [31] and sorted using Samtools [32]. Picard tools were used to mark duplicates and GATK was used for calling the SNPs [33, 34]. For more details on the preparation pipeline see Reimer et al. [35].

### Genotype (array) data and filtering

The initial array data set contained 918 animals and 580, 588 SNPs. SNPs misplaced at wrong chromosomes were removed. The data was then filtered for SNP call rates of >99% and animal call rate of >95% using the SNP & Variation Suite Version (SVS) 8.1 [36] which retained 904 animals and 450, 082 SNPs. From this point, the following SNP filtering pipeline was applied, with number of SNPs left at each step shown in brackets:

1. SNPs with missing positions were discarded (445,428).
2. SNPs that shared the same position on the same chromosome were discarded (e.g. if there were two SNPs sharing the same position, both of them were discarded (445,388).
3. SNPs had to be present in both array and WGS data (21,759 of array SNPs were not found in the WGS data) and only SNPs from chromosome 1–28 were considered (401,420).
4. SNPs were discarded if the reference (and/or alternative) allele of genotype (array) data didn’t match the reference (and/or alternative) allele from the sequence data (401,125).

After the above filtering, a total of 401,125 SNPs remained for further analysis. This set of data was used in assessing allele frequency calling in the pooled sequence data, comparing allele frequencies between the array and WGS data, and assessing how this uncorrected ascertained data affect genetic diversity analysis by being compared to results analyzed from WGS. The array data SNP was converted so that allele A resembled the respective reference allele.

Different filtering schemes were applied to the array dataset (Array_all in Table 2) to be tested for their potential to account for ascertainment bias. More specifically, we applied three different basic filtering principles:

1. LD based SNP pruning, which has been described to partially account for the effects of ascertainment bias. In our study, SNP pruning for LD was done in PLINK v1.9 [37, 38]. The parameters: *indep 50 5 2* were used, whereby *50* is the window size in SNPs, *5* is the window size step (in SNPs) after LD calculation (after LD has been calculated from the 50 SNPs window, and from a pair of SNPs in LD the SNP with lower MAF is removed, the window is shifted 5 SNPs forward and the procedure is repeated), and *2* is the variance inflation factor VIF = 1/(1-r^2^) [39].
2. A second filter applied was to restrict the analysis to SNPs that were found to be polymorphic in the wild chicken populations, which were represented in our study with two populations (GGsc and GGg subspecies).
3. A third filter excluded SNPs with less than 5% MAF. This MAF filtering was done in PLINK v1.9 [37, 38] using the command –maf 0.05.

**Table 2:** Array data set versions with different filtering strategies applied

Given name for data set	Filter/s applied	No of SNPs
Array_all		401, 125
Array_MAF5	Filtered out SNPs with less than 5% MAF	379, 342
GG	Retained only SNPs that are polymorphic in the two *Gallus gallus* wild populations (GGg and GGsc)	289, 390
GG_MAF5	GG and filtered out SNPs with MAF less than 5%	284, 748
Pruned	SNPs were pruned based on LD	122, 006
Pruned_MAF5	Pruned and filtered out SNPs with MAF less than 5%	107, 604
Pruned_GG	Pruned and GG	86, 404
Pruned_GG_MAF5	Pruned_GG and filtered out SNPs with MAF less than 5%	82, 975

These filters were applied alone and in combination, the corresponding filters and resulting data sets are presented in Table 2.

### Allele frequency calling in the pooled sequence data

To investigate the reliability of allele frequency calling in our WGS pooled data, we estimated and compared allele frequencies between array (using all 401,125 SNPs) and WGS pooled data for corresponding loci. To avoid issues relating to sample size [40], only 39 of the 42 populations (with 383 individuals for array data and 385 individuals for WGS data) were used for this comparison, the 3 commercial populations which contained different individuals in the two data sets were excluded. Then we also compared the allele frequencies for each breed between the two sets, this time including also the 3 commercial breeds. We used Pearson’s correlations between estimated allele frequencies of WGS and array data to assess the accuracy of allele frequency calling in the pool WGS data. All allele frequency calculations were based on the alternative allele at each locus. Allele frequencies for the pooled sequences were calculated as the proportion of reads’ counts for the alternative allele at each locus.

### Assessing ascertainment bias in the array data

We randomly sampled 401,125 SNPs in 100 repetitions from the WGS data, computed the average allele frequency spectrum (AFS) and compared it with the AFS of the 401,125 SNPs in the array data.

Genic SNPs of *Gallus gallus* were annotated with Ensembl genes 85 [41] and the proportions of SNPs in genic and non-genic regions were calculated and compared between the two sets. The genic region was defined according to the Ensembl gene definition, comprised of any spliced transcripts with overlapping coding sequence [42]. It was further determined if there are differences in MAF distributions from the genic and non-genic regions in the two data types.

### Assessing the potential effects of ascertainment bias in genetic variation analysis

Within breeds diversity analyses, population differentiation and phylogenetic structure analyses were performed and compared between the WGS data and different versions of the array data. For within breed variation, the expected heterozygosity (*H*_*e*_) was estimated as: *H*_*e*_*= 2p(1-p)*, where *p* represented the allele frequency of the alternative allele [43]. We could not use the observed heterozygosity for comparison since this one was not available for the pooled sequence data.

As a measure of population differentiation, the pairwise fixation index (*F*_*ST*_) between breeds for each locus was estimated as: ${F}_{ST}=\frac{s^2}{\overline{p}\ \left(1-\overline{p}\right)}$ [44]. For the same sample sizes *s*^2^ was calculated as ${\sum}_i{\left(\overset{\sim }{p_i}-\overline{p}\right)}^2/r$ where $\overset{\sim }{p_i}$ is the allele frequency of the *i*^*th*^ population, $\overline{p}$ is the average allele frequency across populations and *r* is the number of populations the *F*_*ST*_ is calculated for. For different sample sizes the *s*^2^ was calculated as ${\sum}_i{n}_i{\left(\overset{\sim }{p_i}-\overline{p}\right)}^2/r\overline{n}$ and $\overline{p}$ calculated as ${\sum}_i{n}_i{\overset{\sim }{p}}_i/r\overline{n}$ where *n*_*i*_ is the sample size of the *i*^*th*^ population and $\overline{n}$ is the mean sample size. The *F*_*ST*_ values were averaged across loci.

Phylogenetic variation between populations in the different data sets was evaluated by means of phylogenetic trees and principal components analysis (PCA). Pairwise genetic distances were estimated using Nei’s standard genetic distance [45]. The pairwise genetic distance matrices of the different array data versions were compared with that of WGS using Frobenius (*F*) distances, which was calculated as ${F}_{A,B}=\sqrt{trace\left(\left(A-B\right)\ast {\left(A-B\right)}^{\prime}\right)}$ [46], where A and B are the two distance matrices to be compared. Since it couldn’t be ruled out that there is a scale effect of the number of SNPs used in the construction of the distance matrix, we sampled 100 replicates from the WGS data with the same number of SNPs as was used in the construction of the array-based matrix in the respective comparison. We then calculated the genetic distances and compared the respective array-based matrix to the 100 replicates of the WGS-based matrices.

The phylogenetic trees were derived from the pairwise distance matrices between the breeds. The ‘Ape’ package in R v3.2.2 was used to compute and construct neighbor joining (NJ) trees [47, 48]. The NJ trees were then compared using their topological distances obtained from two methods:

1. Penny & Hendy [49] consider the topological distance as twice the number of internal branches defining different bipartitions of the tips. Comparisons here are made by counting the number of different partitions resulting from cutting the interior branches of the two trees. Differences in partitions are determined by having one or more different objects (in our case different populations) when the trees are cut at a branch. The topological difference is then calculated by how many partitions need to be changed in order for the two trees to be similar. This method determines how similar objects are grouped together in the two trees based on the partitions. A value of 0 means that cutting the trees at any similar branch point results in similar objects on the partitions of the two trees; therefore, the two trees are considered to have a similar topology. The lower the value, the more similar the two trees are.
2. Billera et al. [50] consider the topological distance as the sum of the branch lengths that need to be erased to have two similar trees calculated as $d=\sqrt{\sum {\left({X}_i-{Y}_i\right)}^2}$, where X and Y are two NJ trees, and *i* is the *i*^*th*^ population in X and Y. *X*_*i*_and *Y*_*i*_ are the branch lengths of the *i*^*th*^ population in trees X and Y respectively. The branch length is described as the amount of evolutionary change [51], and the distance between two populations in one tree is the sum of the branch lengths connecting them. Therefore, if population *i = 1* in tree X and Y has the same branch lengths but population *i = 2* in tree X and Y has different length, the distance between population 1 and 2 in the two trees will be different. This method estimates the difference between the two trees for the *i*^*th*^ population and sums all the differences for every population. A value of 0 means that all pairs of populations have the same branch lengths connecting them in the two trees.

Again these comparisons were made between the different versions of the array data set and the randomly sampled 100 replicates of WGS data and with the same number of SNPs, respectively.

The “ade4” and “stats” packages in R were used to compute the PCA and the packages “factoextra” and “scatterplot3d” for visualizing the results in two dimensional (2D) and three dimensional (3D) respectively [48, 52, 53].

## Results

### Assessing allele frequency calling in pool whole resequencing data

**Fig. 1:**
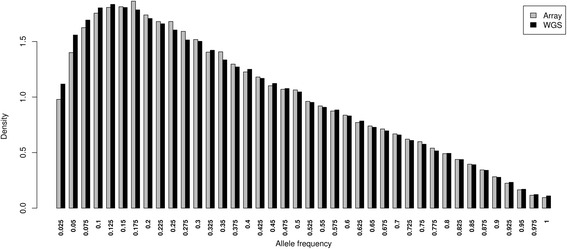
Allele frequency spectrum of array data and corresponding WGS loci for 39 populations

We compared the estimated allele frequency for all SNPs in the ‘Array_all’ data set with the estimates from the pool WGS data at each corresponding locus. The allele frequency spectra of the two data sets were found to be mostly identical (Fig. 1). The proportion of SNPs in the frequency bin 0.025–0.125 was slightly higher in the WGS than the array data while the proportions of SNPs in the bins 0.150–0.3 were slightly higher in the array than the WGS data. A high correlation was obtained between the allele frequencies of the two sets (*r* = 0.983), as well as within the different breeds (ranging from *r* = 0.94 to 0.99).

### Assessing the potential of ascertainment bias in the array genotype data

**Fig. 2:**
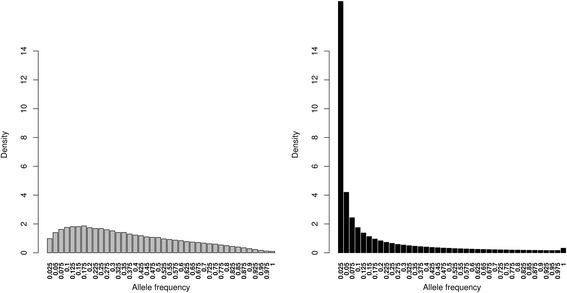
Allele frequency spectrum of array data (left) and WGS data (right) for 39 populations

The allele frequency spectra showed remarkable differences for the two data types (Fig. 2). The array data had very low but increasing numbers of SNPs at allele frequencies between 0 and 0.175 while the WGS had a very high number of rare variants between 0 and 0.025 and SNP numbers decreased with increasing frequencies, with the exception of the last window (which includes the fixation of the derived allele) which was found to be slightly over-represented.

For the individual populations (refer to Additional file 1), the most affected in terms of missing rare SNPs were the Marans copper black (MRschk), Araucana black (ARsch) and the wild GGsc; and the least affected were the European fancy bantam (SBsschs, BAsch, FZgpo and ABwa) breeds, the White Leghorn line R11 (LER11), the Asian long tailed (TOgh and OHsh) breeds and the commercial white layers (WL_A). In the latter, these results have shown to be related to the genetic diversity within these breeds (see *H*_*e*_ estimates below and the discussion thereof).

The proportion of SNPs was 39.6% and 39.9% in genes and was 0.044% and 0.012% in exons for array and WGS data, respectively (see in Additional file 2: Table S1). Differences in (minor) allele frequencies (in genic and non-genic regions) followed a similar pattern to that observed in Fig. 2 whereby rare variants were underrepresented in the array data. The correlations between MAF proportions in genic and non-genic regions were 0.956 and 0.999 in the array and WGS data, respectively. The minor allele frequency of SNPs differed very little between the genic and non-genic regions with the array and sequence data (Additional file 3: Figure S1). From this we concluded that the selection of SNPs in the array was not biased based on their positions in genic or non-genic region, although, differences between the two sets were found to be in the exonic regions whereby the array set had an overrepresentation of SNPs.

**Fig. 3:**
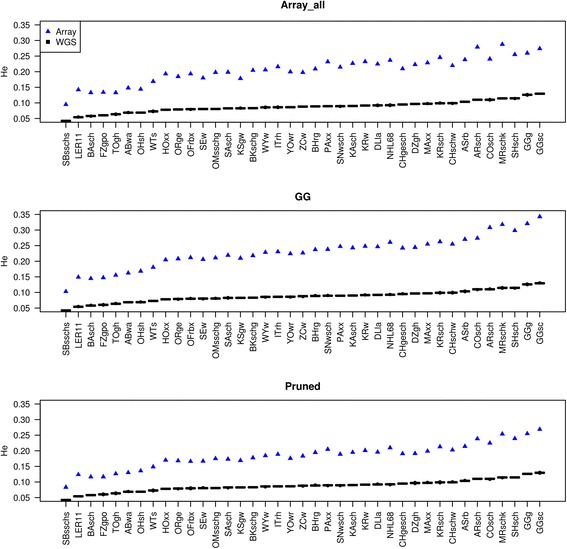
Comparisons of expected heterozygosity (*H*_*e*_) estimates between WGS (boxplot of 100 replicates) and array (Array_all, GG and Pruned) data

Within breed variation was assessed by comparing the expected heterozygosity estimates between the two sets, and the results for the WGS vs. Array_all, GG and Pruned versions of array data are shown in Fig. 3. The versions with MAF filtering barely showed any difference and are therefore not shown. In Fig. 3, we ranked the breeds in ascending order of the estimated *H*_*e*_ in WGS and fitted (for each same breed) the array estimated *H*_*e*_ to observe if it also appears in the same ranking order as the WGS data. The red jungle fowls, which are believed to be the ancestors of domestic chickens are expected to carry more genetic information than found in most of the other populations. When using the WGS data, the highest genetic diversity was observed in the two red jungle fowls (wild: GGsc and GGg) which was not the case with the Array_all data. There was also considerable random fluctuations in the ranking of the breeds in the Array_all data. Tying up these *H*_*e*_ back to the allele frequency spectra of each population, the highly affected breeds in terms of AFS were also more affected in terms of the *H*_*e*_ ranking (estimated with Array_all) and vice versa for the less affected once. The *H*_*e*_ ranking of MRschk and ARsch in the array data was very high compared to the other breeds. Given the allele frequency and *H*_*e*_ estimates, we observed that the breeds which were least affected by ascertainment bias are mainly those with less genetic variability. After filtering the data for SNPs being polymophic in the wild populations (GG) or pruning the SNPs based on LD (Pruned), the maximum diversity in the wild populations was captured and less fluctuations appeared in the ranking order.

In agreement with e.g. [3], (based on microsatellite data) both the commercial brown (BL_A and BL_D) and white (WL_A) layers displayed reduced genetic diversity within the breed (Additional file 3: Figure S2, estimated using the data with 42 populations). The commercial white egg layers, which emerged from a single parental origin, the White Leghorn breed [5, 54], had very low genetic diversity. The brown layers (BL_A and BL_D) with multi-parental origins of Asian and European background had more genetic diversity compared to white layers. Noting that these commercial breeds were part of the discovery panel, we investigated whether the *H*_*e*_ results behaved differently than in other populations when using array data. Unlike the two brown layer lines with elevated *H*_*e*_ ranking when using any of the array data, the white layers didn’t deviate from the WGS *H*_*e*_ ranking when using the array data (Additional file 3: Figure S2). So this makes it difficult to tie the effects of ascertainment bias on *H*_*e*_ estimation to the relatedness of the breeds to the discovery panel breeds. Furthermore, the fact that the commercial lines’ individuals used in the array data are different to those used in the WGS could also be of impact in this context.

When fitting a linear regression of the WGS-based *H*_*e*_ values on array-based *H*_*e*_ values the slope is >2 with all considered data sets (smallest with 2.150 for the LD pruned data, see Table 3 and in Additional file 3: Figure S3) reflecting not only a systematic overestimation of expected heterozygosity from array data, but also a scale effect resulting in an even more severe overestimation for highly heterozygous breeds. While the underrepresentation of low MAF SNPs in the array data compared to WGS data (cf. Fig. 2) provides a good explanation for the observed difference in the average *H*_*e*_, the reason for the scale effect remains to be understood.

**Table 3:** Relationship between the *H*_*e*_ estimates between WGS and the array data sets

	r_s_	Slope
Array_all	0.956	2.233
Array_MAF5	0.957	2.321
GG	**0.985** ^a^	2.770
GG_MAF5	0.984	2.790
Pruned	0.973	**2.150** ^a^
Pruned_MAF5	0.974	2.340
Pruned_GG	0.983	2.675
Pruned_GG_MAF5	0.983	2.717

*r*_*s*_ – Spearman’s rank correlation. Slope – the slope of regression line when the *H*_*e*_ estimates of array data are regressed against those of WGS data

^a^Numbers in bold face represent the best value in the column. These results are based on 39 populations

**Fig. 4:**
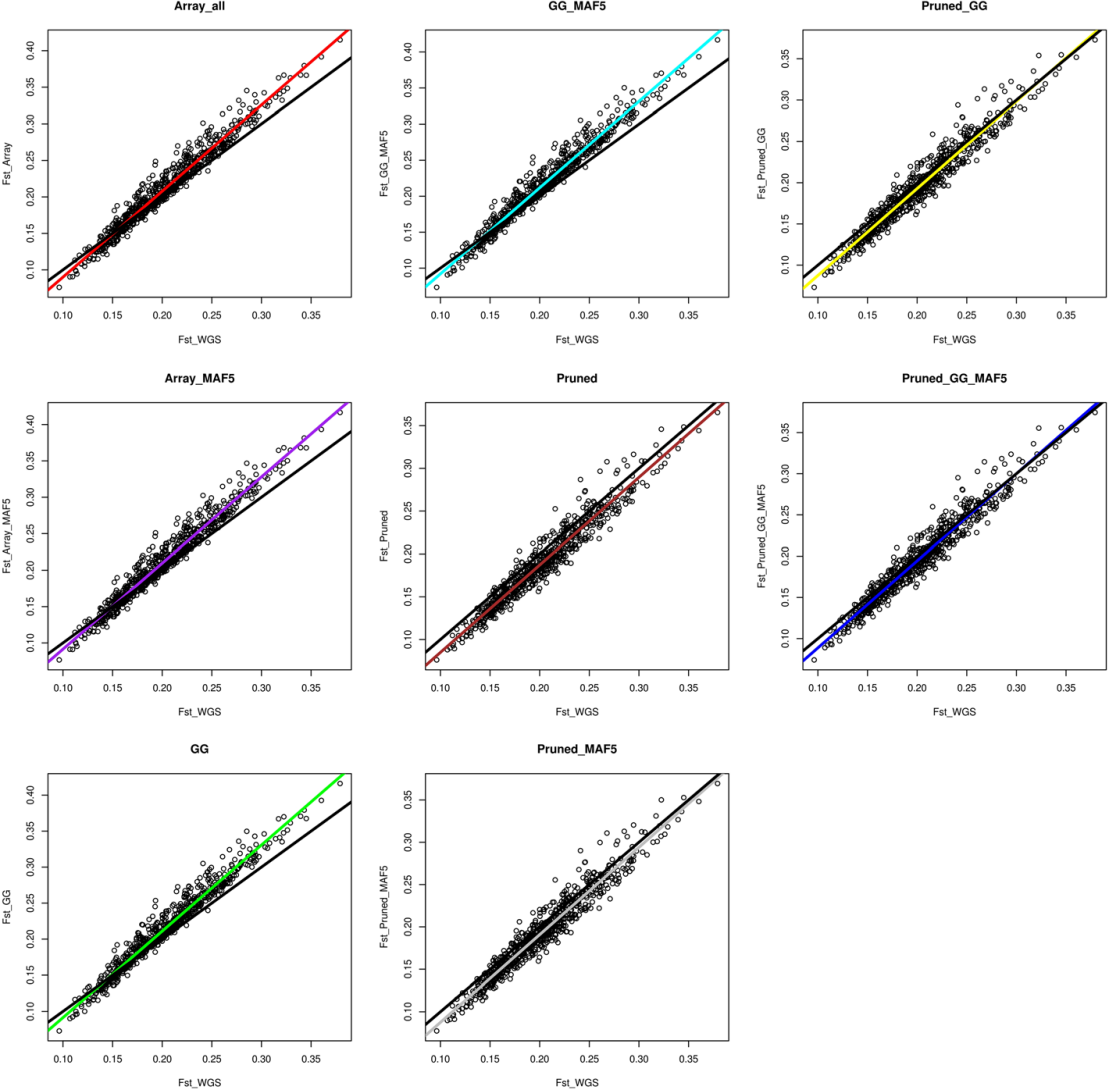
Regressions through the pairwise *F*_*ST*_ values between WGS and array data. Black lines represent the expected identity relationship between the two data sets (with a slope of 1)

A comparison between the estimated pairwise *F*_*ST*_ values of WGS and the different filtered versions of the array data is shown in Fig. 4. The black regression line shows the expected linear relationship between the *F*_*ST*_ of WGS and array where the pairwise *F*_*ST*_ values estimated from the two sets are equal. The Array_all, Array_MAF5 and the versions filtered for being polymorphic in the *Gallus gallus* populations (GG and GG_MAF5) underestimated the *F*_*ST*_ where WGS *F*_*ST*_ was low (0.09 to <0.15) and overestimated the *F*_*ST*_ where WGS *F*_*ST*_ was high (>0.15). The LD pruned versions (Pruned and Pruned_MAF5) and the LD pruned plus polymorphic to *Gallus gallus* populations’ (Pruned_GG and Pruned_GG_MAF5) data sets consistently underestimated the pairwise *F*_*ST*_ values. The regression lines for comparing WGS *F*_*ST*_ and *F*_*ST*_ estimated from the LD pruned versions didn’t cross through the expected regression line, while for versions without LD pruning the regression lines crossed each other. The slopes and regression coefficients (R^2^) of these linear relationships are presented in Table 4. The WGS vs. Pruned data had the lowest R^2^ (0.887), however, with a slope (1.027) closer to 1 compared to the rest of the other array sets. The WGS vs. GG and GG_MAF5 had the highest R^2^ (0.919 for both of them) and yet the highest slope too (1.208 and 1.209 respectively), whereas in this case a better slope (close to 1) is preferred (it justifies the significance of the linear relationship between the pairwise *F*_*ST*_ values estimated from WGS and array data). A combination of filtering SNPs based on LD and retaining SNPs that are polymorphic in the wild populations (GG) improved the R^2^ but compromised the slope.

**Table 4:** The relationship between the *F*_*ST*_ estimates of the WGS and array data

	WGS				
	Slope	R^2^	Regression constant	Standard error (SE)	Residual variance
Array_all	1.179	0.954	−0.028	0.009	0.0001
Array_MAF5	1.183	0.954	−0.027	0.010	0.0001
GG	1.197	**0.959** ^a^	−0.028	0.009	0.0001
GG_MAF5	1.197	**0.959** ^a^	−0.028	0.009	0.0001
Pruned	**1.023** ^a^	0.937	−0.017	0.010	0.0001
Pruned_MAF5	1.033	0.939	−0.016	0.010	0.0001
Pruned_GG	1.055	0.940	−0.018	0.010	0.0001
Pruned _GG_MAF5	1.057	0.941	−0.017	0.010	0.0001

^a^Numbers in bold face represent the best value in the column. R^2^ – regression coefficient. These results are based on 39 populations

Table 5 shows the Frobenius (*F*) distances between the distance matrices of WGS and array (on the diagonal), and the different array sets among themselves. The mean *F* distance between WGS and Pruned data was the lowest (3.152) and highest between WGS and GG_MAF5 data (6.700). A lower *F* distance means two compared distant matrices are more similar. Therefore the pairwise distance matrix of Pruned data is more related to the WGS than the rest of the sets. Among the array versions, the most distant matrices were found between the Pruned version and the GG and GG_MAF5 versions (these GG and GG_MAF5 versions had the highest distances to the matrix of WGS data).

**Table 5:** Frobenius (*F*) distances between distance matrices of WGS and array data

	Array_all	Array_MAF5	GG	GG_MAF5	Pruned	Pruned_MAF5	Pruned_GG	Pruned_GG_MAF5
Array_all	5.312 ± 0.001							
Array_MAF5	0.591	5.889 ± 0.001						
GG	1.239	0.685	6.501 ± 0.001					
GG_MAF5	1.434	0.868	0.200	6.700 ± 0.001				
Pruned	2.230	2.810	3.397	3.596	**3.152**^a^ ± 0.001			
Pruned_MAF5	1.332	1.886	2.447	2.644	0.971	4.115 ± 0.001		
Pruned_GG	1.034	1.530	2.038	2.232	1.417	0.462	4.548 ± 0.002	
Pruned_GG_MAF5	0.811	1.216	1.676	1.867	1.800	0.836	0.329	4.931 ± 0.002

The diagonal is a mean of the *F* distance between the array data set and 100 WGS replicates with the standard errors (SE)

^a^Numbers in bold face represent the best value in the column

**Fig. 5:**
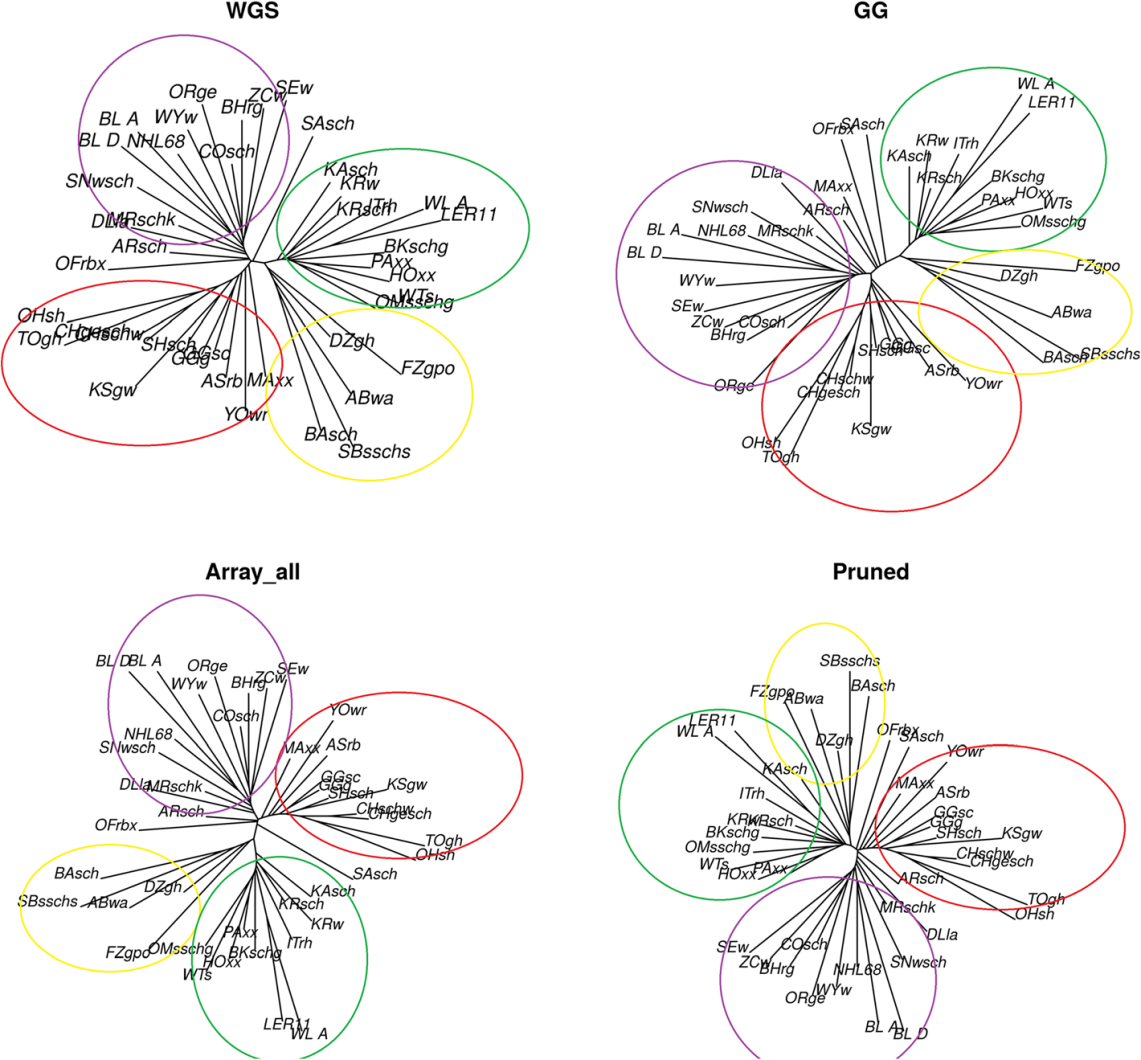
Neighbour joining trees of WGS, Array_all, GG and Pruned data sets

**Fig. 6:**
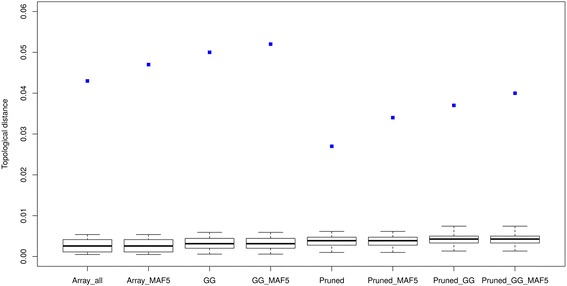
Topological distances between the NJ trees of array and 100 replicates of WGS data based on the Billera method. The boxplots reflect distances between the 100 replicates of WGS and the blue dots are mean distance between the array set and the 100 WGS replicates

**Fig. 7:**
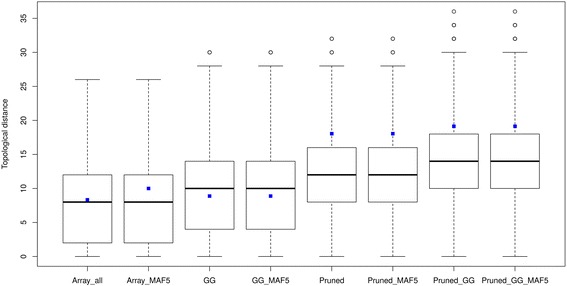
Topological distances between NJ trees of array and 100 replicates of WGS data based on the Penny and Hendy method. The boxplots reflect distances between the 100 replicates of WGS and the blue dots are mean distance between the array set and the 100 WGS replicates

The neighbor joining trees of the WGS, Array_all, Pruned and GG data sets are shown in Fig. 5. Four clusters were identified and circled with different colors and in Additional file 2: Table S2 shows the breeds and their cluster affiliations. Three breeds were outside the clusters and are noted in Additional file 2: Table S2 with an n (not assigned). All the array data sets were able to capture the same clusters as the WGS data in exception of the MAxx population which was not assigned to any cluster when using the GG set while assigned to cluster 2 when using the other sets. Cluster 1 and 2 represent breeds from Asian origin, with cluster 1 grouping the normal sized breeds together and cluster 2 showing a cluster of dwarf birds. Similarly cluster 3 and 4 represents breeds from European origin with normal sized and dwarf birds’ clusters, respectively. From visual inspection, the trees shown displayed many similarities, especially the way breeds were clustered together. To quantify the similarities statistically, we used two different methods [49, 50] to access the topological distances (Figs. 6 and 7) between trees of the WGS and array data sets. Based on the Billera method, the topological distance between the WGS and the Pruned data was the lowest (with distance of 0.027) while it was highest with the GG_MAF5 data (with a distance of 0.052) (detailed in Additional file 2: Table S3). For the WGS and GG data, the distance was 0.050 and for WGS and Array_all data it was 0.043. All the mean topological distances between WGS and the various array sets didn’t fall within the same ranges as the distances between the 100 replicates of WGS (see Fig. 6 and Additional file 2: Table S3). Nonetheless the results show that there is a better relationship between the trees of WGS and the Pruned data than of WGS with any of the other array versions.

Using the Penny and Hendy method, the mean distances between WGS and all the array sets fell within the distance ranges between 100 WGS replicates (see Fig. 7 and in Additional file 2: Table S4). However, the standard errors for the mean distances for all sets’ comparisons were also high. The distances between the WGS and GG, Array_all and their MAF filtered versions were much closer to the median of the 100 replicates. These comparisons of the array and WGS trees based on trees’ partitions using the Penny and Hendy method yielded closer relationships between the two data types. These distances confirmed the visual observation whereby the trees show a relative similar clustering of breeds (Fig. 5). Comparisons across the different array versions showed that Array_all is more related to the GG and both of them are distant to the Pruned data (in Additional file 2: Table S5).

**Fig. 8:**
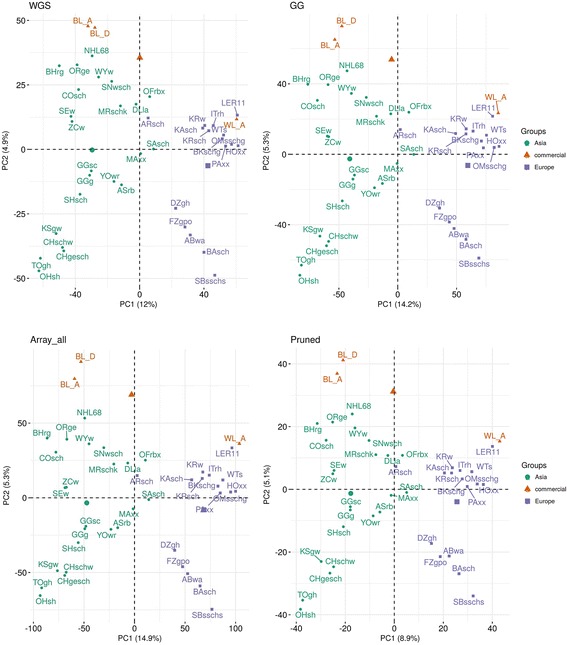
Two dimensional PCA plots of WGS and array (Array_all, GG and Pruned) data

We computed the PCA to see how population structures are captured by the array data compared to the WGS, and visualize the results in 2D and 3D plots. The 2 dimensional PCA plots showed only a very little and hardly noticeable difference between the array sets and the WGS data. Overall all the array versions were able to capture almost similar structures as that of WGS in the two-dimensional PCA. Figure 8 shows the PCA plots of WGS, Array_all, GG and Pruned data sets. In general, the first PC discriminates Asian (left) from European (right) breed types. The first two PCs accounted for 16.9, 20.2, 19.5 and 14% variation in the WGS, Array_all, GG and Pruned data respectively. So, the amount of variation explained by the first two PCs was overestimated with Array_all and GG data, and underestimated with Pruned data. The 3rd PC in these sets still seemed to capture a reasonable amount of variation very close to the same amount captured by the 2nd PC (see in Additional file 3: Figure S4). Visually, the 3D plots showed at least some noticeable, but still small differences in the population structuring compared to the 2D plots.

## Discussion

When assessing allele frequency calling in the pooled WGS data, high correlations were obtained between the allele frequencies estimated with the Array_all’ data set and pool WGS data set at each corresponding locus and very slight differences between the allele frequency spectra, we conclude that the estimation of allele frequencies from pooled sequences is sufficiently reliable. When comparing the AFS from the two datasets (not based on corresponding loci), the array dataset severely underrepresented the rare SNPs (Fig. 2). This confirmed the already known findings of other studies on ascertained SNP data e.g. [9, 14, 15] and therefore suggests a risk for an ascertainment bias in array-based analysis of the chicken biodiversity panel.

To investigate the effects of ascertainment bias and strategies to mitigate its effects, we performed further genetic diversity analyses using the different filtered (LD based pruned, SNPs polymorphic to the GGsc and GGg populations and MAF filtering) versions of the array data and the results were compared with that obtained from the WGS data. LD based pruning of SNPs has been used in several studies presumed to produce reasonable genetic diversity comparisons between breeds [25, 55, 56]. The basic idea of LD based pruning is to remove markers which are highly correlated with other markers within a given window, leaving markers in the set with low LD to each other. This is efficient to remove the multicollinearity effects, which may result in overestimation of effects of SNPs due to highly correlated SNPs. For example, pairwise relatedness can be overestimated if the SNPs are highly correlated. LD based pruning is believed to be very effective when estimating differentiation measures between populations e.g. genetic distances, inbreeding coefficient, kinships and PCA [57].

Filtering of SNPs based on being polymorphic in wild populations not used in the SNP discovering process was discussed as a possibility to reduce ascertainment bias effects in the European Union project (supported from the European Commission) “GLOBALDIV” (http://www.globaldiv.eu/) (not published). The idea was to use most original population within the same species or even a closely related species for selecting markers to be used in diversity studies in order to reduce the possible overestimation of diversity in the discovery panel populations.

Filtering of SNPs with less than 5% MAF is a common practice in quality control of SNP data because of concerns about lower genotyping rates, accuracy of genotype calling or perception about statistical conclusions that comes from analyzing such SNPs [58]. This filtering however will have consequences, there might be significant information behind these rare SNPs and removing them might hinder the chance of discovering such information [16].

Herrero-Medrano et al. [18] found that SNP chip data underestimated heterozygosity (both observed and expected) compared to next generation sequencing data. While Clark et al. [14] obtained higher heterozygosity estimates with ascertained HapMap data, the heterozygosity estimates were lowered after correcting for the bias. In our study, using the array data led to a systematic overestimation of the expected heterozygosity compared to WGS data. However, array data provided a very similar ranking of the breeds, as demonstrated by Spearman’s rank correlations between 0.956 (for Array_all) and 0.985 (for GG, see Table 3). Pruning SNPs based on LD resulted in a reduced overestimation of *H*_*e*_ compared to the other filters and improved the relationship with the WGS results slightly.

Estimating *F*_*ST*_ from the raw array data or with filtering for SNPs found in the wild populations resulted in inconsistence (i.e. underestimation of *F*_*ST*_ where WGS *F*_*ST*_ was low and overestimation the *F*_*ST*_ where WGS *F*_*ST*_ was high) estimates. These inconsistencies may cause misinformed conclusions on the actual differentiation among the populations. In a related study, ascertainment bias has shown to result in higher *F*_*ST*_ values from ascertained SNP data when compared with WGS data [6]. Albrechtsen et al. [15] observed only a small difference in *F*_*ST*_ estimates between SNP chip and resequencing data. But when populations were less related to the ascertained panel, the *F*_*ST*_ estimates increased due to ascertainment bias. They therefore concluded that the bias is dependent on how the investigated populations are related to the ascertainment sample. The array used in our study was developed using several experimental and commercial broiler and layer lines [10]. Due to the multi-breed background of this discovery panel, it is challenging to relate each population to all of these discovery panel populations (including the ones that we didn’t use in this study) in order to come up with a conclusion of whether the relatedness of these populations to the discovery populations affect their *F*_*ST*_ estimates. Additionally, similar to what we have observed with the *H*_*e*_ comparisons, the two commercial layers which we used in our study, were also affected differently (results not shown). This suggests that the effects of ascertainment bias on *F*_*ST*_ estimation in these data sets were very similar independent of whether the populations are more or less related to the discovery panel populations. The LD based pruned SNP data underestimated pairwise *F*_*ST*_ values between breeds, however in a consistent manner and thus should still be preferred over the other filtering strategies.

The clustering of populations by using both PCA and NJ trees is less affected by ascertainment bias. Even thou quantifiable measures such as Frobenius distances (for comparing the distance matrices of the two data types) and topological distances (for comparing the NJ trees) showed that the LD pruned data versions had a better relationship with the WGS data, the NJ trees computed from all array sets displayed similar clusters to the one computed with the WGS data. Ascertainment bias is expected to have limited and predictable effects on PCA. This is according to the in-depth explanation of the underlying processes, including migration, geographical isolation, and admixture in interpreting PCA projections explained by Mcvean [27]. Projections of PCA from SNP genotype data are expected to be similar to PCA projections from WGS data unless the SNP discovery panel is very strongly biased [27]. This expectation was proven truthful in our study where all array data versions (even the Array_all) were found to exhibit structures which were visually very close to the ones obtained from WGS data.

In general, MAF filtering had very little or no effect in all comparisons done, and when its effect was noticeable it actually tended to worsen the results. Tabangin et al. [58] oppose discarding low MAF SNPs with the conception that it will inflate false positives results. Our results also discourage the MAF filtering to consequently study diversity.

Quite a number of studies ([6, 8, 9, 11, 12, 59], among others) on ascertainment bias in genetic studies provide a very good background and insight on the topic. However in most of these studies, the conclusions made on ascertainment bias and its effects on genetic analysis were based on simulated or limited real data. When investigating genome-wide genetic diversity in cattle breeds with SNP data, Edea et al. [60] also investigated the effects of ascertainment bias and most of our results are in agreement with their findings. Furthermore we overcame the shortfalls that were not looked into in their study (i.e. we looked at more possible filtering options, we used WGS as a reference standard and our results discourages the MAF filtering)**.** To the best of our knowledge this paper presents so far the largest study on how different filtering strategies accounts for the effects of ascertainment bias in diversity studies, using real SNP genotype and WGS data. Some of our results (e.g. the only marginal difference between PCA from SNP genotype and WGS data) differ from what was claimed based on simulated (ascertained and non-ascertained) data (e.g. [12]).

Limitations of this study are due to the use of pooled WGS data with a limited number of individuals (9–15 per population) and with 20X coverage only. Due to this, low MAF SNPs may still be missed and some measures, like observed heterozygosity and other inbreeding-related metrics, are not available for the WGS data. Nonetheless, the comparisons between the AFS of WGS and array data based on corresponding loci (Fig. 1) has shown that estimated rare SNPs were a bit higher in the pooled sequence data than in array data therefore, implying a better detection of rare SNPs by sequence pooling (which are missed by the array data). Given these limitations, the pooled WGS data may not completely reflect all aspects of the true diversity of the studied breeds in a comprehensive way, but still our results allow a fair assessment of ascertainment bias and potential mitigation strategies for a number of relevant quantities.

## Conclusions

Using the array genotype data as it is to study genetic diversity of different populations without any accountability measure for ascertainment bias runs the risk of getting misleading results. This study provides insights of how the effects of ascertainment bias can be minimized through appropriate SNP filtering strategies. A variety of populations were represented in our data, comprising both possibly close and distant to the populations in the discovery panel. The LD based pruning of SNPs has proven to yield consistent results which are highly comparable to those obtained from whole genome sequence data for the various populations used in this study in all the results. So, even though it doesn’t fully account for ascertainment bias, the effects remain rather limited and are systematic and, by this, predictable. The other filtering strategies showed to be affected differently with some of the criteria (e.g. *F*_*ST*_ values between populations) and therefore may lead to inconsistent conclusions. Overall pruning of SNPs based on LD outperformed the other filtering strategies and is recommended for practical applications.

## Additional files

Additional file 1: Zip file containing allele frequency spectrum figures of each population. (ZIP 11230 kb)

Additional file 2: Table S1. Proportion of SNPs in genic and non-genic in WGS and array data. **Table S2.** Population clusters. **Table S3.** Topological distances between NJ trees of WGS and array data based on Billera method. **Table S4.** Topological distances between NJ trees of WGS and array data based on Penny and Hendy method. **Table S5**. Topological distances among the array versions. (DOCX 25 kb)

Additional file 3: Figure S1. Comparion of MAF between genic and non-genic regions in array (left) and WGS (right) data. **Figure S2.** Comparisons of expected heterozygosity (*H*_*e*_) estimates between WGS (boxplot of 100 replicates) and array (Array_all, GG and Pruned) data, for all 42 populations. **Figure S3.** Expected heterozygosity (*H*_*e*_) estimated with array vs. WGS data for the 39 populations. **Figure S4.** Three dimensional PCA plot of A) WGS, B) GG and C) Pruned array data. (PDF 761 kb)

Additional file 4: The ENA accession numbers of all sequence reads used in the WGS data. (XLSX 10 kb)

## Abbreviations

AFS: Allele frequency spectrum
*F*: Frobenius
*F*_*ST*_: Fixation index
*H*_*e*_: Expected heterozygosity
LD: Linkage disequilibrium
MAF: Manor allele frequency
NJ: Neighbor joining
PC: Principal component
PCA: Principal components analysis
SNP: Single nucleotide polymorphism
SVS: SNP Variation Suite
Synbreed: Synergistic Plant and Animal Breeding
WGS: Whole genome sequence
