# Supplementary figures and images for: Efficiency of different strategies to mitigate ascertainment bias when using SNP panels in diversity studies
Source: BMC Genomics. 2018 Jan 5;19:22. doi: 10.1186/s12864-017-4416-9 (PMC5756397; doi:10.1186/s12864-017-4416-9)

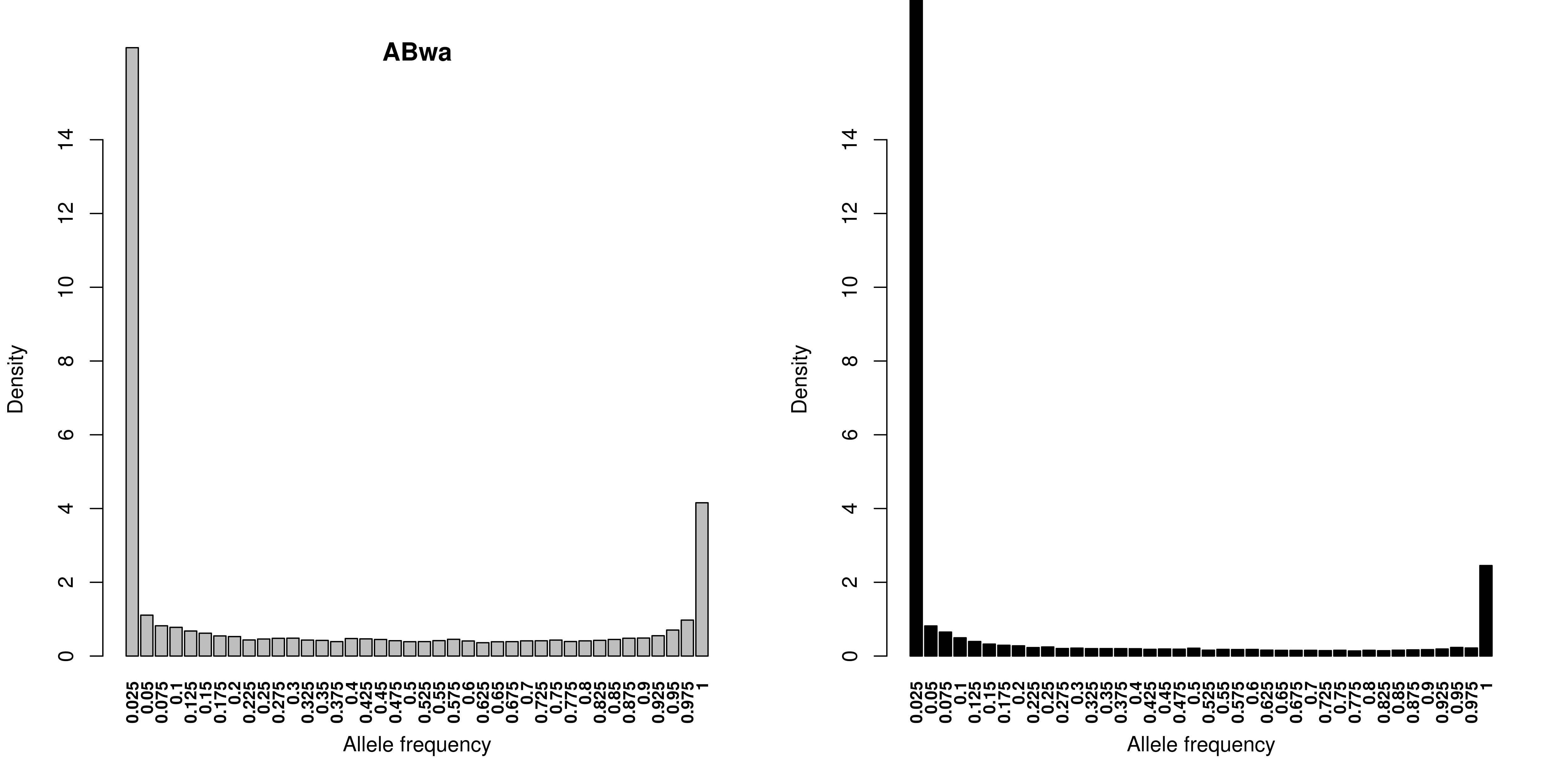

Supplement: Supplementary file 1 — Zip file containing allele frequency spectrum figures of each population. (ZIP 11230 kb) [file 12864_2017_4416_MOESM1_ESM.zip › additional_1 - Copy/AFS_array_WGS_ABwa.tif]

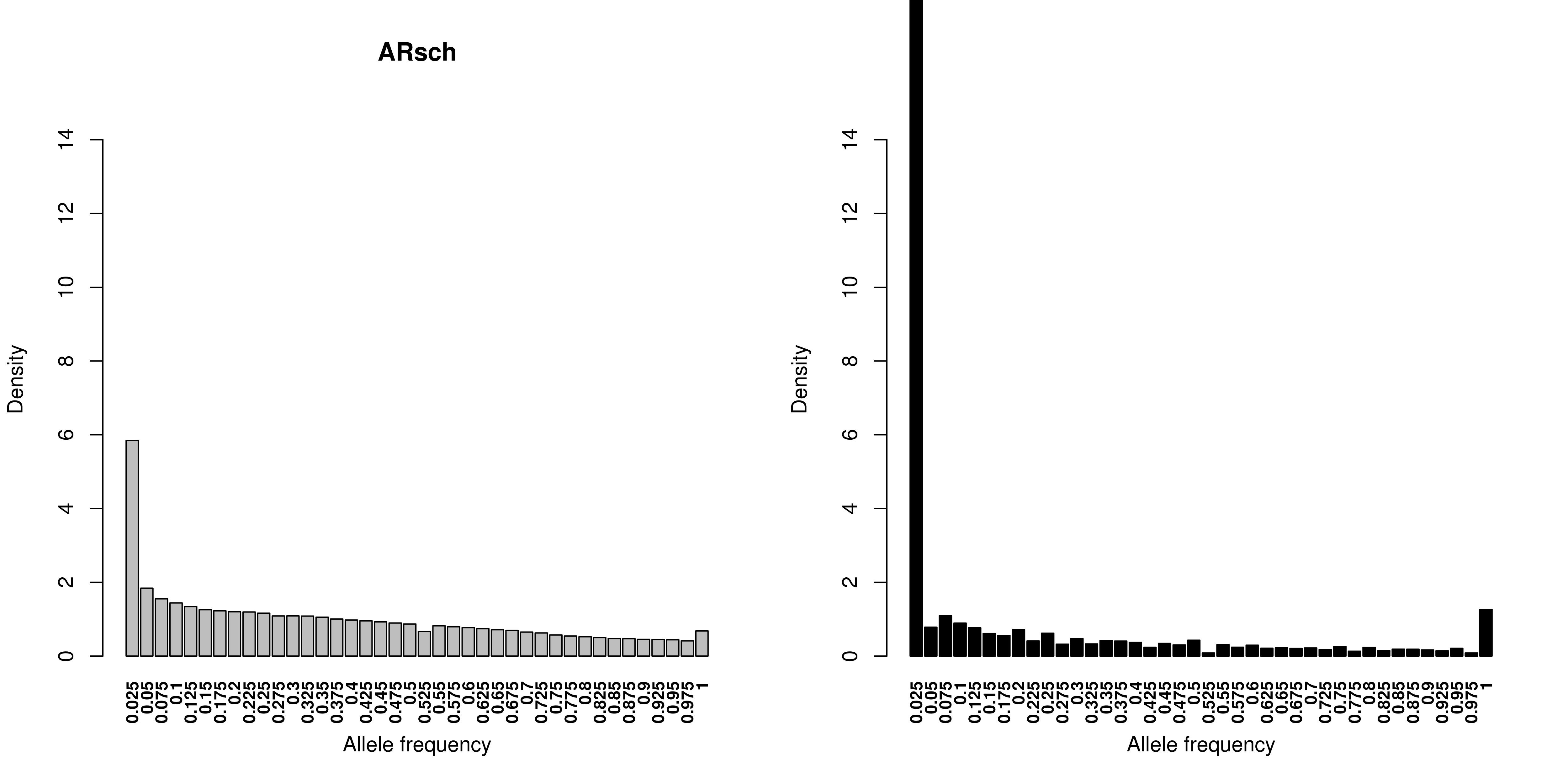

Supplement: Supplementary file 1 — Zip file containing allele frequency spectrum figures of each population. (ZIP 11230 kb) [file 12864_2017_4416_MOESM1_ESM.zip › additional_1 - Copy/AFS_array_WGS_ARsch.tif]

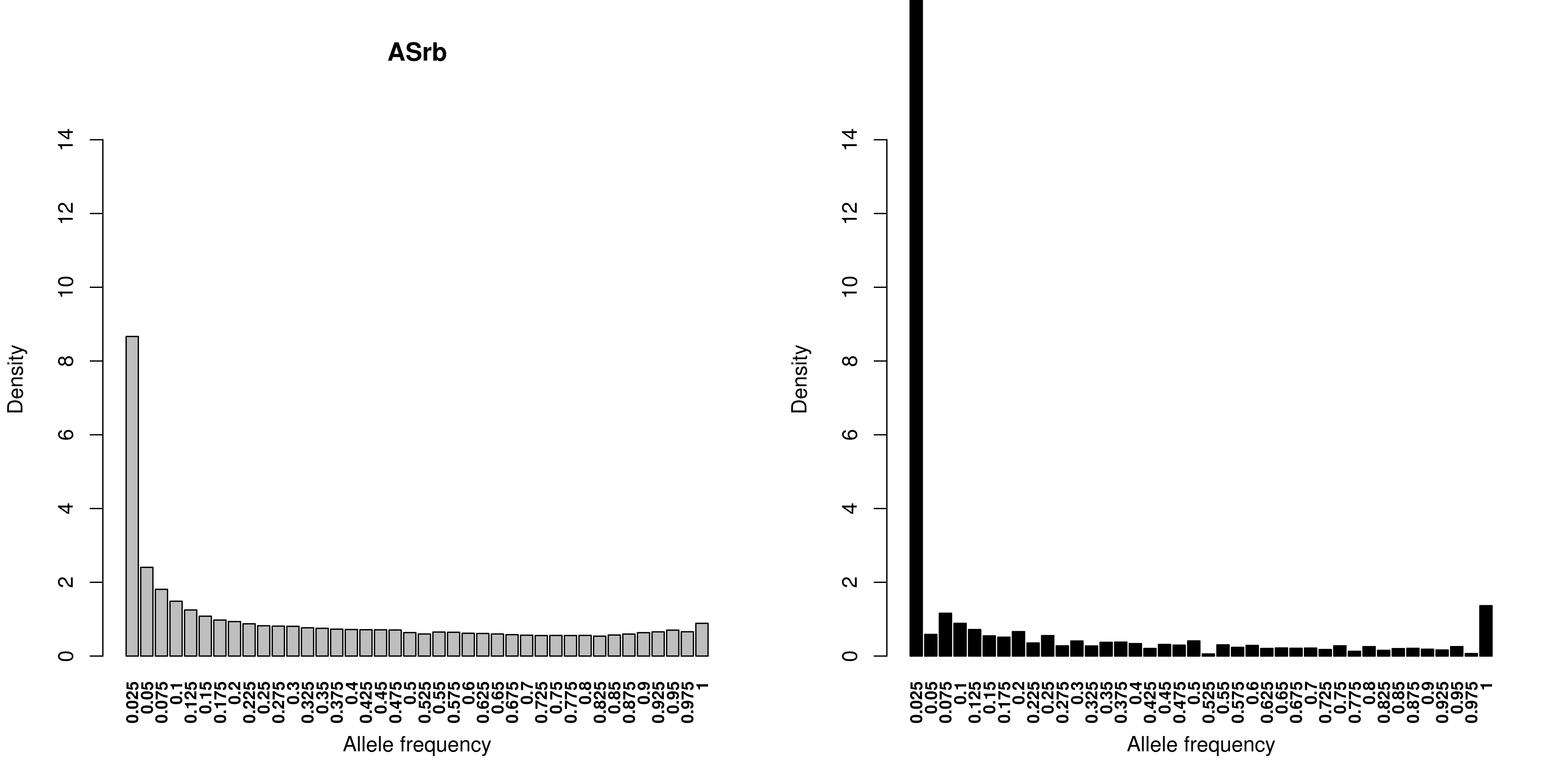

Supplement: Supplementary file 1 — Zip file containing allele frequency spectrum figures of each population. (ZIP 11230 kb) [file 12864_2017_4416_MOESM1_ESM.zip › additional_1 - Copy/AFS_array_WGS_ASrb.tif]

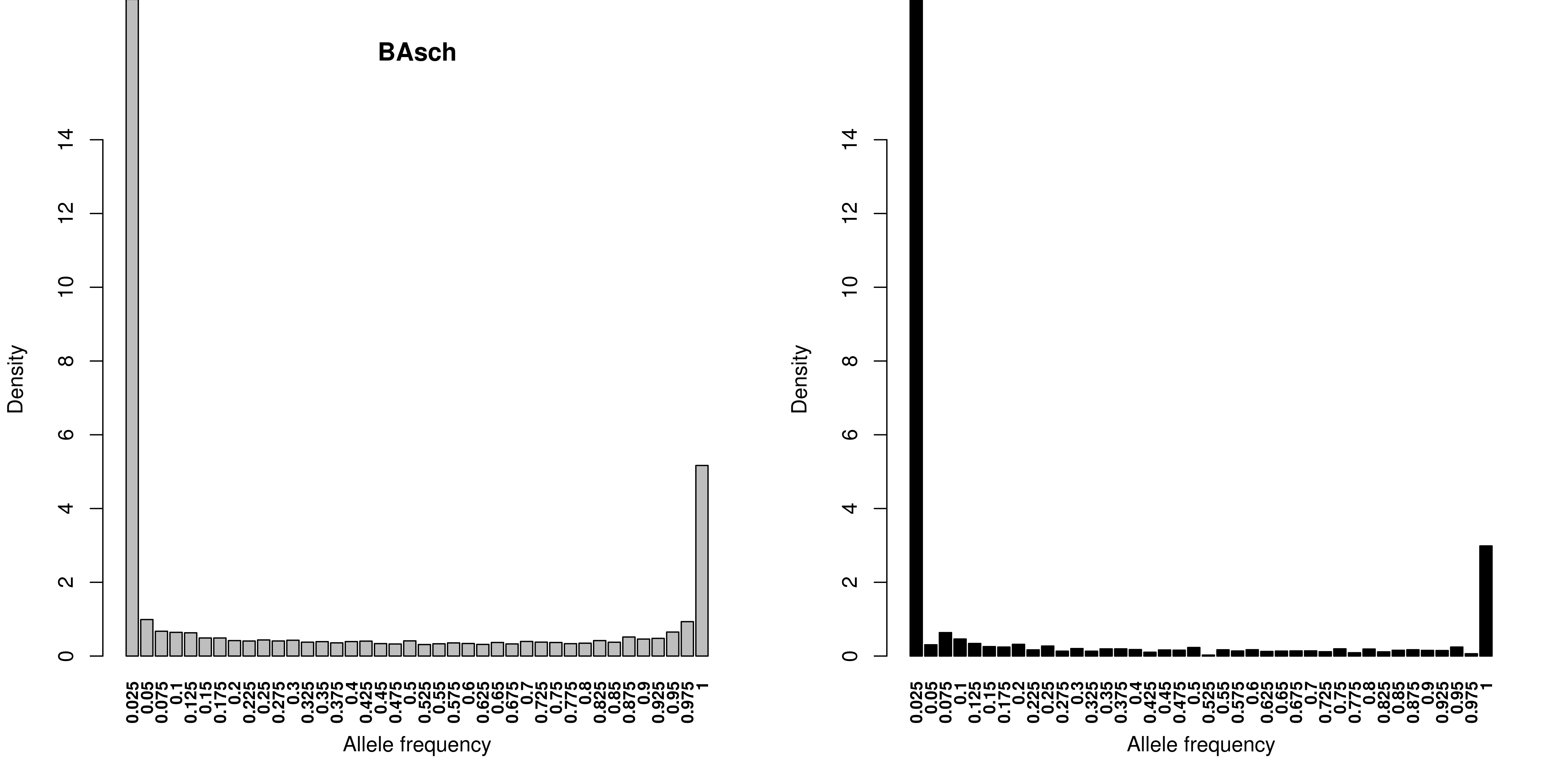

Supplement: Supplementary file 1 — Zip file containing allele frequency spectrum figures of each population. (ZIP 11230 kb) [file 12864_2017_4416_MOESM1_ESM.zip › additional_1 - Copy/AFS_array_WGS_BAsch.tif]

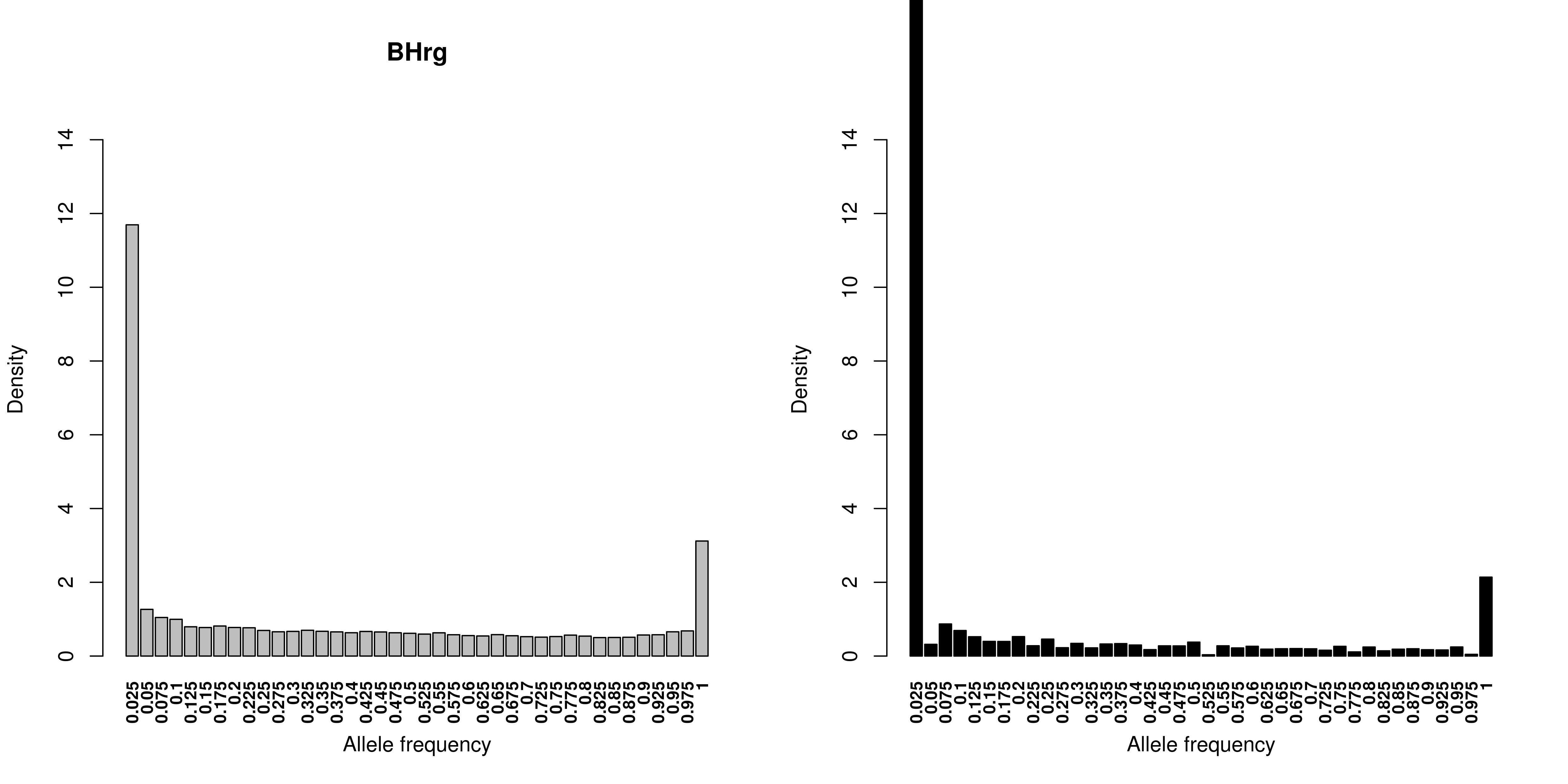

Supplement: Supplementary file 1 — Zip file containing allele frequency spectrum figures of each population. (ZIP 11230 kb) [file 12864_2017_4416_MOESM1_ESM.zip › additional_1 - Copy/AFS_array_WGS_BHrg.tif]

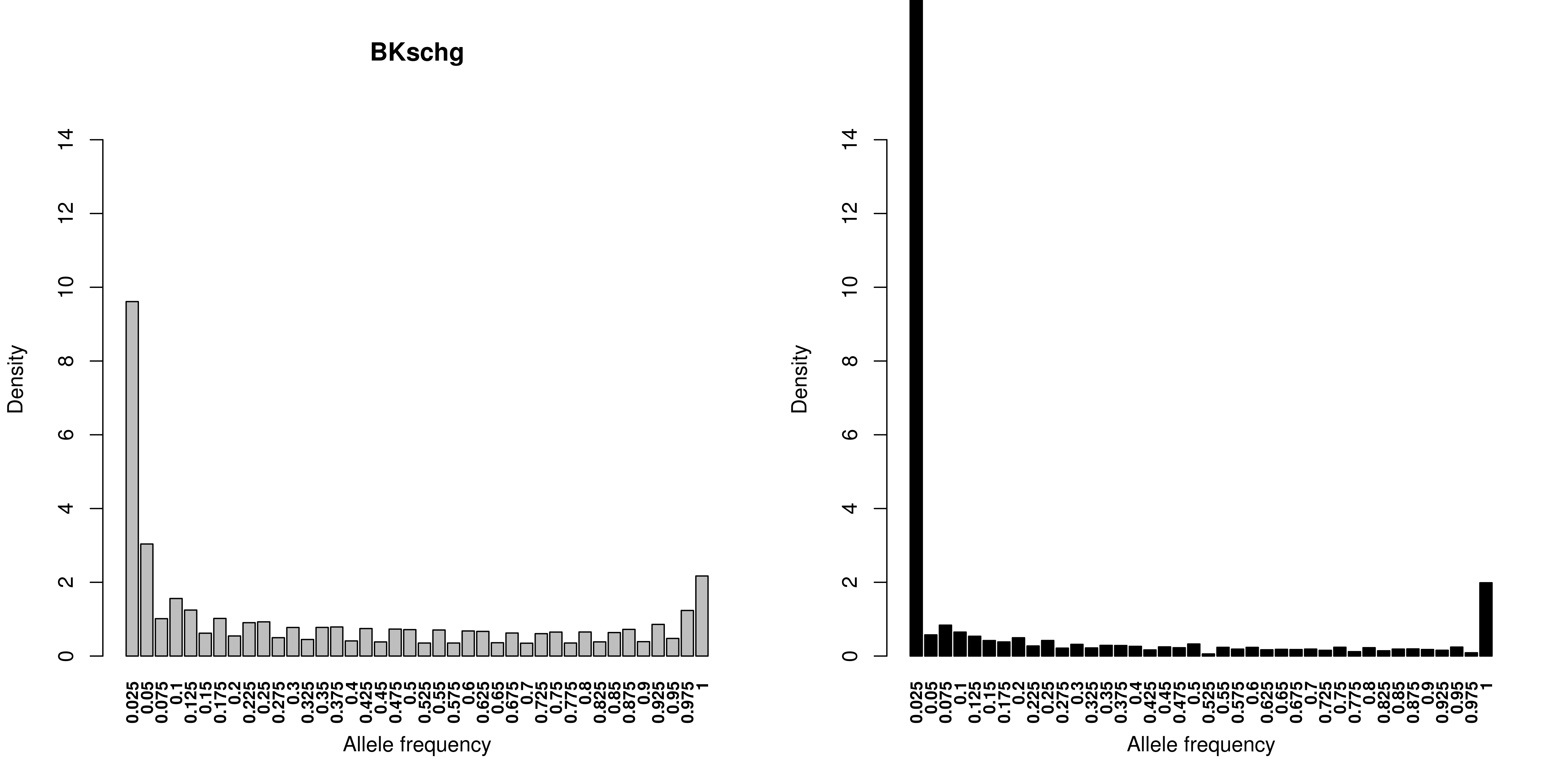

Supplement: Supplementary file 1 — Zip file containing allele frequency spectrum figures of each population. (ZIP 11230 kb) [file 12864_2017_4416_MOESM1_ESM.zip › additional_1 - Copy/AFS_array_WGS_BKschg.tif]

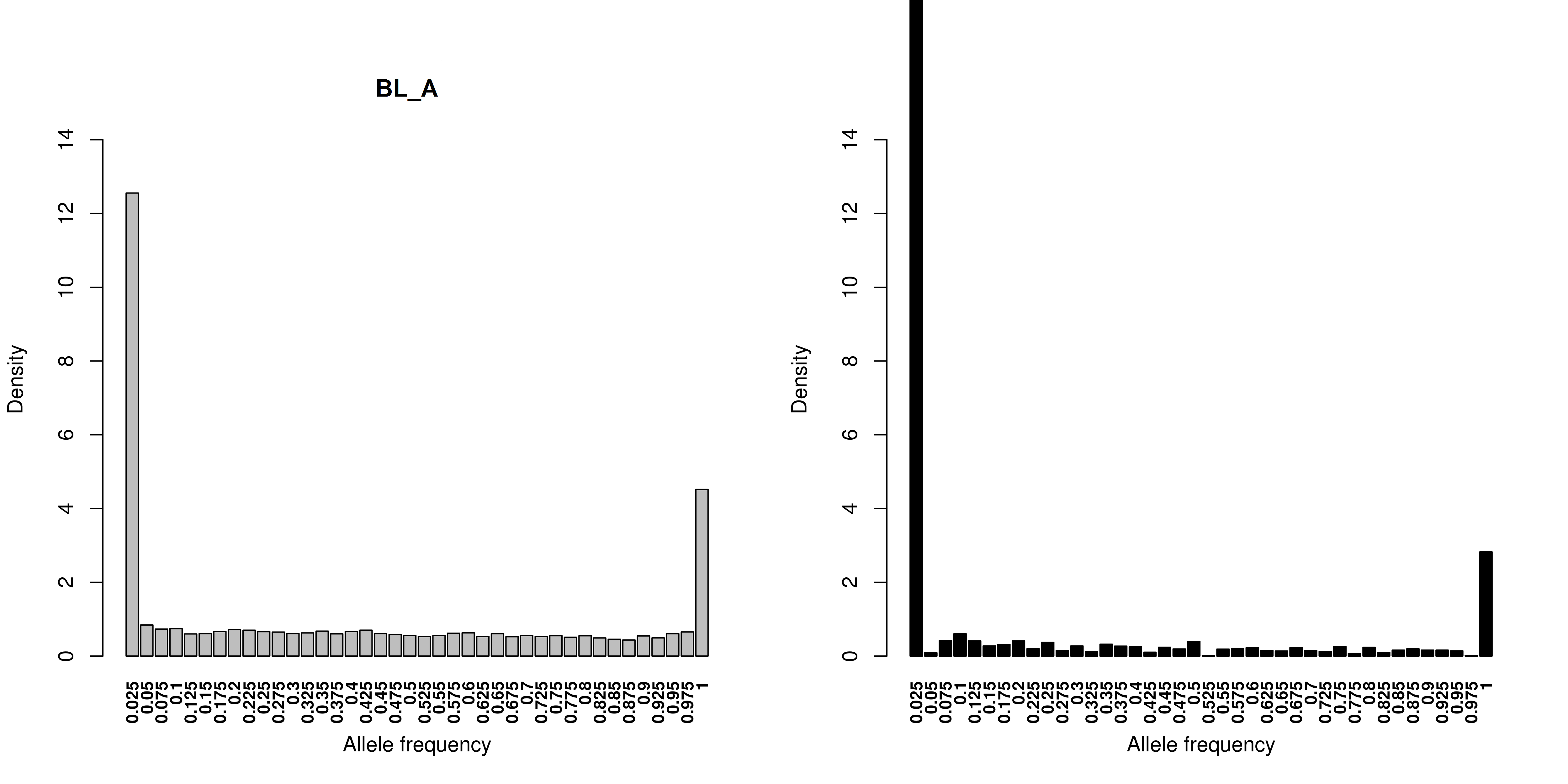

Supplement: Supplementary file 1 — Zip file containing allele frequency spectrum figures of each population. (ZIP 11230 kb) [file 12864_2017_4416_MOESM1_ESM.zip › additional_1 - Copy/AFS_array_WGS_BL_A.tif]

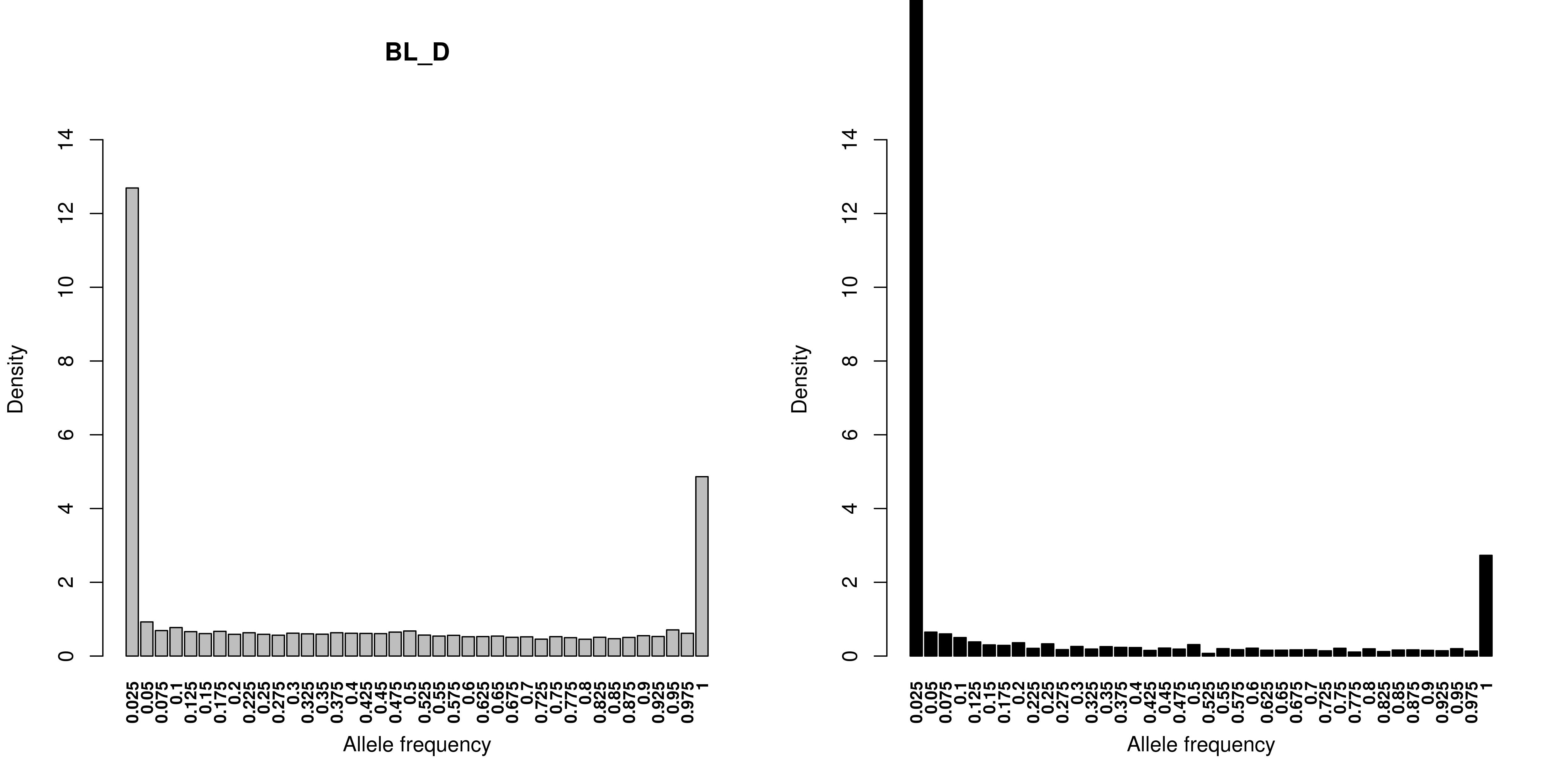

Supplement: Supplementary file 1 — Zip file containing allele frequency spectrum figures of each population. (ZIP 11230 kb) [file 12864_2017_4416_MOESM1_ESM.zip › additional_1 - Copy/AFS_array_WGS_BL_D.tif]

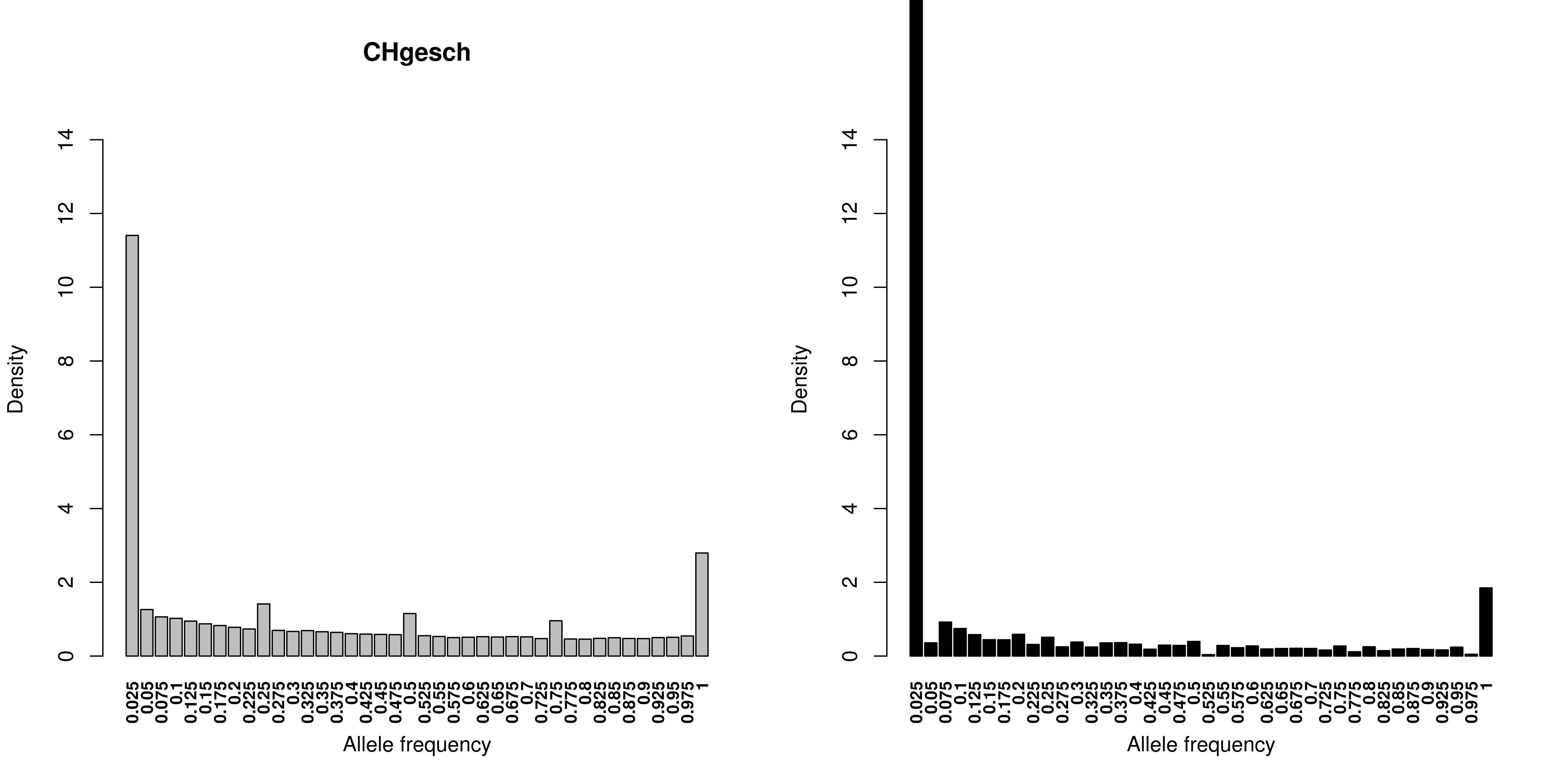

Supplement: Supplementary file 1 — Zip file containing allele frequency spectrum figures of each population. (ZIP 11230 kb) [file 12864_2017_4416_MOESM1_ESM.zip › additional_1 - Copy/AFS_array_WGS_CHgesch.tif]

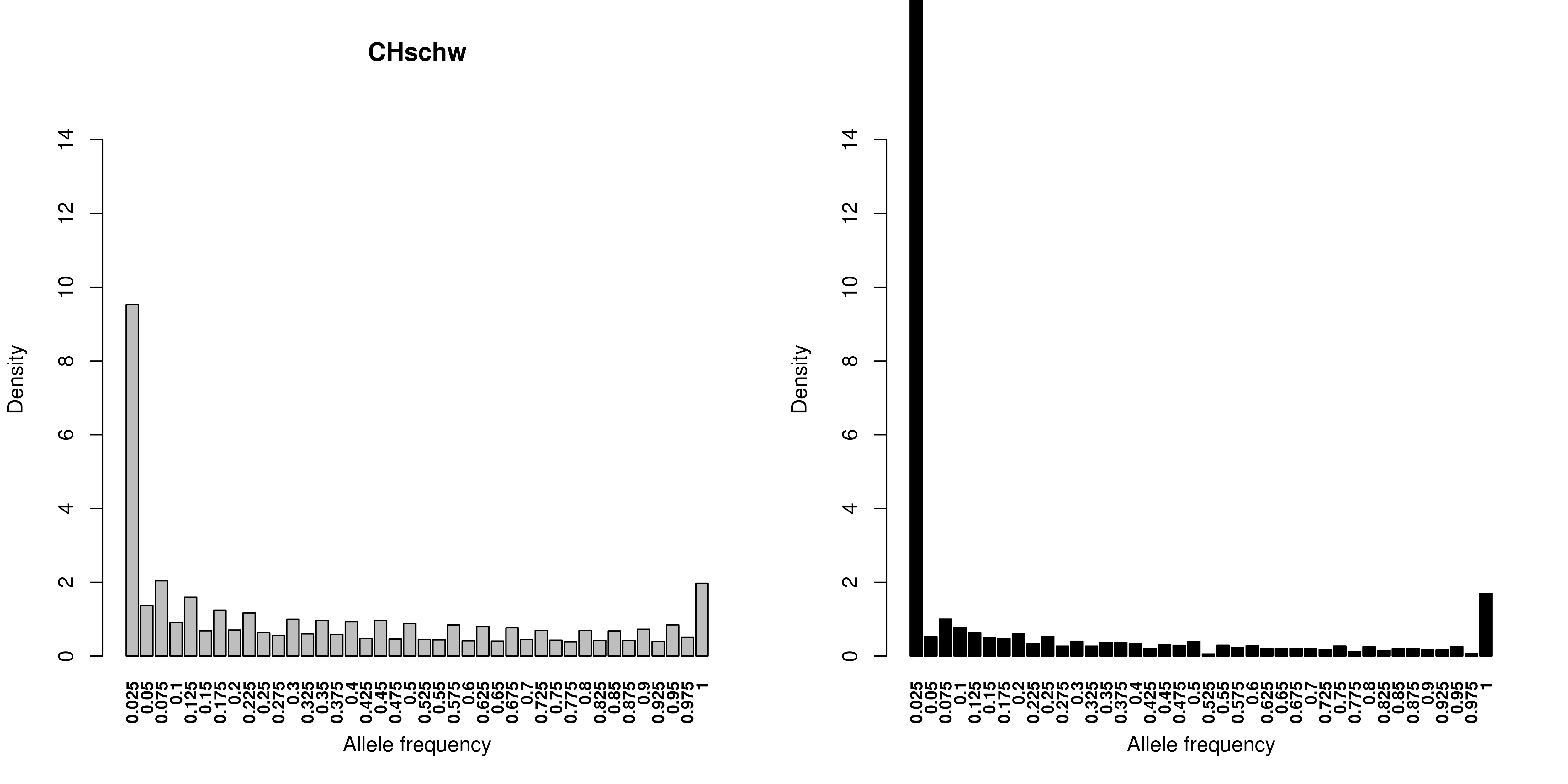

Supplement: Supplementary file 1 — Zip file containing allele frequency spectrum figures of each population. (ZIP 11230 kb) [file 12864_2017_4416_MOESM1_ESM.zip › additional_1 - Copy/AFS_array_WGS_CHschw.tif]

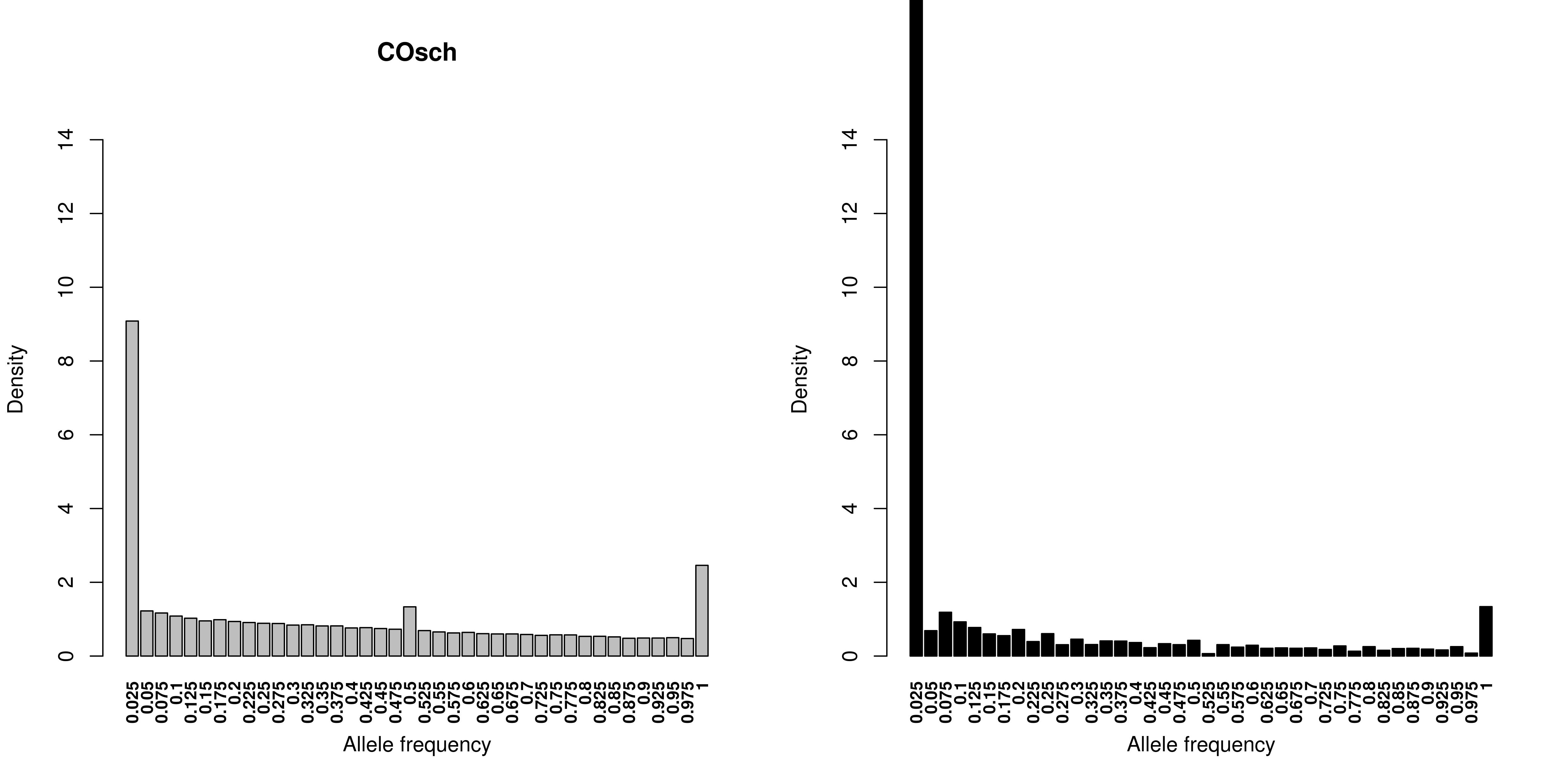

Supplement: Supplementary file 1 — Zip file containing allele frequency spectrum figures of each population. (ZIP 11230 kb) [file 12864_2017_4416_MOESM1_ESM.zip › additional_1 - Copy/AFS_array_WGS_COsch.tif]

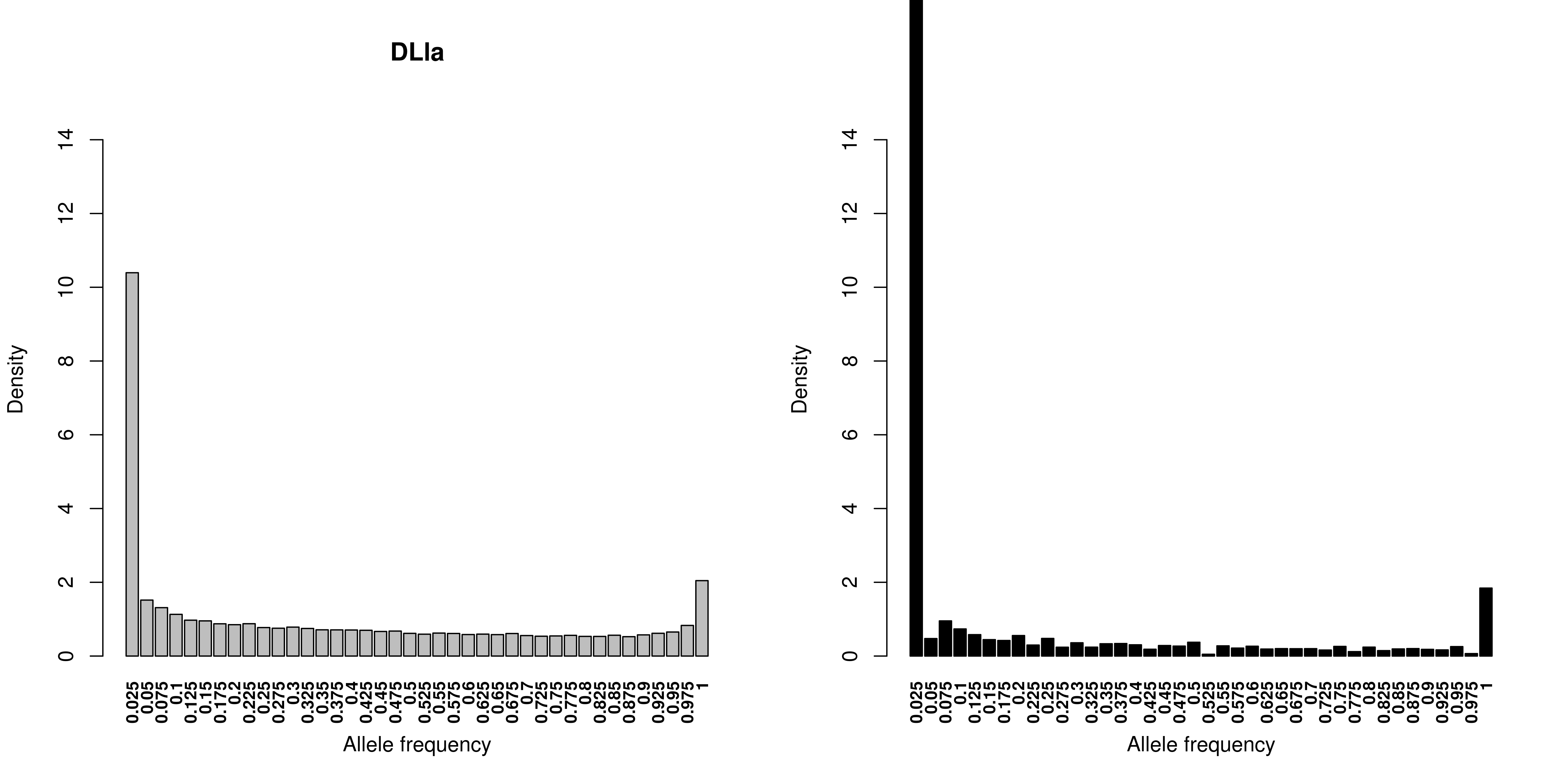

Supplement: Supplementary file 1 — Zip file containing allele frequency spectrum figures of each population. (ZIP 11230 kb) [file 12864_2017_4416_MOESM1_ESM.zip › additional_1 - Copy/AFS_array_WGS_DLla.tif]

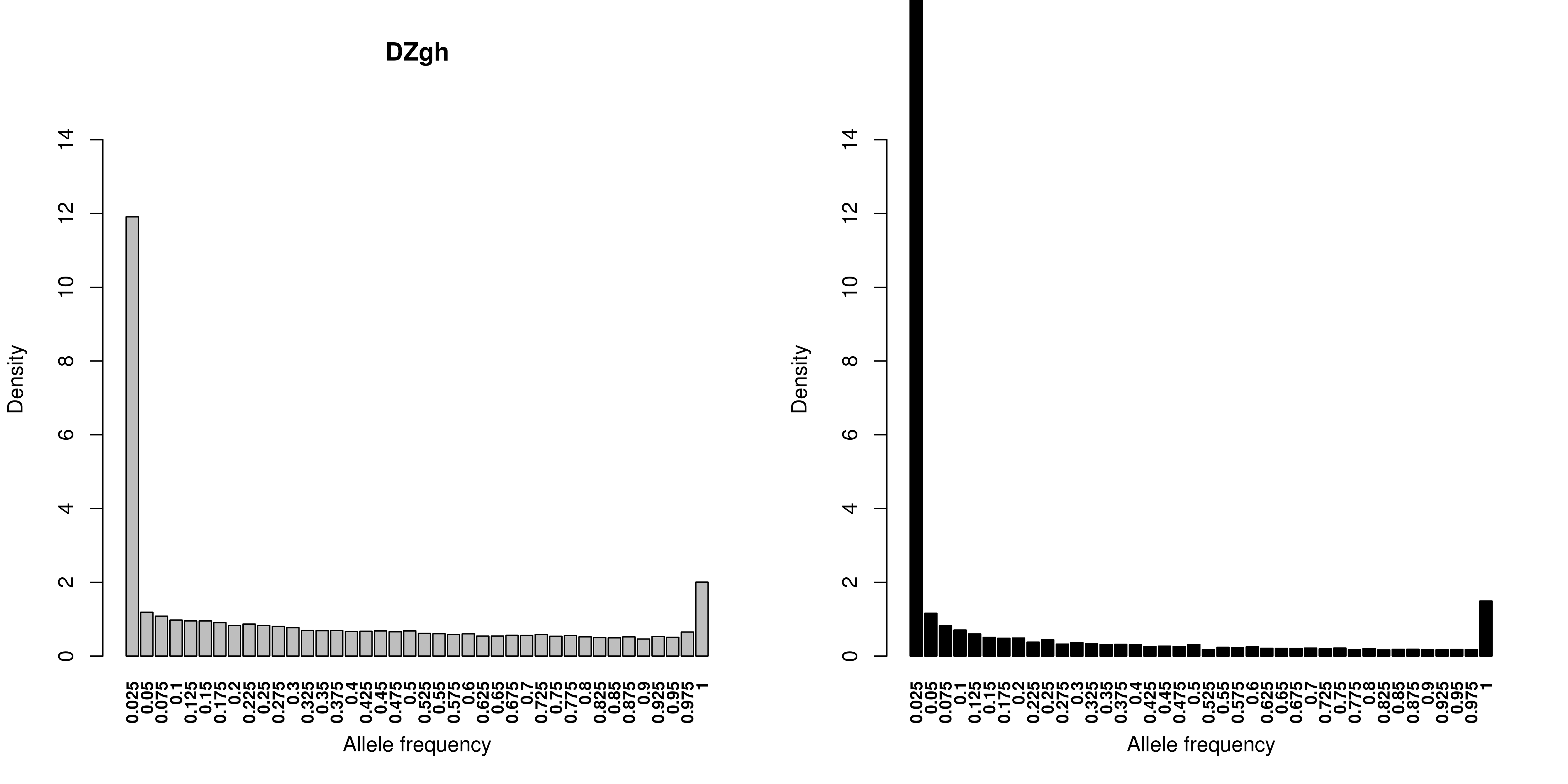

Supplement: Supplementary file 1 — Zip file containing allele frequency spectrum figures of each population. (ZIP 11230 kb) [file 12864_2017_4416_MOESM1_ESM.zip › additional_1 - Copy/AFS_array_WGS_DZgh.tif]

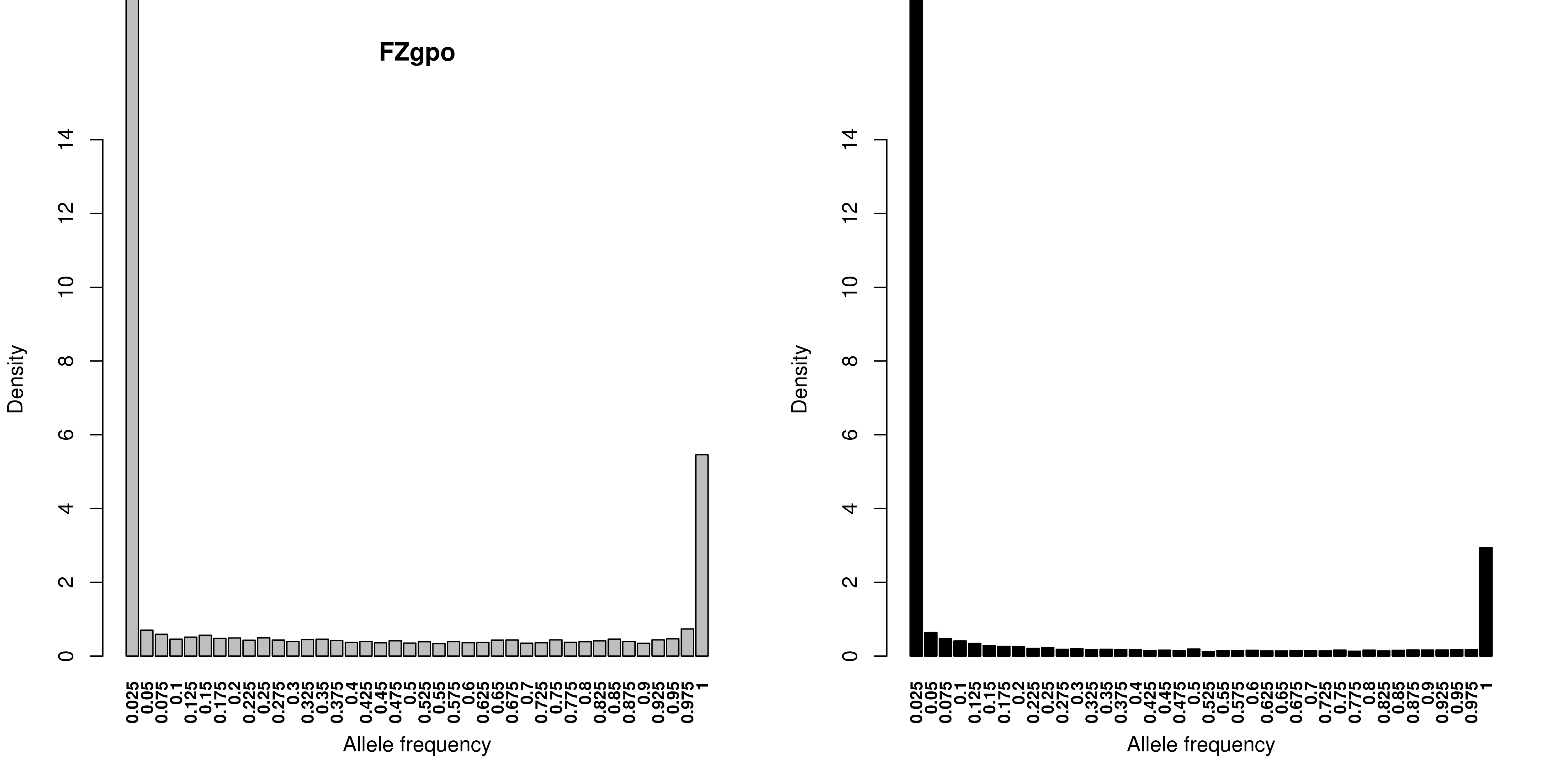

Supplement: Supplementary file 1 — Zip file containing allele frequency spectrum figures of each population. (ZIP 11230 kb) [file 12864_2017_4416_MOESM1_ESM.zip › additional_1 - Copy/AFS_array_WGS_FZgpo.tif]

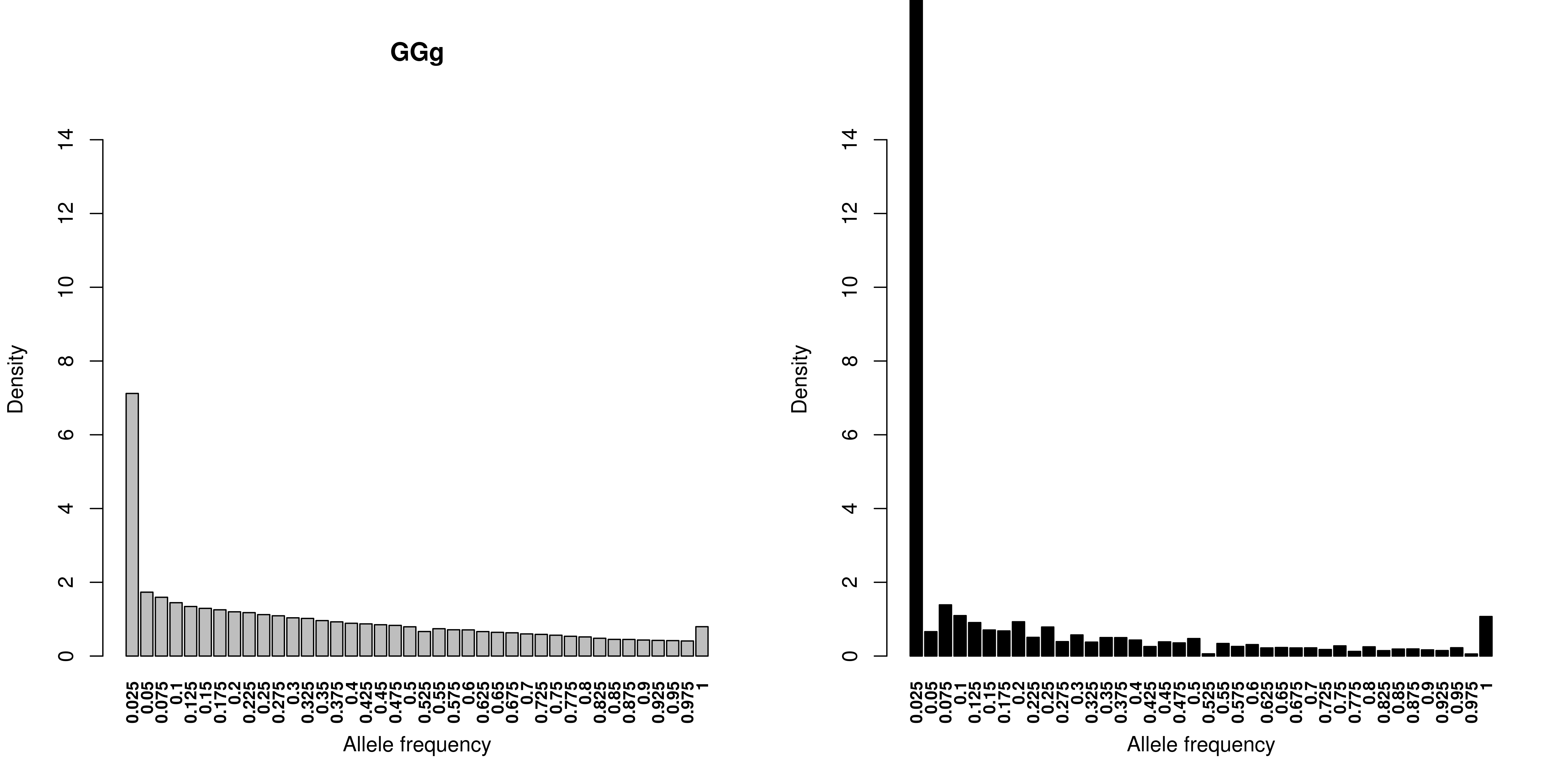

Supplement: Supplementary file 1 — Zip file containing allele frequency spectrum figures of each population. (ZIP 11230 kb) [file 12864_2017_4416_MOESM1_ESM.zip › additional_1 - Copy/AFS_array_WGS_GGg.tif]

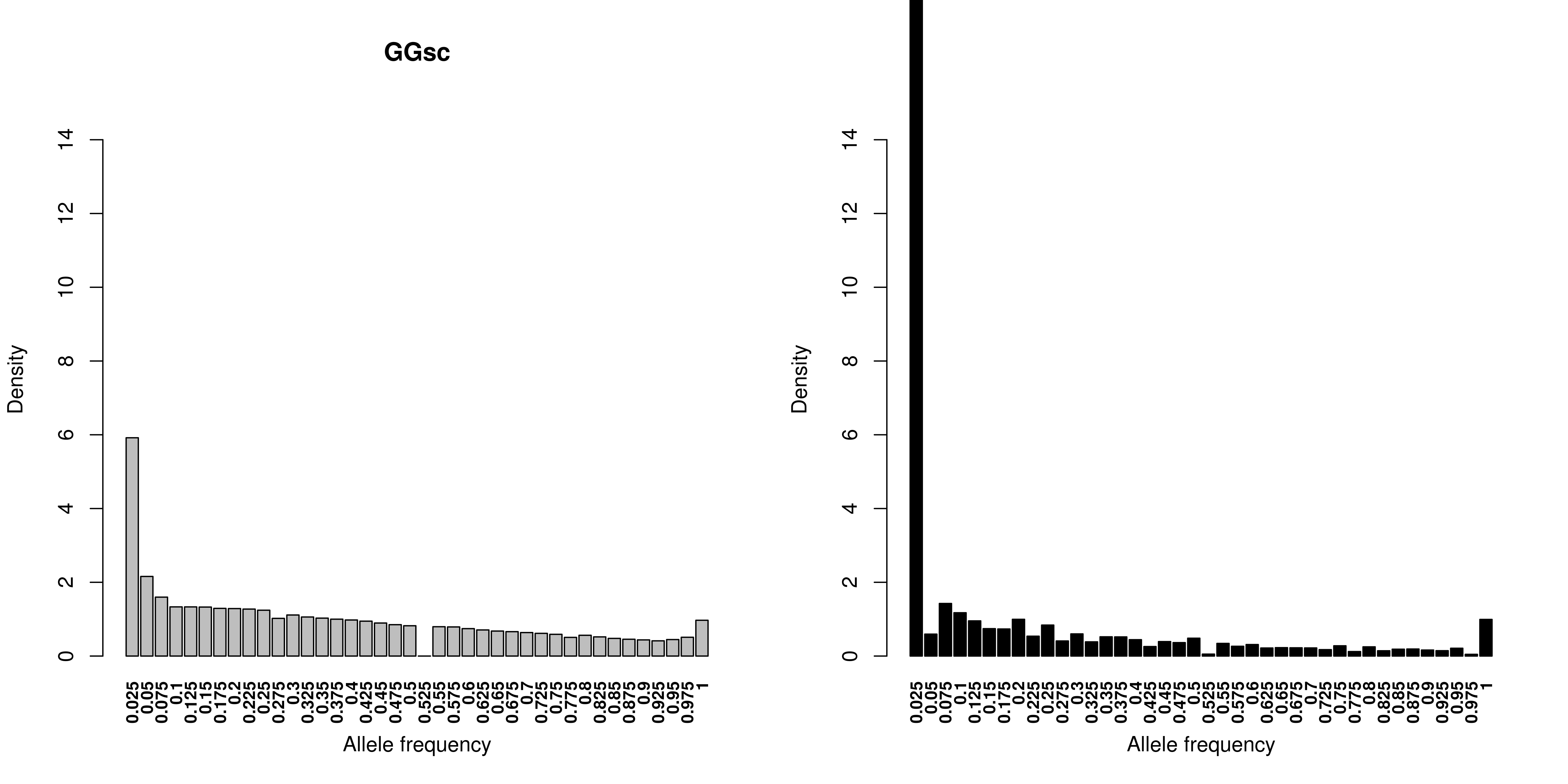

Supplement: Supplementary file 1 — Zip file containing allele frequency spectrum figures of each population. (ZIP 11230 kb) [file 12864_2017_4416_MOESM1_ESM.zip › additional_1 - Copy/AFS_array_WGS_GGsc.tif]

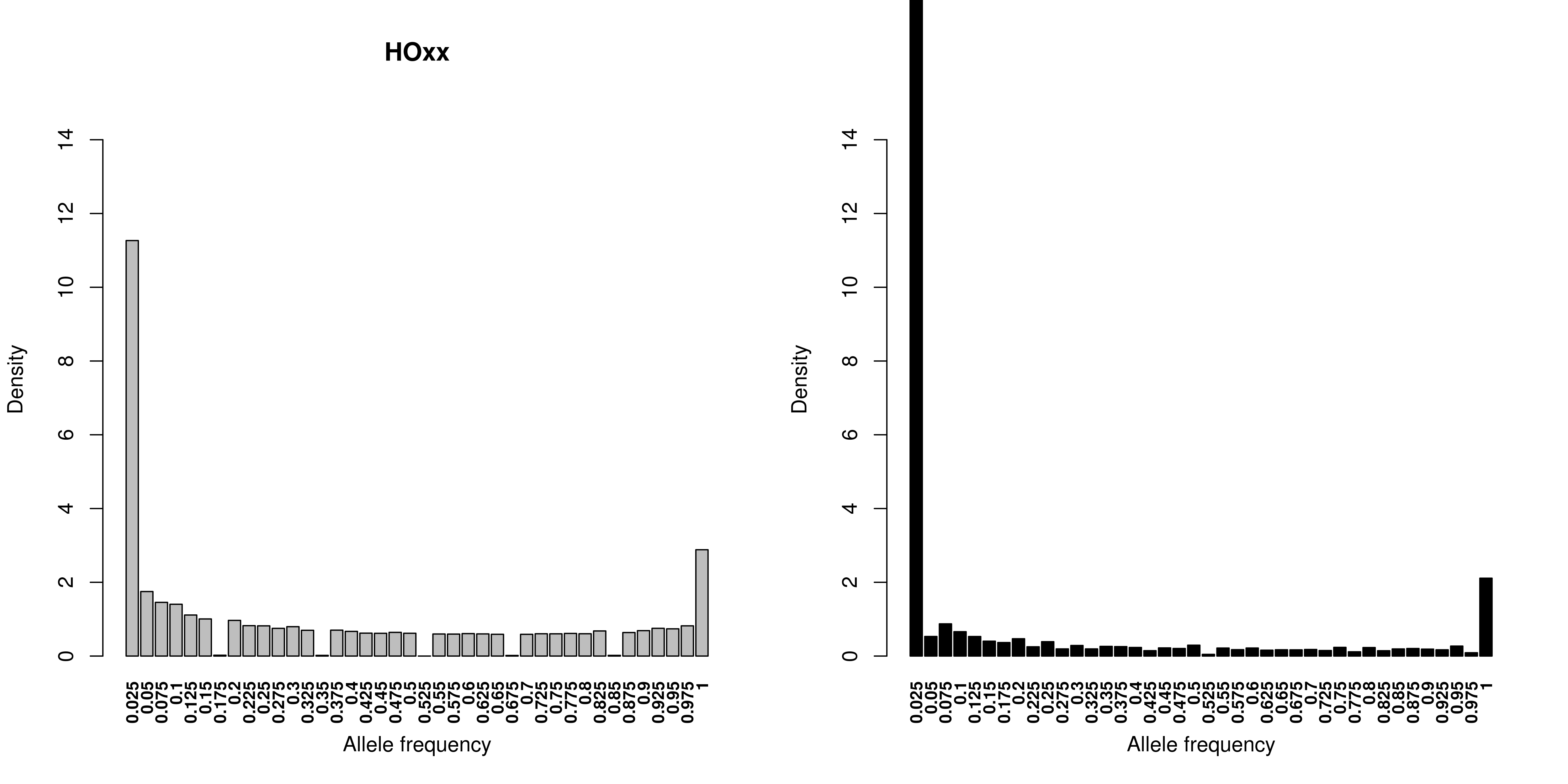

Supplement: Supplementary file 1 — Zip file containing allele frequency spectrum figures of each population. (ZIP 11230 kb) [file 12864_2017_4416_MOESM1_ESM.zip › additional_1 - Copy/AFS_array_WGS_HOxx.tif]

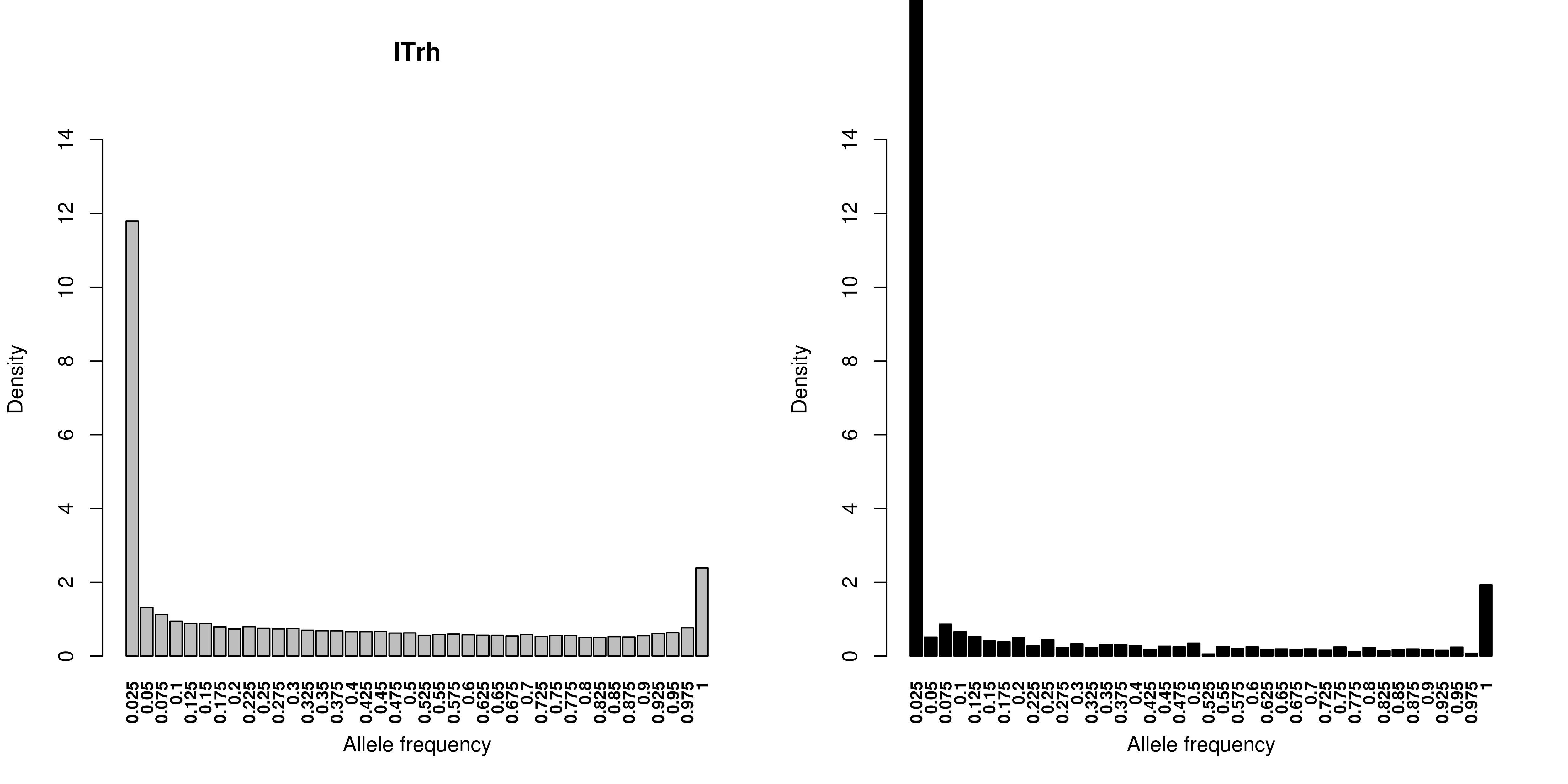

Supplement: Supplementary file 1 — Zip file containing allele frequency spectrum figures of each population. (ZIP 11230 kb) [file 12864_2017_4416_MOESM1_ESM.zip › additional_1 - Copy/AFS_array_WGS_ITrh.tif]

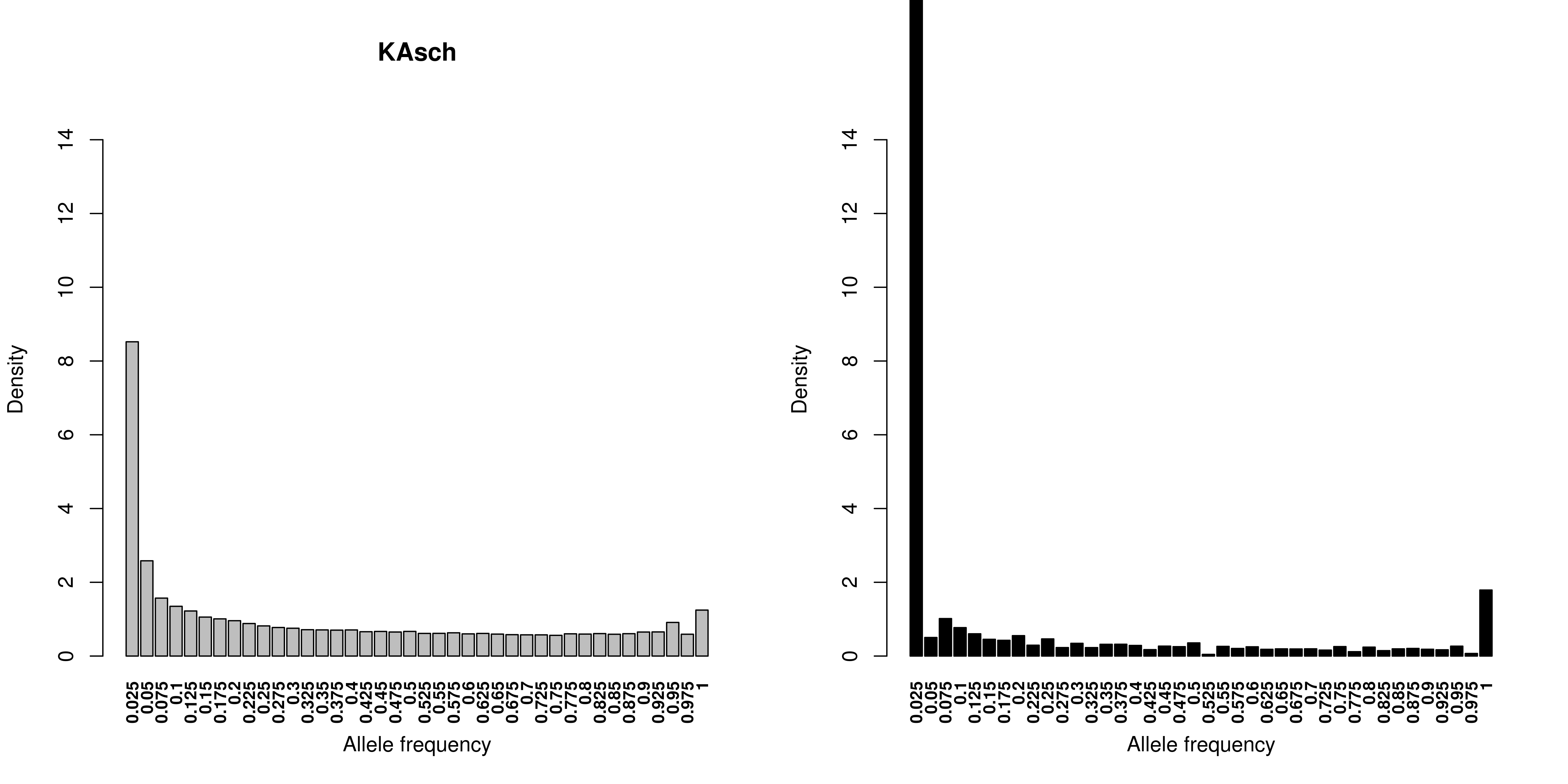

Supplement: Supplementary file 1 — Zip file containing allele frequency spectrum figures of each population. (ZIP 11230 kb) [file 12864_2017_4416_MOESM1_ESM.zip › additional_1 - Copy/AFS_array_WGS_KAsch.tif]

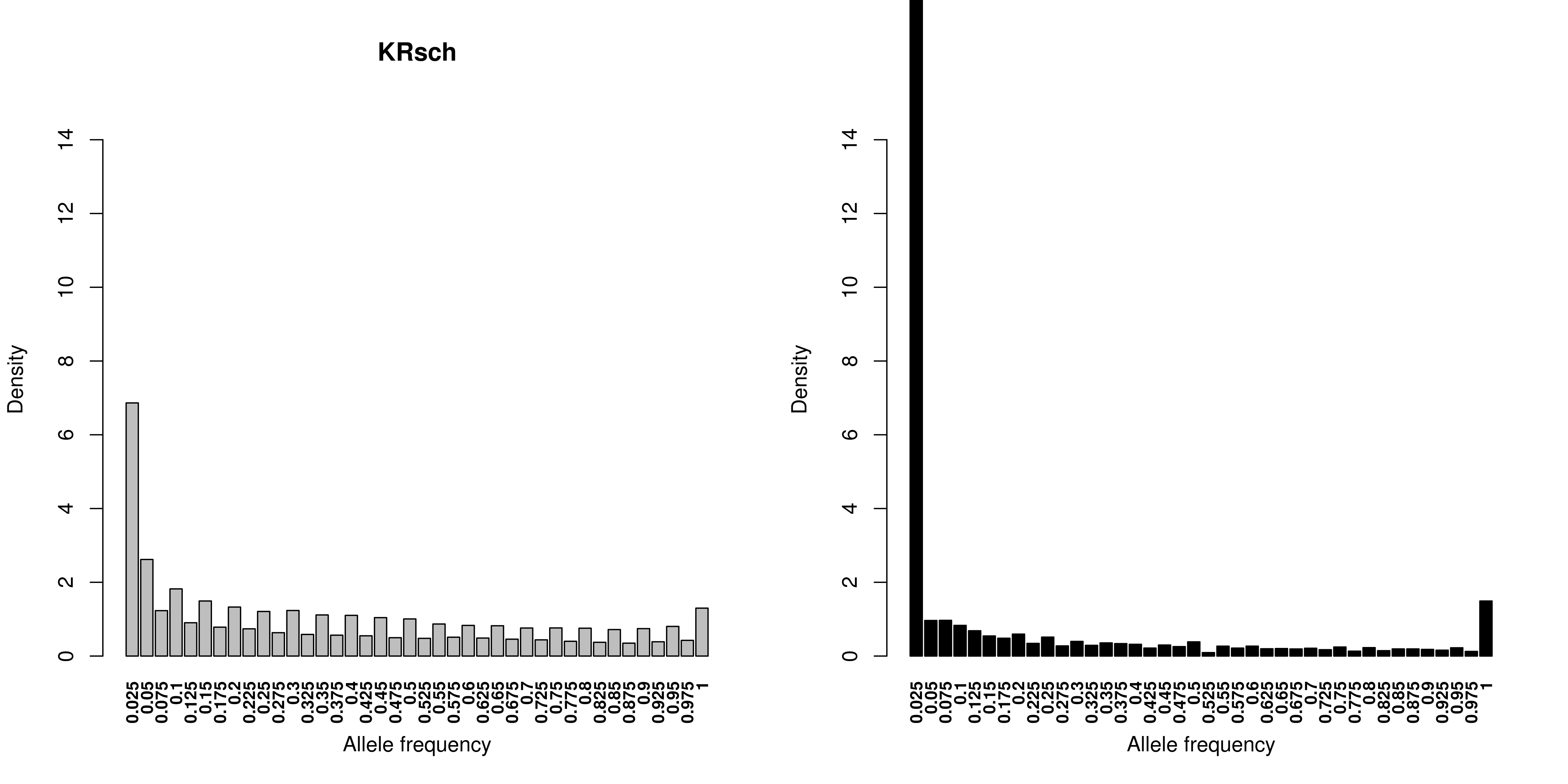

Supplement: Supplementary file 1 — Zip file containing allele frequency spectrum figures of each population. (ZIP 11230 kb) [file 12864_2017_4416_MOESM1_ESM.zip › additional_1 - Copy/AFS_array_WGS_KRsch.tif]

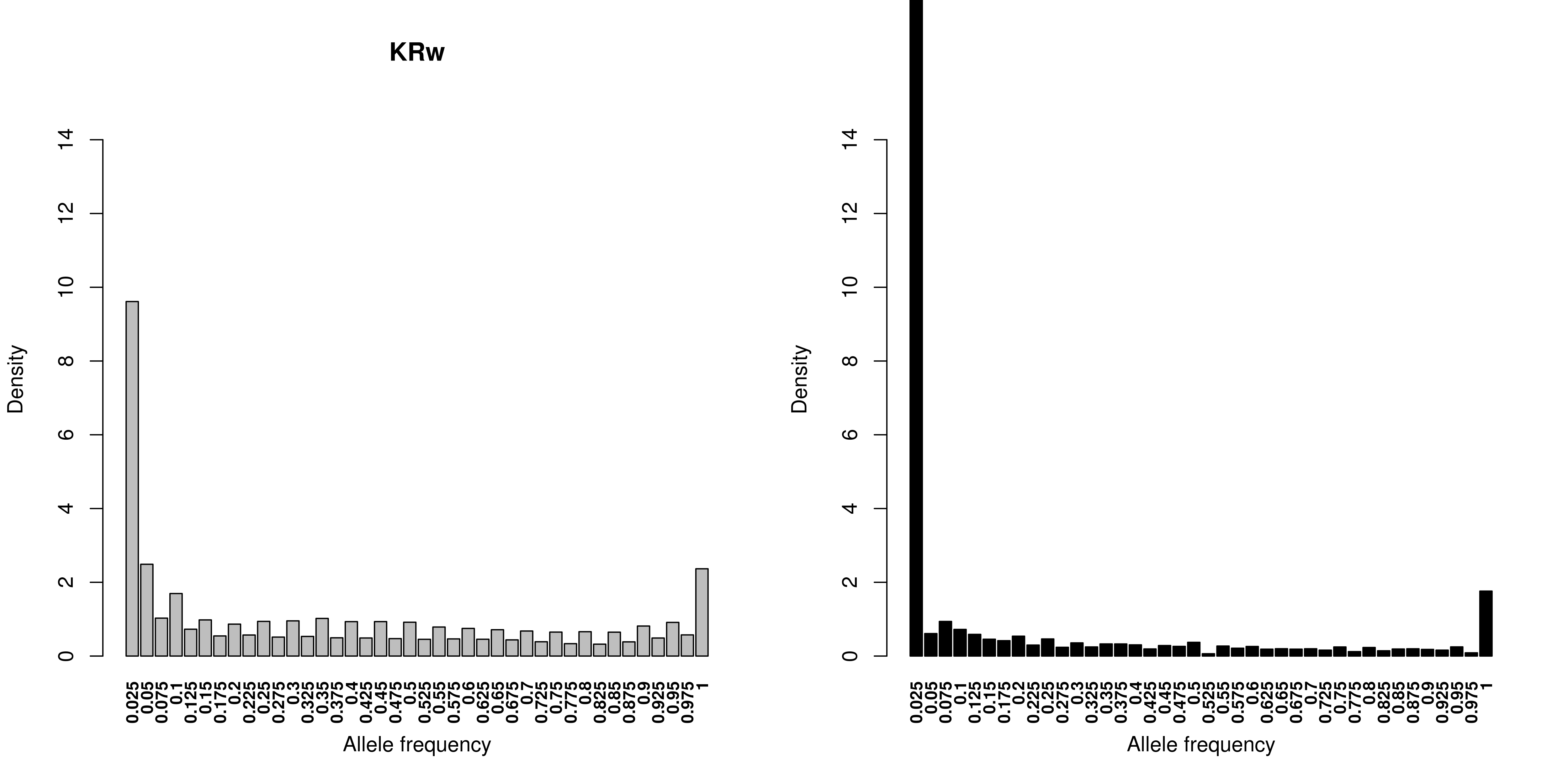

Supplement: Supplementary file 1 — Zip file containing allele frequency spectrum figures of each population. (ZIP 11230 kb) [file 12864_2017_4416_MOESM1_ESM.zip › additional_1 - Copy/AFS_array_WGS_KRw.tif]

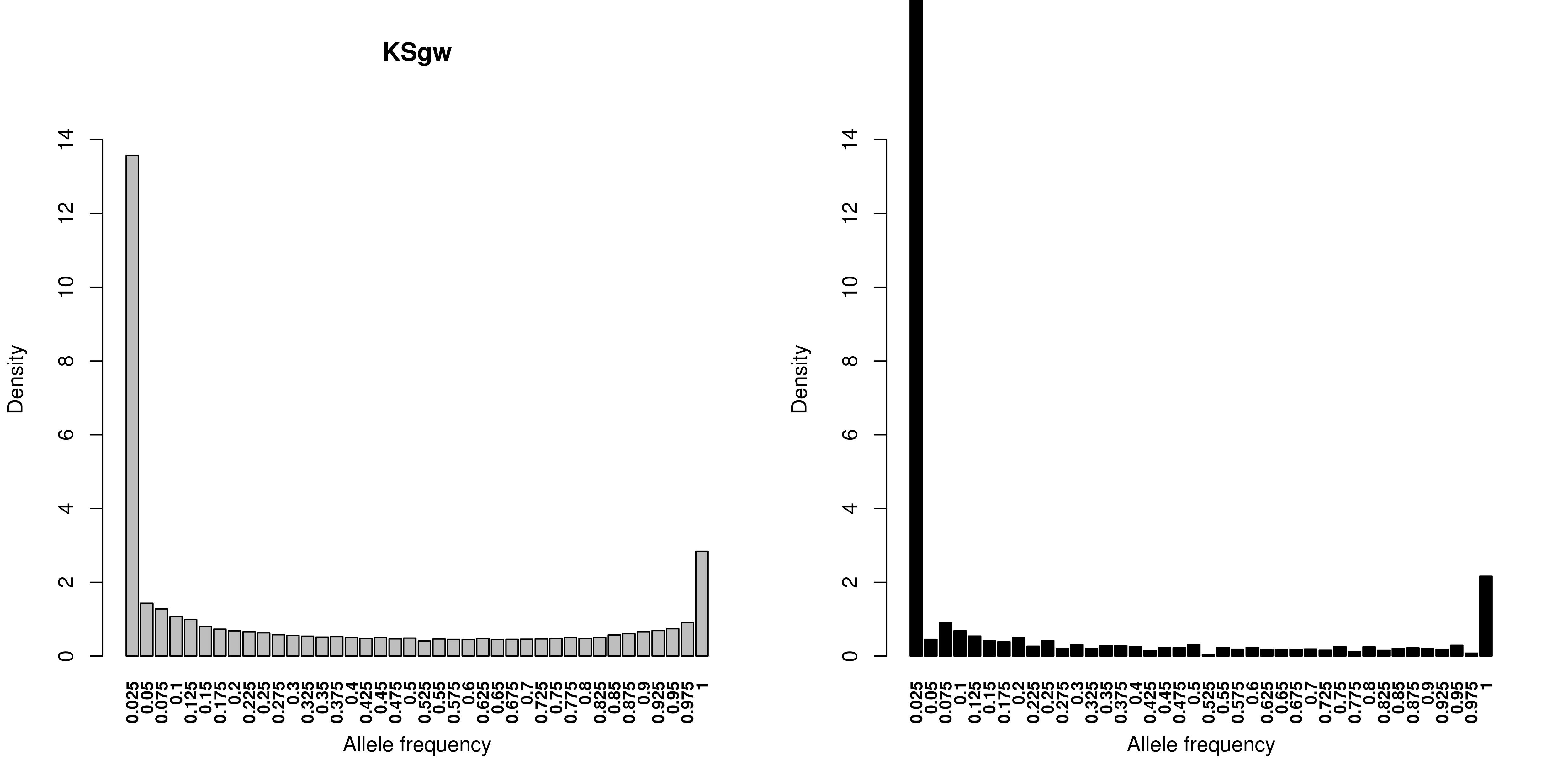

Supplement: Supplementary file 1 — Zip file containing allele frequency spectrum figures of each population. (ZIP 11230 kb) [file 12864_2017_4416_MOESM1_ESM.zip › additional_1 - Copy/AFS_array_WGS_KSgw.tif]

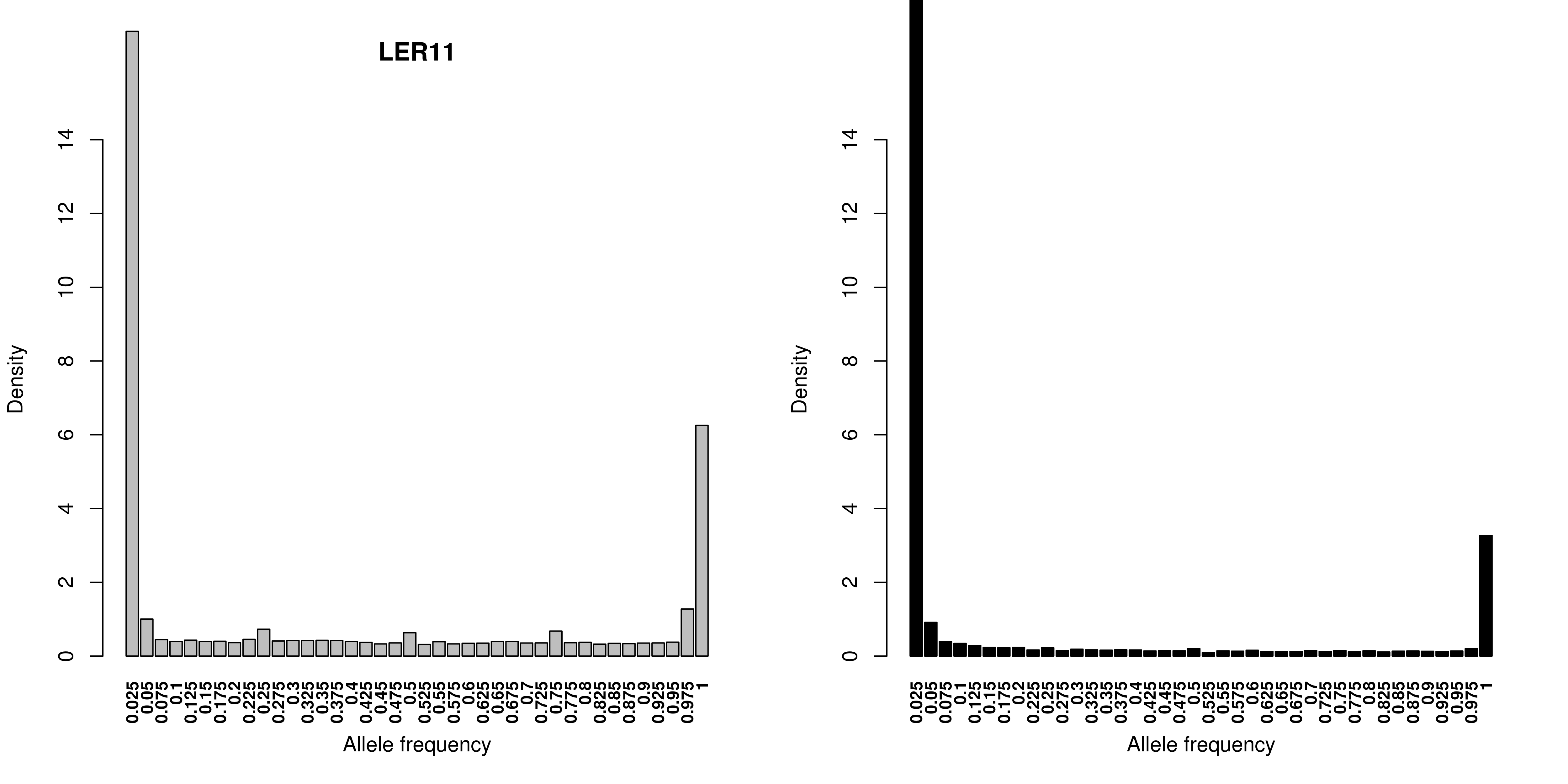

Supplement: Supplementary file 1 — Zip file containing allele frequency spectrum figures of each population. (ZIP 11230 kb) [file 12864_2017_4416_MOESM1_ESM.zip › additional_1 - Copy/AFS_array_WGS_LER11.tif]

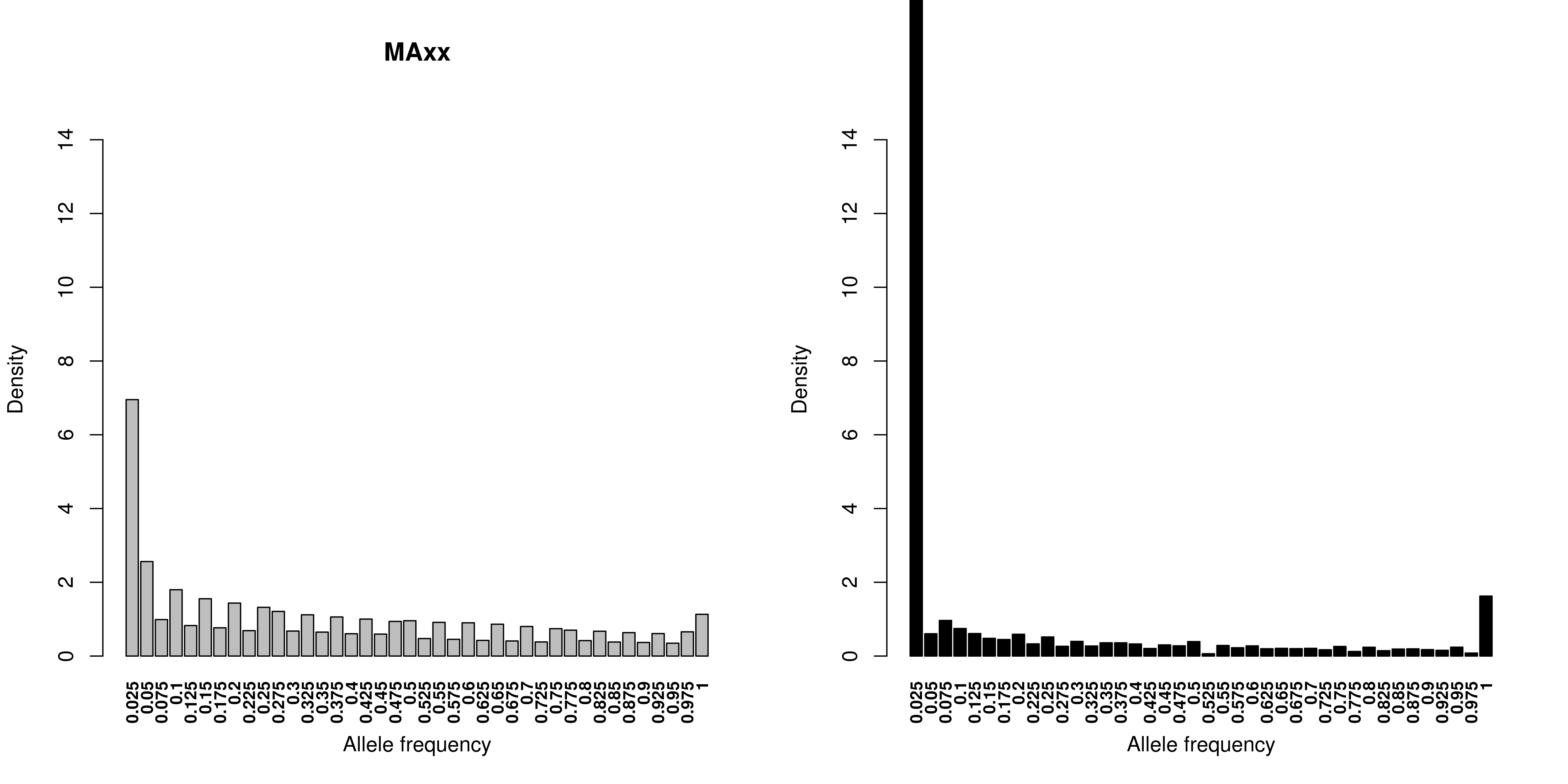

Supplement: Supplementary file 1 — Zip file containing allele frequency spectrum figures of each population. (ZIP 11230 kb) [file 12864_2017_4416_MOESM1_ESM.zip › additional_1 - Copy/AFS_array_WGS_MAxx.tif]

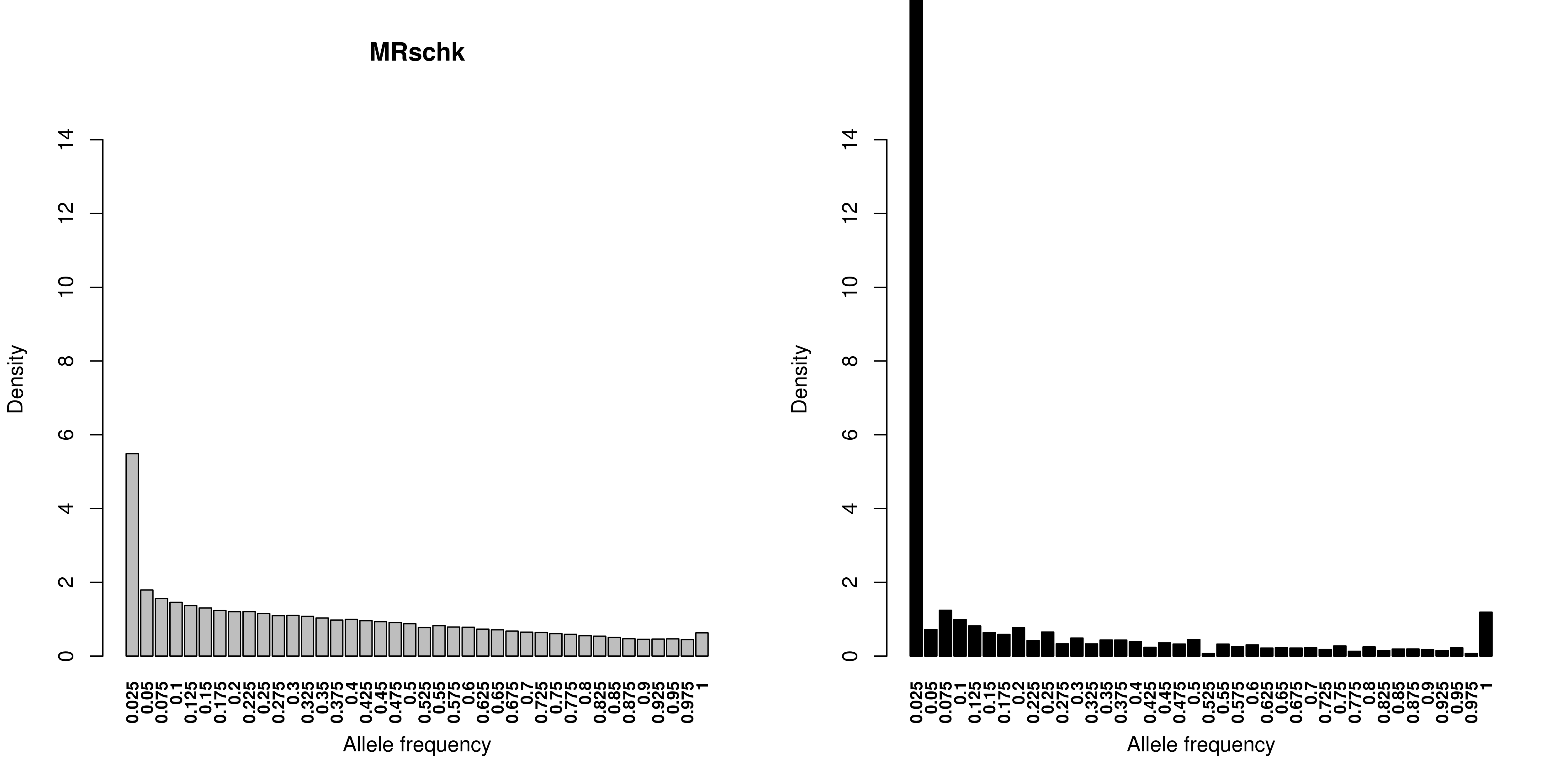

Supplement: Supplementary file 1 — Zip file containing allele frequency spectrum figures of each population. (ZIP 11230 kb) [file 12864_2017_4416_MOESM1_ESM.zip › additional_1 - Copy/AFS_array_WGS_MRschk.tif]

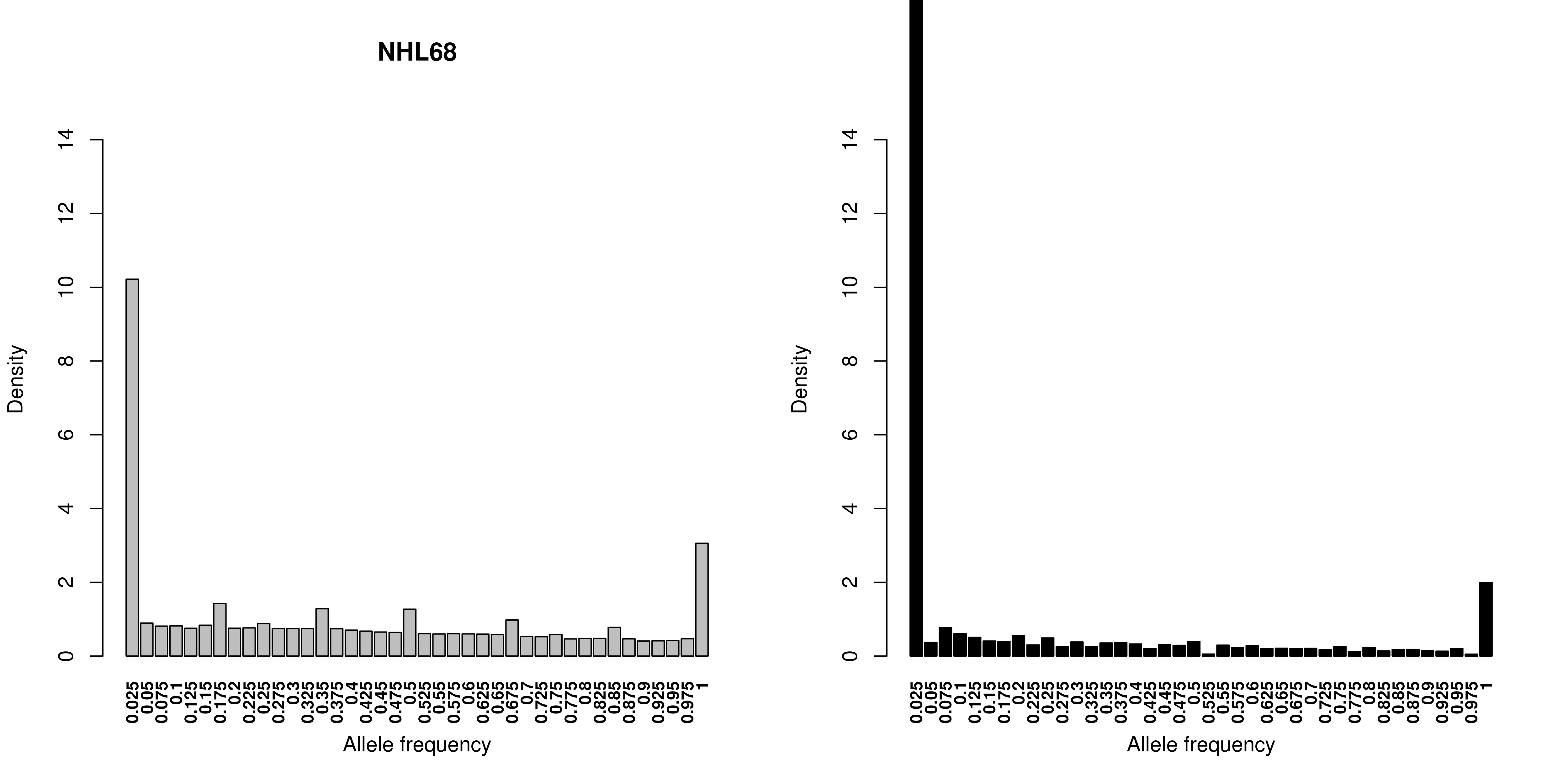

Supplement: Supplementary file 1 — Zip file containing allele frequency spectrum figures of each population. (ZIP 11230 kb) [file 12864_2017_4416_MOESM1_ESM.zip › additional_1 - Copy/AFS_array_WGS_NHL68.tif]

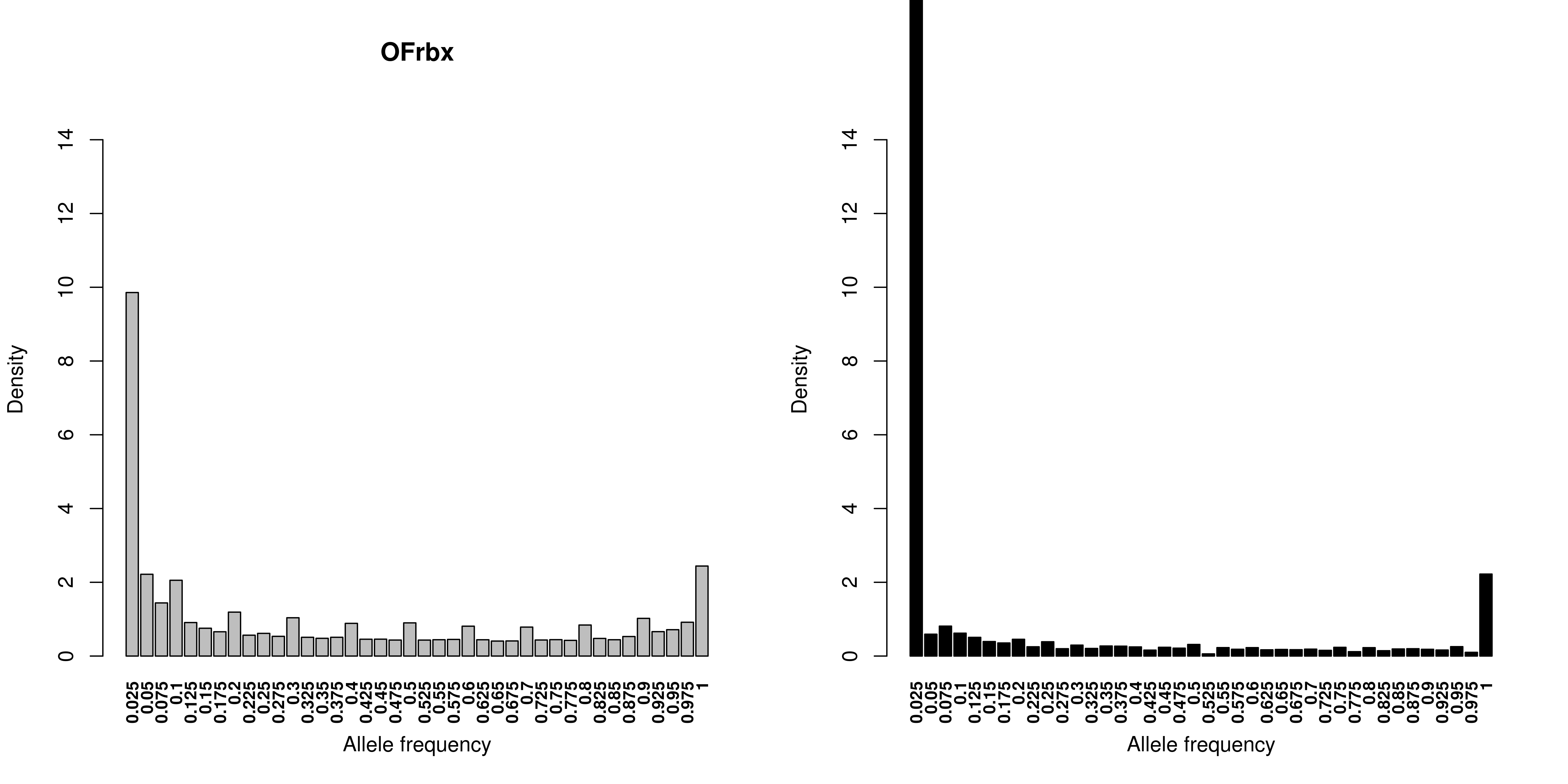

Supplement: Supplementary file 1 — Zip file containing allele frequency spectrum figures of each population. (ZIP 11230 kb) [file 12864_2017_4416_MOESM1_ESM.zip › additional_1 - Copy/AFS_array_WGS_OFrbx.tif]

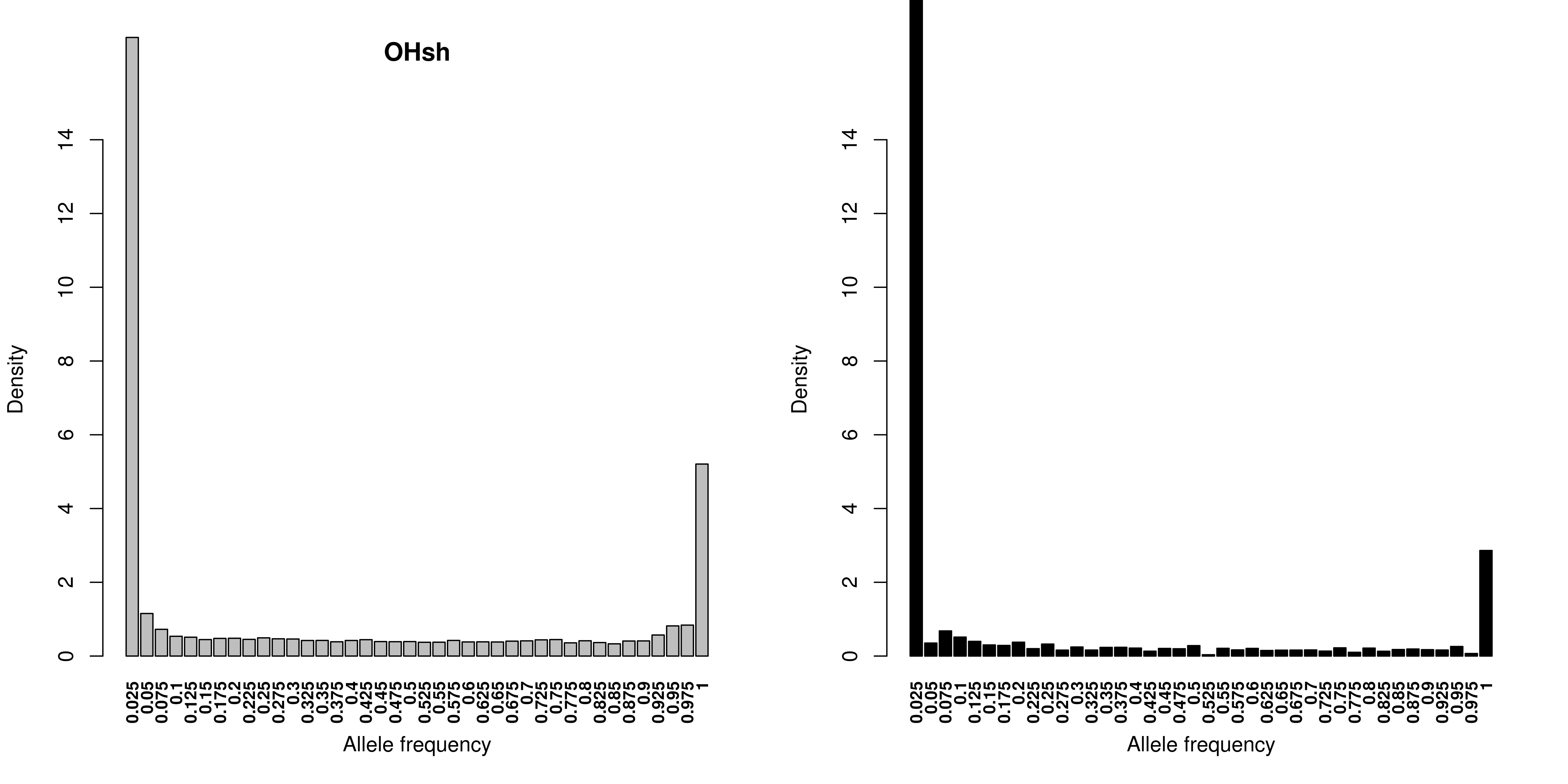

Supplement: Supplementary file 1 — Zip file containing allele frequency spectrum figures of each population. (ZIP 11230 kb) [file 12864_2017_4416_MOESM1_ESM.zip › additional_1 - Copy/AFS_array_WGS_OHsh.tif]

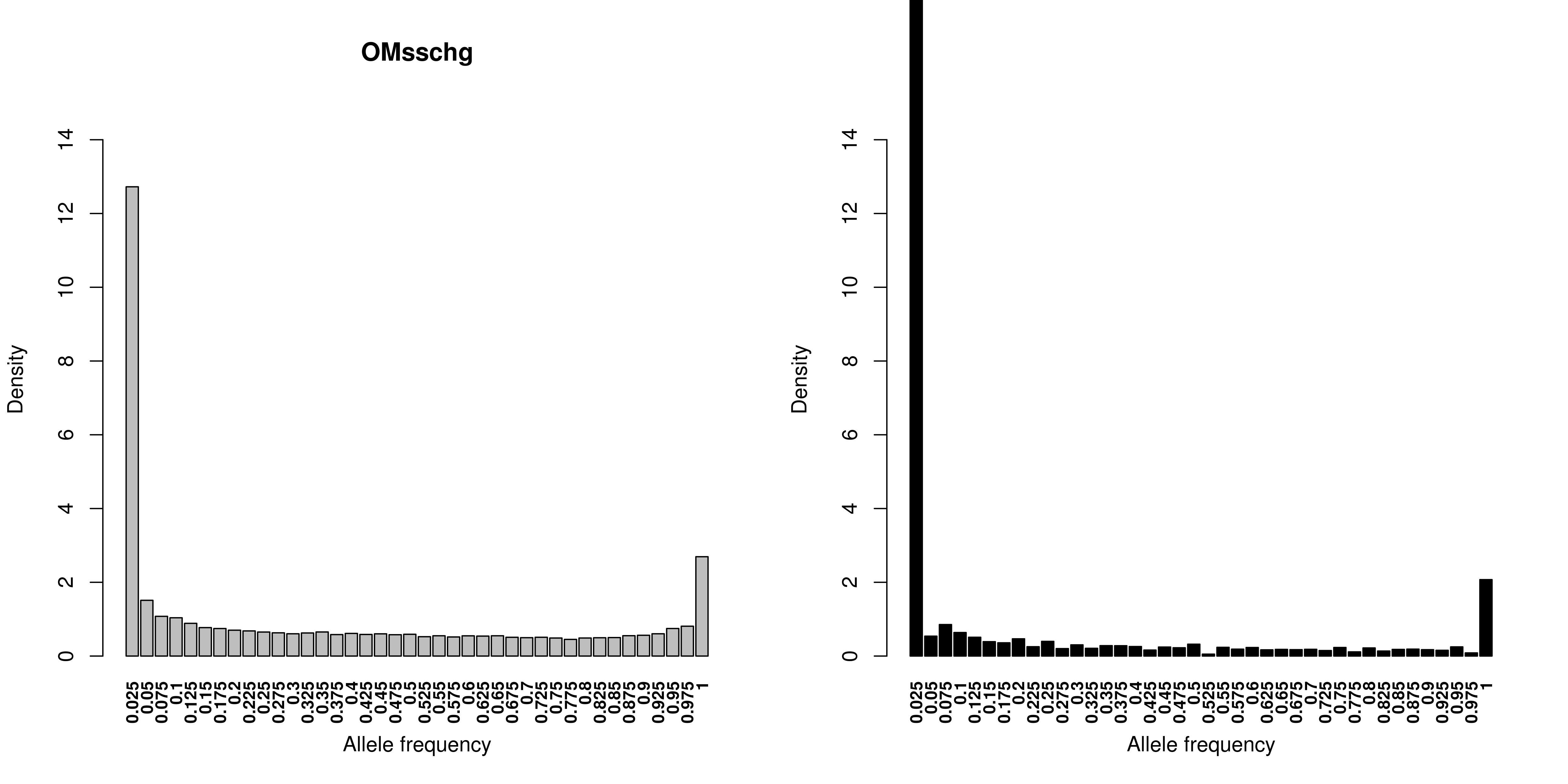

Supplement: Supplementary file 1 — Zip file containing allele frequency spectrum figures of each population. (ZIP 11230 kb) [file 12864_2017_4416_MOESM1_ESM.zip › additional_1 - Copy/AFS_array_WGS_OMsschg.tif]

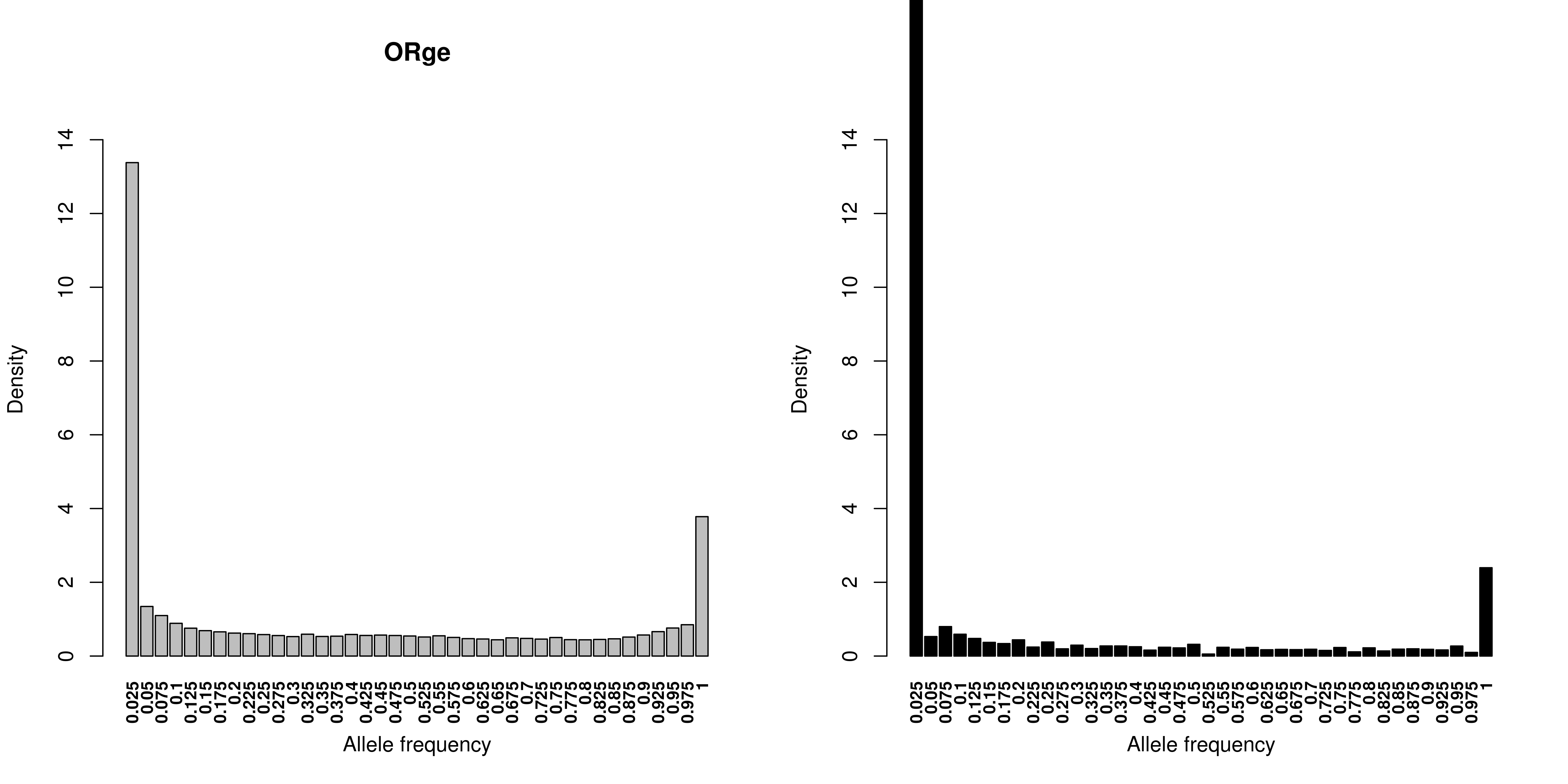

Supplement: Supplementary file 1 — Zip file containing allele frequency spectrum figures of each population. (ZIP 11230 kb) [file 12864_2017_4416_MOESM1_ESM.zip › additional_1 - Copy/AFS_array_WGS_ORge.tif]

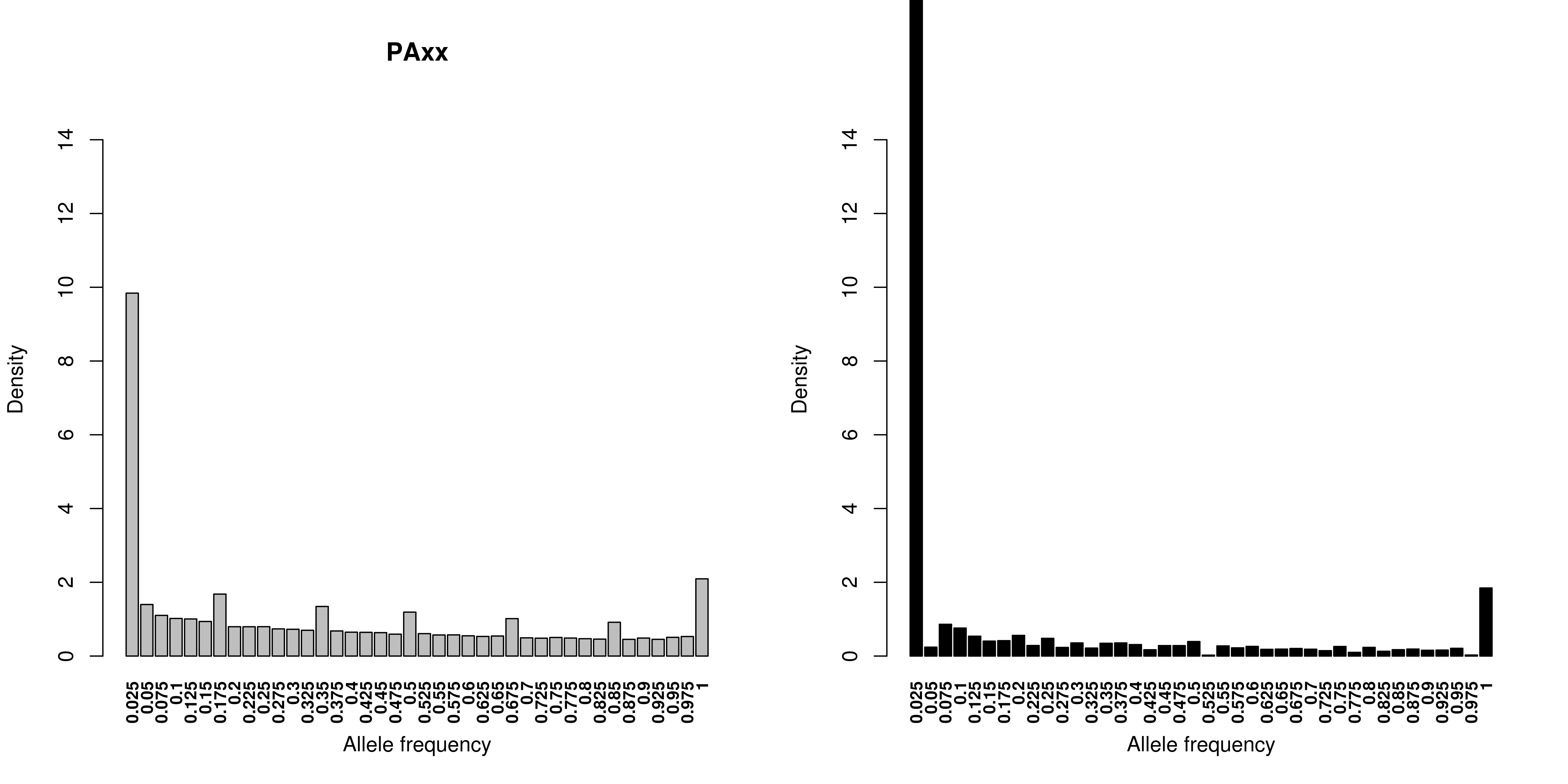

Supplement: Supplementary file 1 — Zip file containing allele frequency spectrum figures of each population. (ZIP 11230 kb) [file 12864_2017_4416_MOESM1_ESM.zip › additional_1 - Copy/AFS_array_WGS_PAxx.tif]

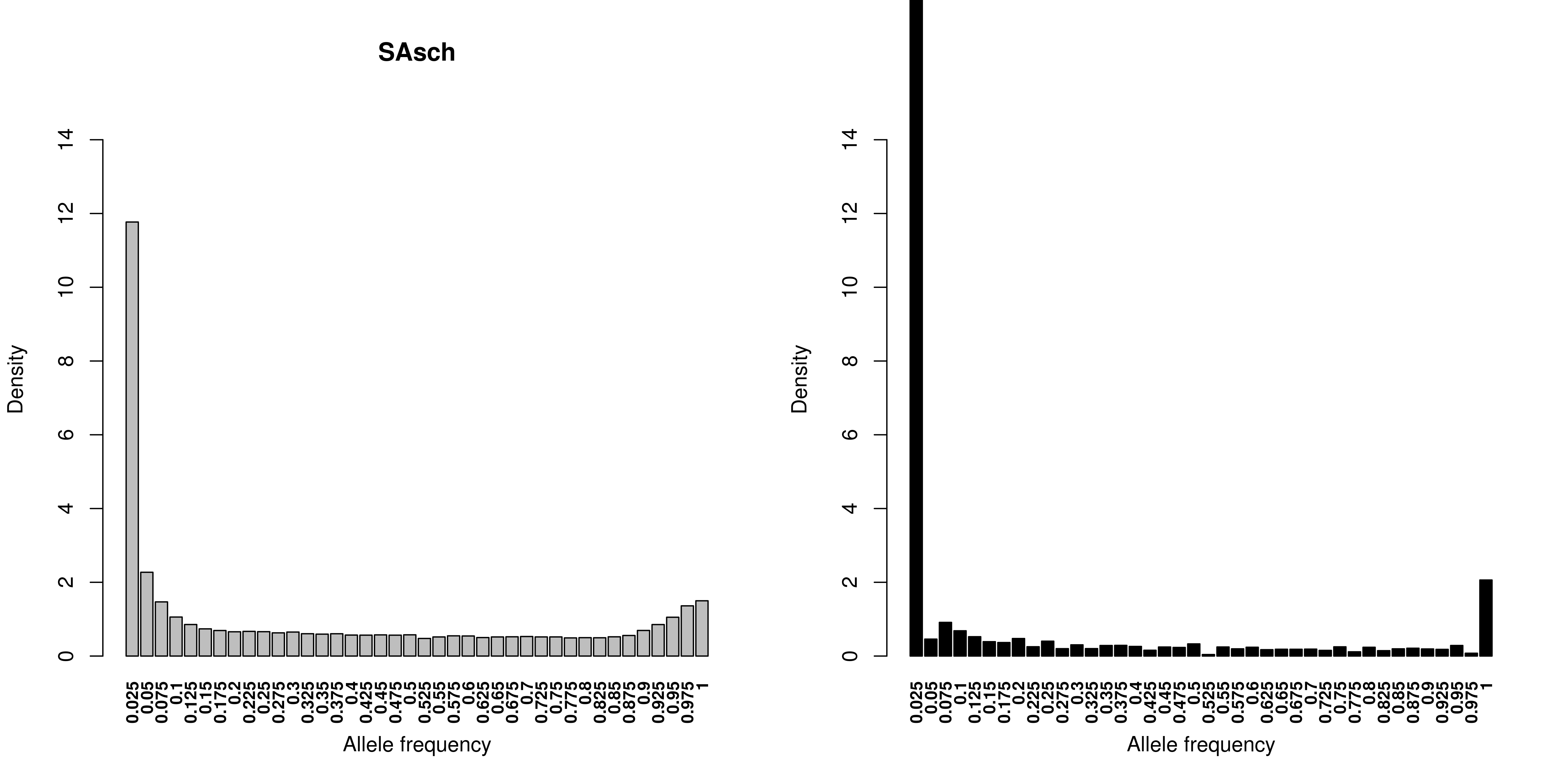

Supplement: Supplementary file 1 — Zip file containing allele frequency spectrum figures of each population. (ZIP 11230 kb) [file 12864_2017_4416_MOESM1_ESM.zip › additional_1 - Copy/AFS_array_WGS_SAsch.tif]

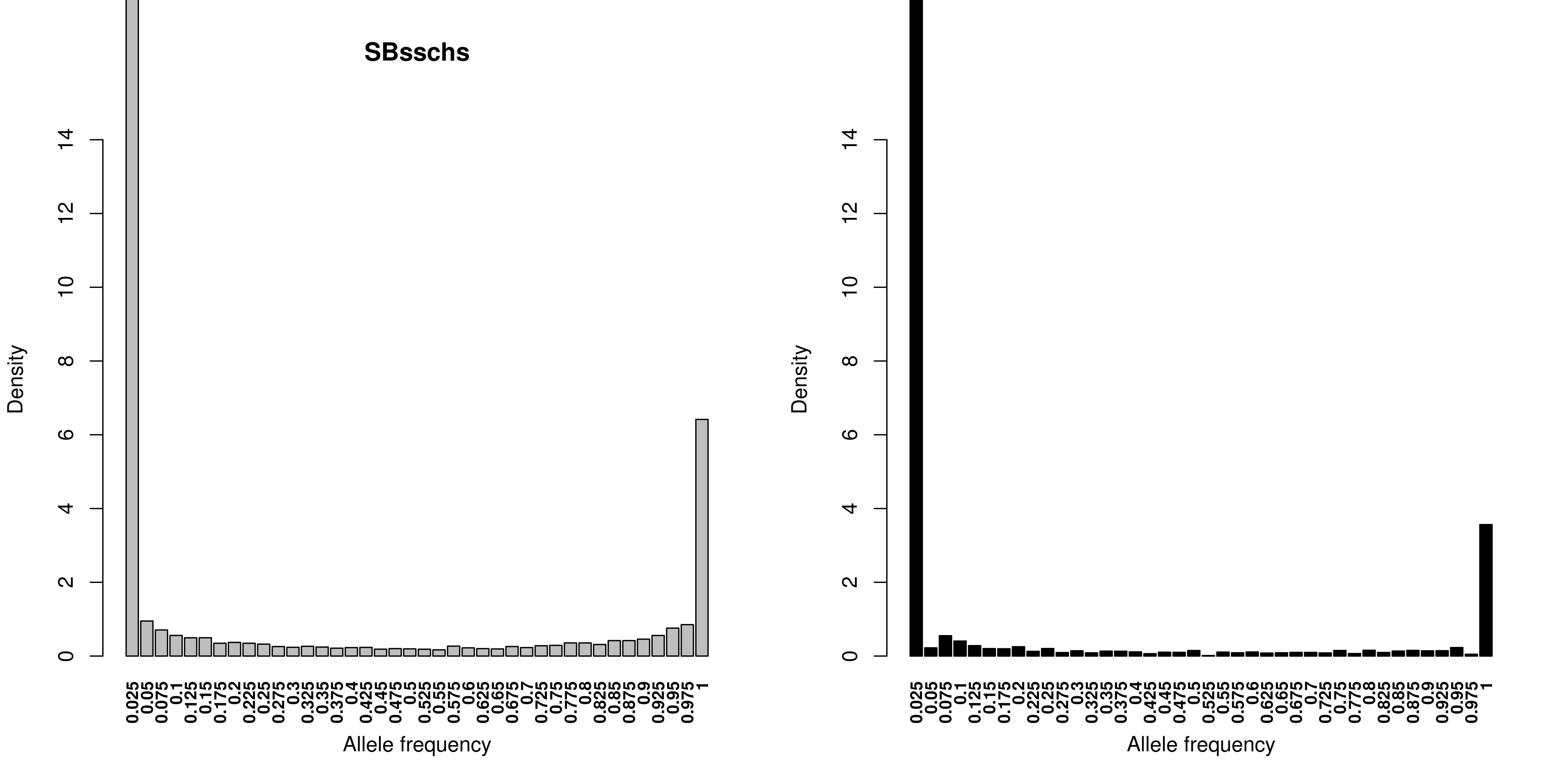

Supplement: Supplementary file 1 — Zip file containing allele frequency spectrum figures of each population. (ZIP 11230 kb) [file 12864_2017_4416_MOESM1_ESM.zip › additional_1 - Copy/AFS_array_WGS_SBsschs.tif]

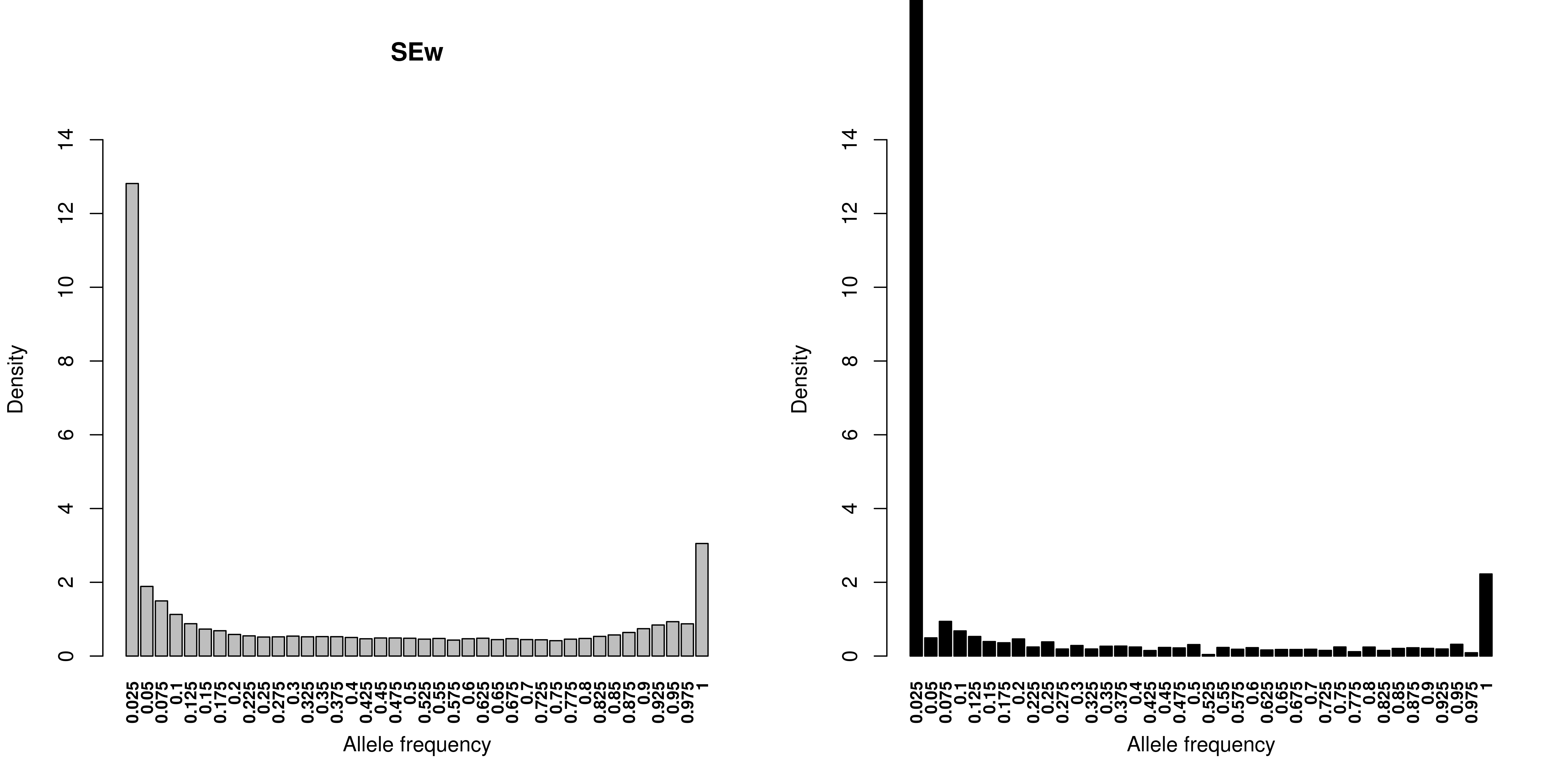

Supplement: Supplementary file 1 — Zip file containing allele frequency spectrum figures of each population. (ZIP 11230 kb) [file 12864_2017_4416_MOESM1_ESM.zip › additional_1 - Copy/AFS_array_WGS_SEw.tif]

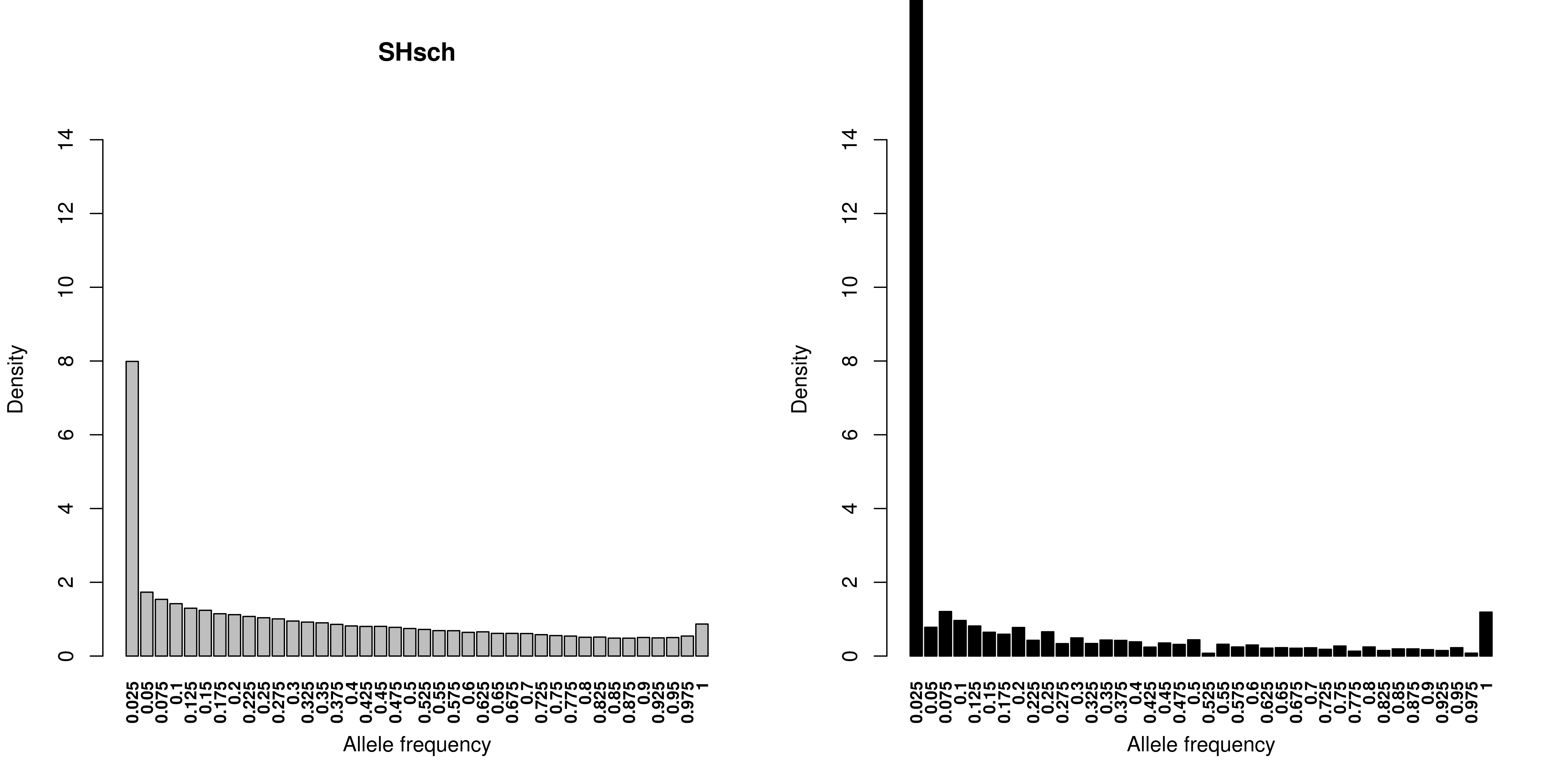

Supplement: Supplementary file 1 — Zip file containing allele frequency spectrum figures of each population. (ZIP 11230 kb) [file 12864_2017_4416_MOESM1_ESM.zip › additional_1 - Copy/AFS_array_WGS_SHsch.tif]

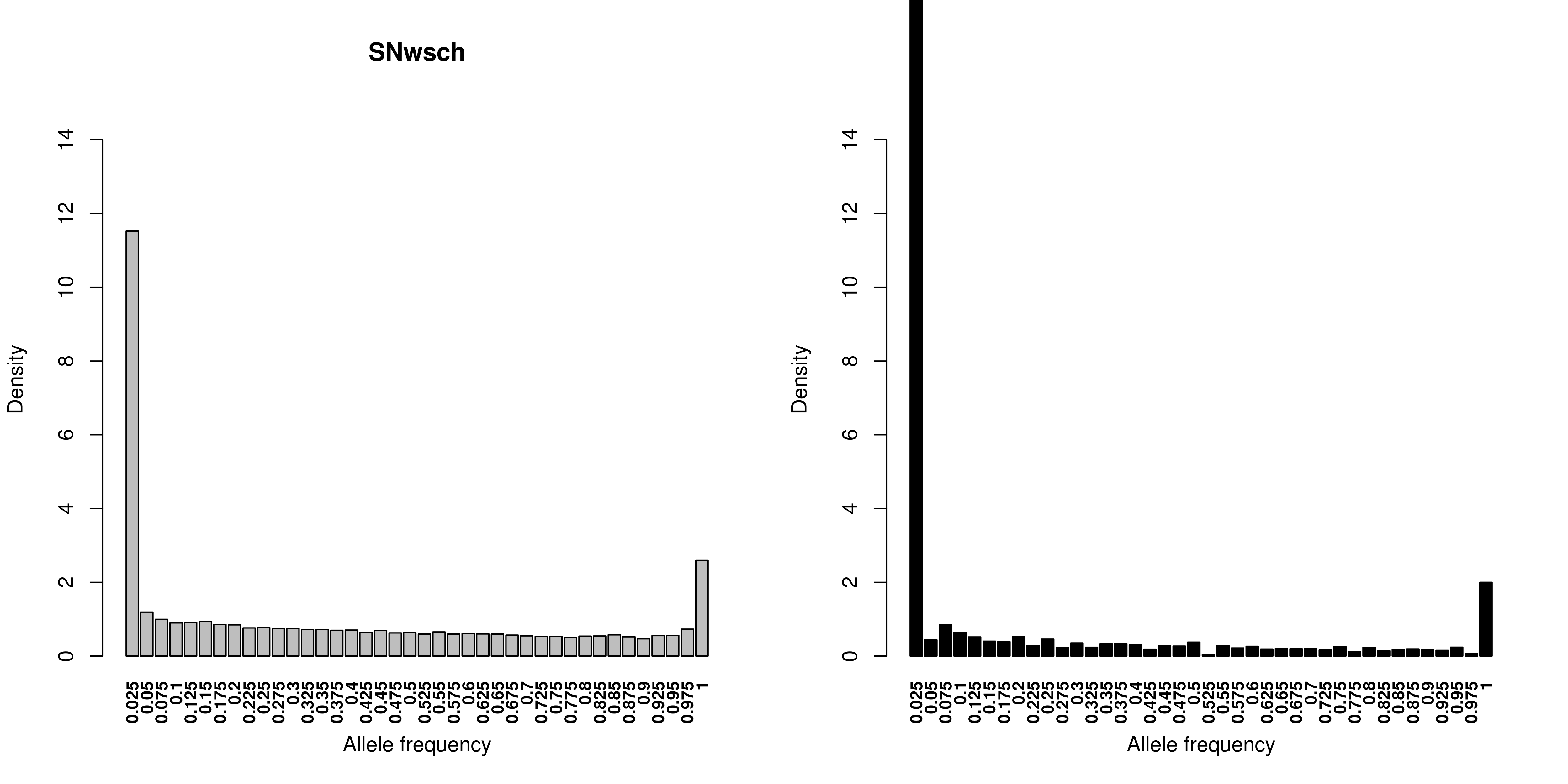

Supplement: Supplementary file 1 — Zip file containing allele frequency spectrum figures of each population. (ZIP 11230 kb) [file 12864_2017_4416_MOESM1_ESM.zip › additional_1 - Copy/AFS_array_WGS_SNwsch.tif]

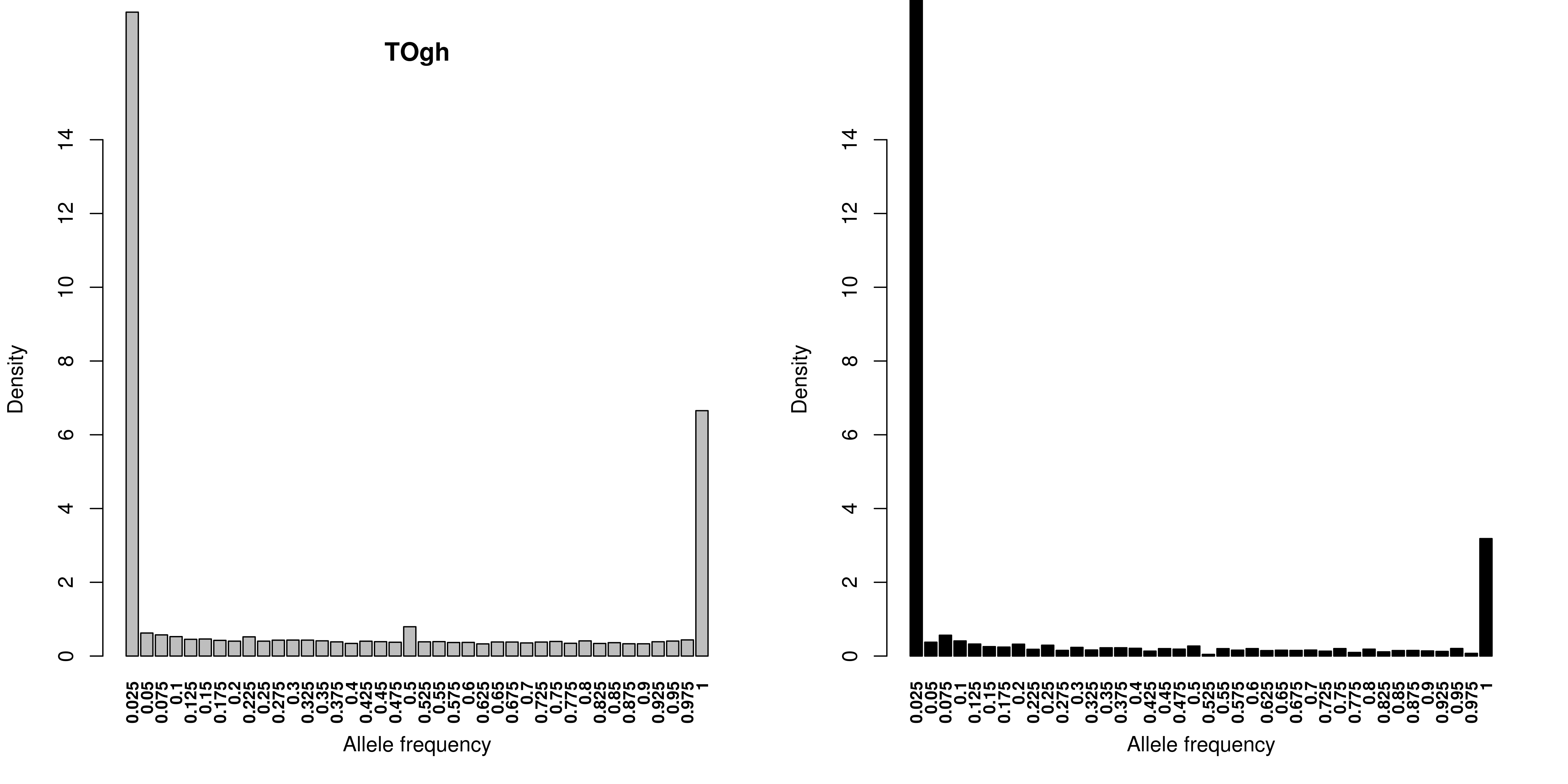

Supplement: Supplementary file 1 — Zip file containing allele frequency spectrum figures of each population. (ZIP 11230 kb) [file 12864_2017_4416_MOESM1_ESM.zip › additional_1 - Copy/AFS_array_WGS_TOgh.tif]

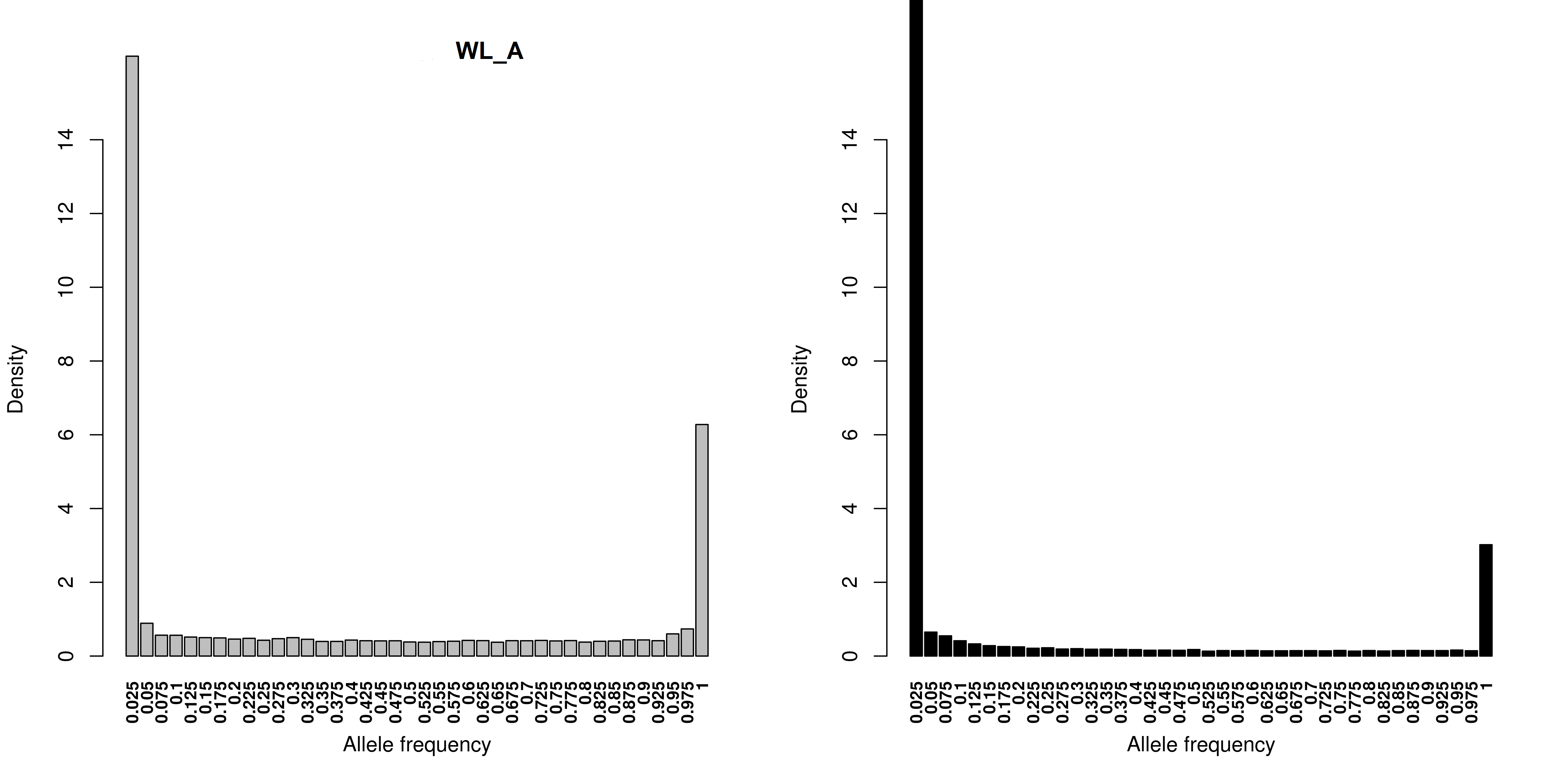

Supplement: Supplementary file 1 — Zip file containing allele frequency spectrum figures of each population. (ZIP 11230 kb) [file 12864_2017_4416_MOESM1_ESM.zip › additional_1 - Copy/AFS_array_WGS_WL_A.tif]

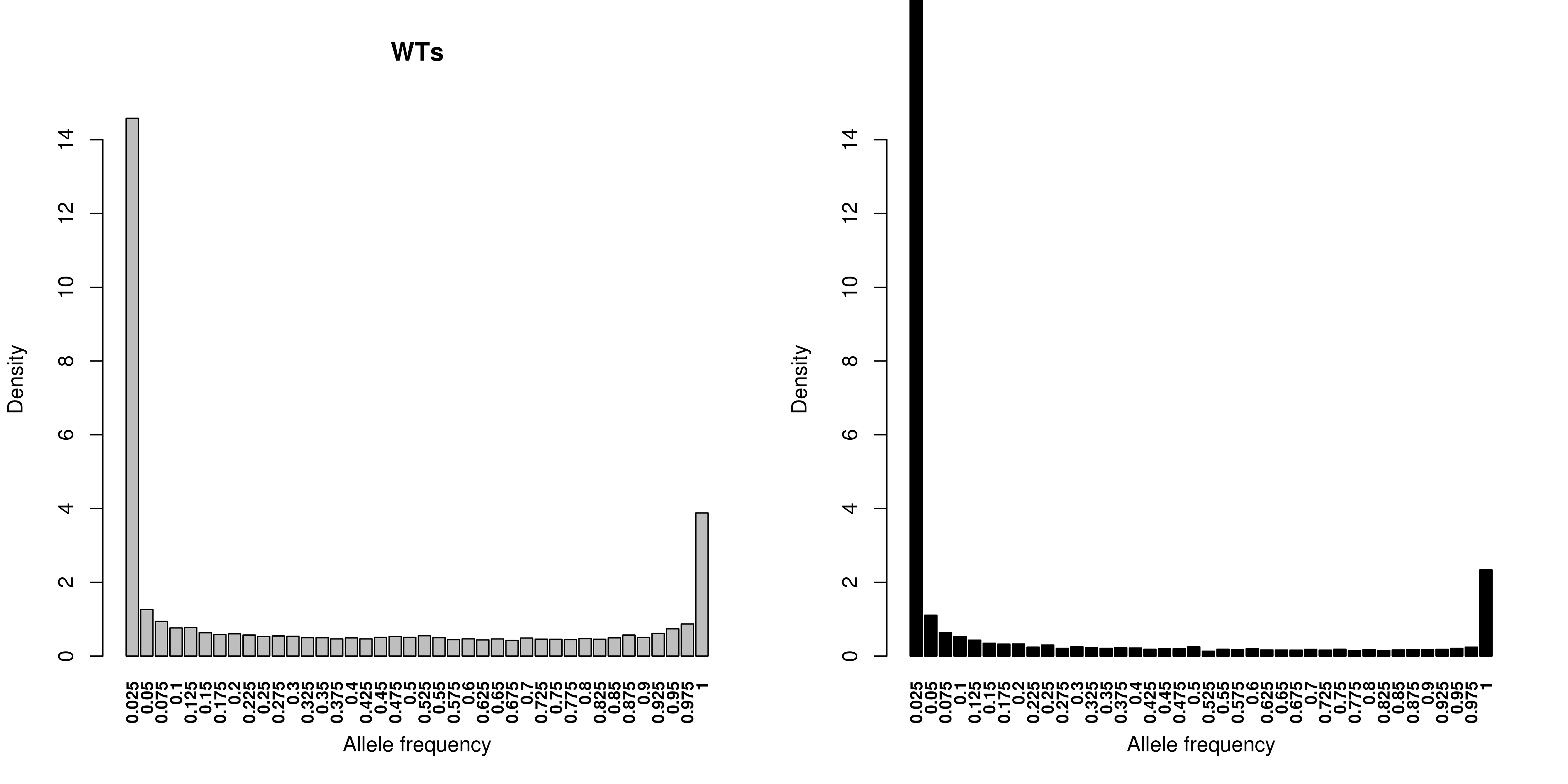

Supplement: Supplementary file 1 — Zip file containing allele frequency spectrum figures of each population. (ZIP 11230 kb) [file 12864_2017_4416_MOESM1_ESM.zip › additional_1 - Copy/AFS_array_WGS_WTs.tif]

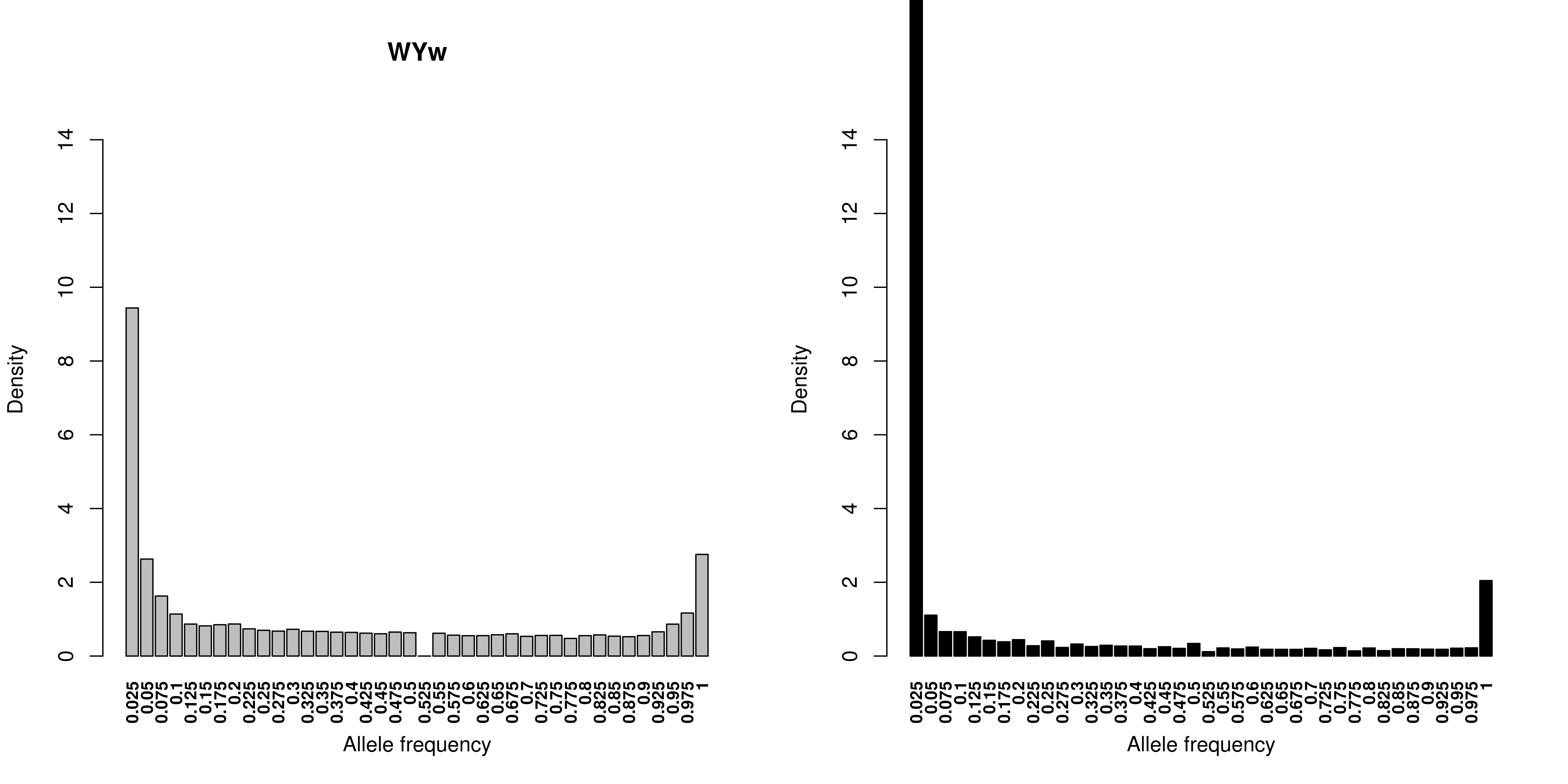

Supplement: Supplementary file 1 — Zip file containing allele frequency spectrum figures of each population. (ZIP 11230 kb) [file 12864_2017_4416_MOESM1_ESM.zip › additional_1 - Copy/AFS_array_WGS_WYw.tif]

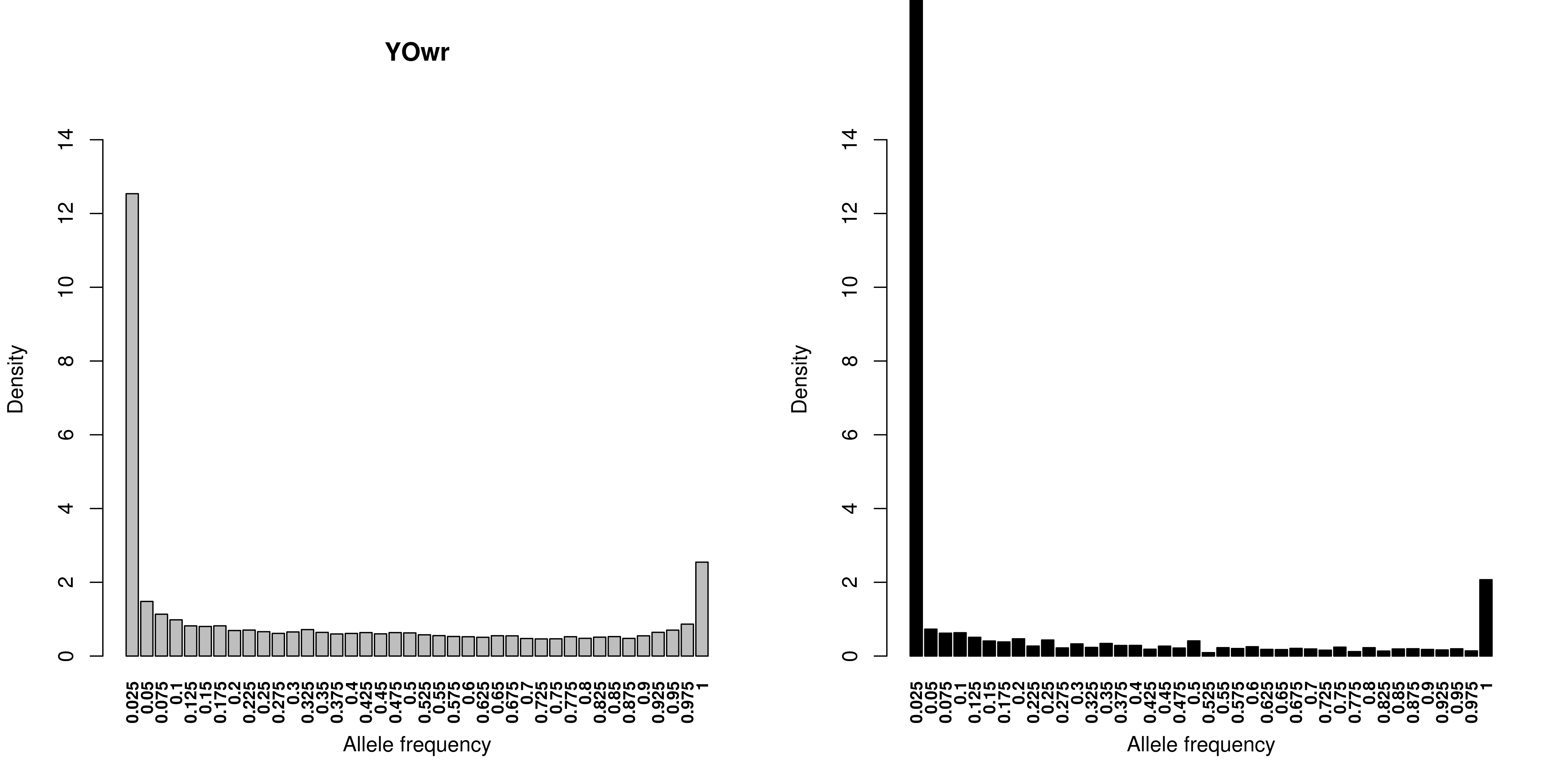

Supplement: Supplementary file 1 — Zip file containing allele frequency spectrum figures of each population. (ZIP 11230 kb) [file 12864_2017_4416_MOESM1_ESM.zip › additional_1 - Copy/AFS_array_WGS_YOwr.tif]

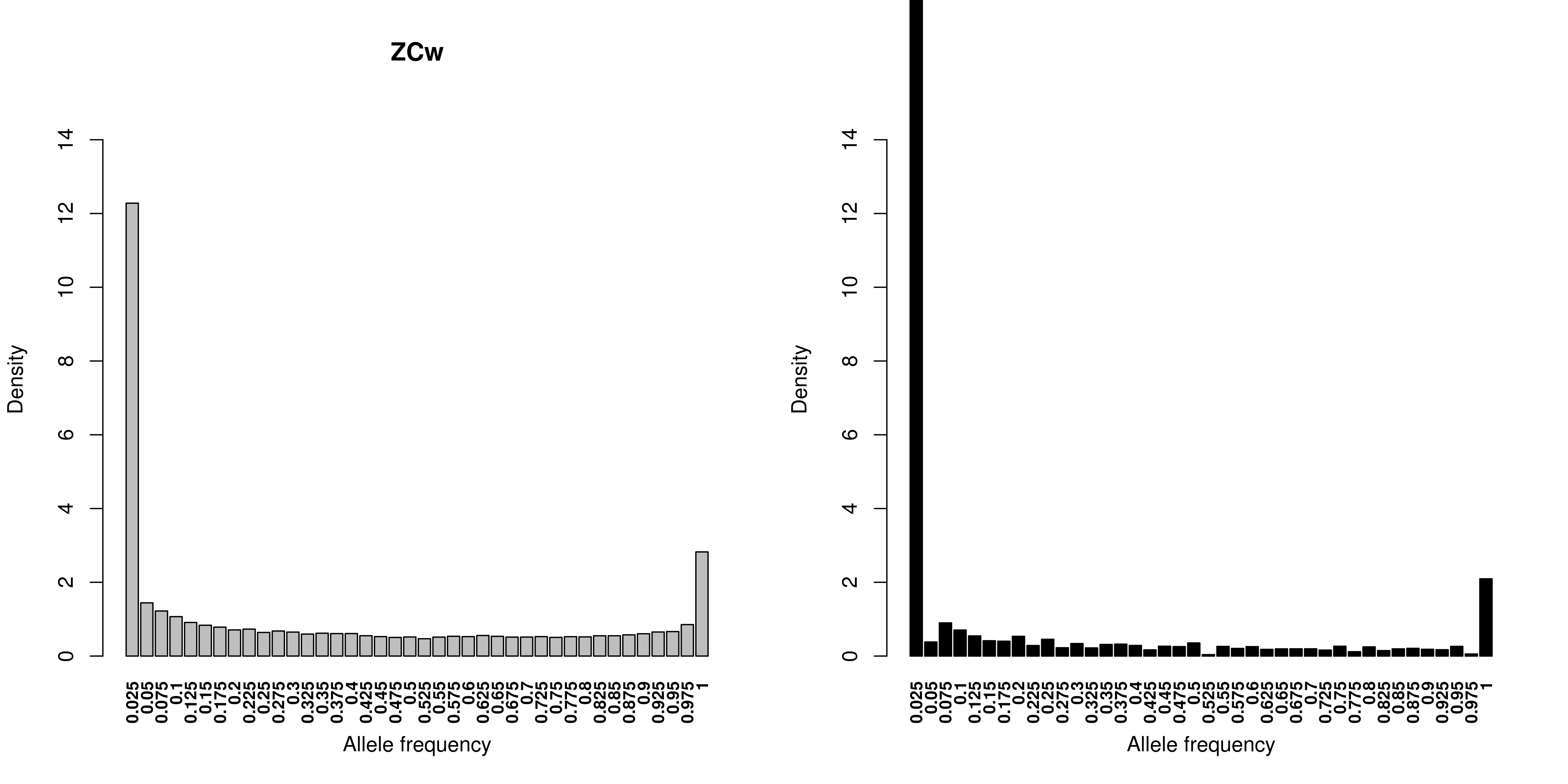

Supplement: Supplementary file 1 — Zip file containing allele frequency spectrum figures of each population. (ZIP 11230 kb) [file 12864_2017_4416_MOESM1_ESM.zip › additional_1 - Copy/AFS_array_WGS_ZCw.tif]
