# Supplementary material for: Efficiency of different strategies to mitigate ascertainment bias when using SNP panels in diversity studies
Source: BMC Genomics. 2018 Jan 5;19:22. doi: 10.1186/s12864-017-4416-9 (PMC5756397; doi:10.1186/s12864-017-4416-9)
Supplement: Supplementary file 2 — Proportion of SNPs in genic and non-genic in WGS and array data. Table S2. Population clusters. Table S3. Topological distances between NJ trees of WGS and array data based on Billera method. Table S4. Topological distances between NJ trees of WGS and array data based on Penny and Hendy method. Table S5. Topological distances among the array versions. (DOCX 25 kb) [file 12864_2017_4416_MOESM2_ESM.docx]

**Table S1.** Proportion of SNPs in genic and non-genic in WGS and array data.

|  | **Array (n=401,125)** | | **Pools (n=29,737,210)** | |
| --- | --- | --- | --- | --- |
|  | **Number** | **Percentage** | **Number** | **Percentage** |
| **Genic** | 158,882 | 39.61 | 11,873,869 | 39.93 |
| **Non-Genic** | 242,243 | 60.39 | 17,863,341 | 60.07 |
| **Exonic** | 17,831 | 0.044 | 364,900 | 0.012 |

**Table S2.** Population clusters.

| **Breed and abbreviation** | **Cluster** | **Name in sequence pools** |
| --- | --- | --- |
| **Commercial breeds :** |  |  |
| WL_A – White Leghorn line A | **4** | pl_WL_0043 |
| BL_A – Rhode Island Red line A | **1** | pl_RI_0043 |
| BL_D – White Rock line D | **1** | pl_WR_0043 |
| **Wild populations :** |  |  |
| GGg – Gallus Gallus Gallus | **2** | pl_GG_0043 |
| GGsc – Gallus Gallus Spadiceus | **2** | pl_GS_0043 |
| **European populations:** |  |  |
| ABwa – Barbue d’Anvers quail | **3** | pl_AB_0043 |
| ARsch – Rumpless Araucana black | **n1** | pl_AR_0043 |
| BAsch – Rosecomb Bantam black | **3** | pl_BA_0043 |
| BKschg – Bergische Crower | **4** | pl_BK_0043 |
| DZgh – German Bantam gold partridge | **3** | pl_DG_0043 |
| FZgpo – Booted Bantam millefleur | **3** | pl_FG_0043 |
| HOxx – Dutch White Crested | **4** | pl_HO_0043 |
| ITrh – Leghorn brown | **4** | pl_IT_0043 |
| KAsch – Castilians black | **4** | pl_KA_0043 |
| KRsch – Creeper black | **4** | pl_KS_0043 |
| KRw – Creeper white | **4** | pl_KW_0043 |
| LER11- ­White Leghorn line R11 | **4** | pl_LE_0043 |
| OMsschg - East Friesian Gulls silver penciled | **4** | pl_OM_0043 |
| PAxx - Poland any colour | **4** | pl_PA_0043 |
| SBsschs - Sebright Bantam silver | **3** | pl_SB_0043 |
| WTs - Westphalian Chicken silver | **4** | pl_WT_0043 |
| **Asian populations:** |  |  |
| ASrb – Aseel red mottled | **2** | pl_AS_0043 |
| BHrg – Brahma gold | **1** | pl_BH_0043 |
| CHgesch – Japanese Bantam black tailed buff | **2** | pl_CG_0043 |
| CHschw – Japanese Bantam black mottled | **2** | pl_CW_0043 |
| COsch – Cochin black | **1** | pl_CS_0043 |
| DLIa – German Faverolles salmon | **1** | pl_DL_0043 |
| KSgw – Ko Shamo black-red | **2** | pl_KG_0043 |
| MAxx – Malay black red | **2** | pl_MA_0043 |
| MRschk – Marans copper black | **1** | pl_MR_0043 |
| NHL68 – New Hampshire line 68 | **1** | pl_NH_0043 |
| OFrbx – Orloff red spangled | **n2** | pl_OF_0043 |
| OHsh - Ohiki silver duckwing | **2** | pl_OH_0043 |
| ORge - Orpington buff | **1** | pl_OR_0043 |
| SAsch - Sumatra black | **n3** | pl_SA_0043 |
| SEw - Silkies white | **1** | pl_SE_0043 |
| SHsch - Shamo black | **2** | pl_SH_0043 |
| SNwsch - Sundheimer light | **1** | pl_SN_0043 |
| TOgh - Toutenkou black breasted red | **2** | pl_TO_0043 |
| WYw - Wyandotte white | **1** | pl_WY_0043 |
| YOwr - Yokohama red saddled white | **2** | pl_YO_0043 |
| ZCw - Pekin Bantam white | **1** | pl_ZC_0043 |

**Table S3.** Topological distances between NJ trees of WGS and array data based on Billera method.

|  |  | **Array mean ± SE** |
| --- | --- | --- |
|  | **WGS range** |  |
| **Array_all** | 0-0.005 | 0.043 ± 0.00002 |
| **Array_MAF5** | 0-0.005 | 0.047 ± 0.00002 |
| **GG** | 0-0.006 | 0.050 ± 0.00002 |
| **GG_MAF5** | 0-0.006 | 0.052 ± 0.00002 |
| **Pruned** | 0-0.006 | 0.027 ± 0.00004 |
| **Pruned_MAF5** | 0-0.006 | 0.034 ± 0.00004 |
| **Pruned_GG** | 0-0.007 | 0.037 ± 0.00004 |
| **Pruned_GG_MAF5** | 0-0.007 | 0.040 ± 0.00004 |

WGS ranges are estimated topological distances between 100 sampled replicates, while the array mean is a mean of the topological distances between the array data set and the 100 WGS replicates

**Table S4.** Topological distances between NJ trees of WGS and array data based on Penny and Hendy method.

|  |  | **Array mean ± SE** |
| --- | --- | --- |
|  | **WGS range** |  |
| **Array_all** | 0-26 | 8.34 ± 0.408 |
| **Array_MAF5** | 0-26 | 10.0 ± 0.414 |
| **GG** | 0-30 | 8.88 ± 0.515 |
| **GG_MAF5** | 0-30 | 8.88 ± 0.515 |
| **Pruned** | 0-32 | 18.06 ± 0.479 |
| **Pruned_MAF5** | 0-32 | 18.06 ± 0.575 |
| **Pruned_GG** | 0-36 | 19.14 ± 0.602 |
| **Pruned_GG_MAF5** | 0-36 | 19.14 ± 0.602 |

WGS ranges are estimated topological distances between 100 sampled replicates, while the array mean is a mean of the topological distances between the array data set and the 100 WGS replicates.

**Table S5.** Topological distances among the array versions.

|  | **Array_all** | **Array_MAF5** | **GG** | **GG_MAF5** | **Pruned** | **Pruned_MAF5** | **Pruned_GG** | **Pruned_GG_MAF5** |
| --- | --- | --- | --- | --- | --- | --- | --- | --- |
| **Array_all** |  | 4 | 6 | 6 | 16 | 16 | 16 | 16 |
| **Array_MAF5** | 0.011 |  | 2 | 2 | 18 | 18 | 18 | 18 |
| **GG** | 0.015 | 0.009 |  | 0 | 16 | 16 | 16 | 16 |
| **GG_MAF5** | 0.016 | 0.010 | 0.002 |  | 16 | 16 | 16 | 16 |
| **Pruned** | 0.024 | 0.029 | 0.030 | 0.032 |  | 0 | 0 | 0 |
| **Pruned_MAF5** | 0.021 | 0.025 | 0.026 | 0.027 | 0.007 |  | 0 | 0 |
| **Pruned_GG** | 0.021 | 0.025 | 0.025 | 0.026 | 0.011 | 0.004 |  | 0 |
| **Pruned_GG_MAF5** | 0.021 | 0.024 | 0.023 | 0.024 | 0.014 | 0.007 | 0.003 |  |

Above the diagonal are the topological distances based on Penny and Hendy method and below the diagonal are the distances based on Billera method.
