# Supplementary material for: Efficiency of different strategies to mitigate ascertainment bias when using SNP panels in diversity studies
Source: BMC Genomics. 2018 Jan 5;19:22. doi: 10.1186/s12864-017-4416-9 (PMC5756397; doi:10.1186/s12864-017-4416-9)
Supplement: Supplementary file 3 — Comparion of MAF between genic and non-genic regions in array (left) and WGS (right) data. Figure S2. Comparisons of expected heterozygosity (He) estimates between WGS (boxplot of 100 replicates) and array (Array_all, GG and Pruned) data, for all 42 populations. Figure S3. Expected heterozygosity (He) estimated with array vs. WGS data for the 39 populations. Figure S4. Three dimensional PCA plot of A) WGS, B) GG and C) Pruned array data. (PDF 761 kb) [file 12864_2017_4416_MOESM3_ESM.pdf]

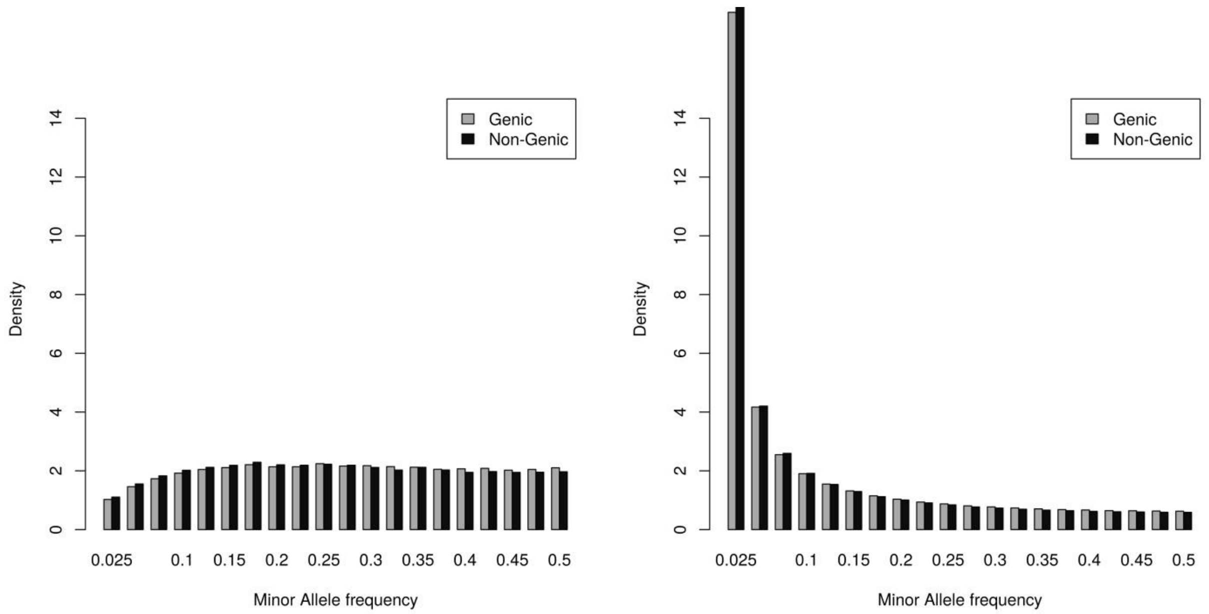

Figure S1. Comparion of MAF between genic and non-genic regions in array (left) and WGS (right) data.

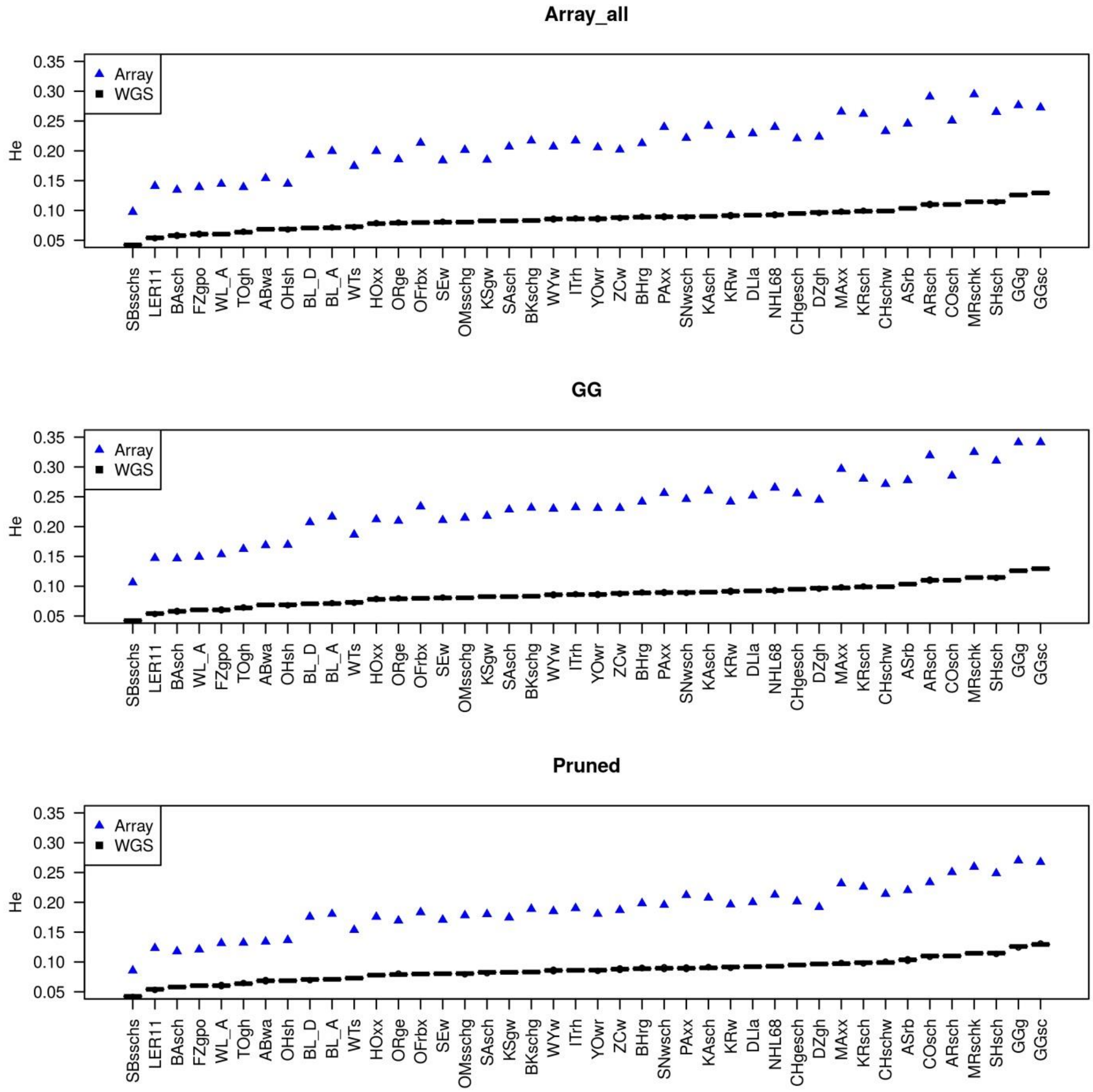

Figure S2. Comparisons of expected heterozygosity (H_e) estimates between WGS (boxplot of 100 replicates) and array (Array_all, GG and Pruned) data, for all 42 populations.

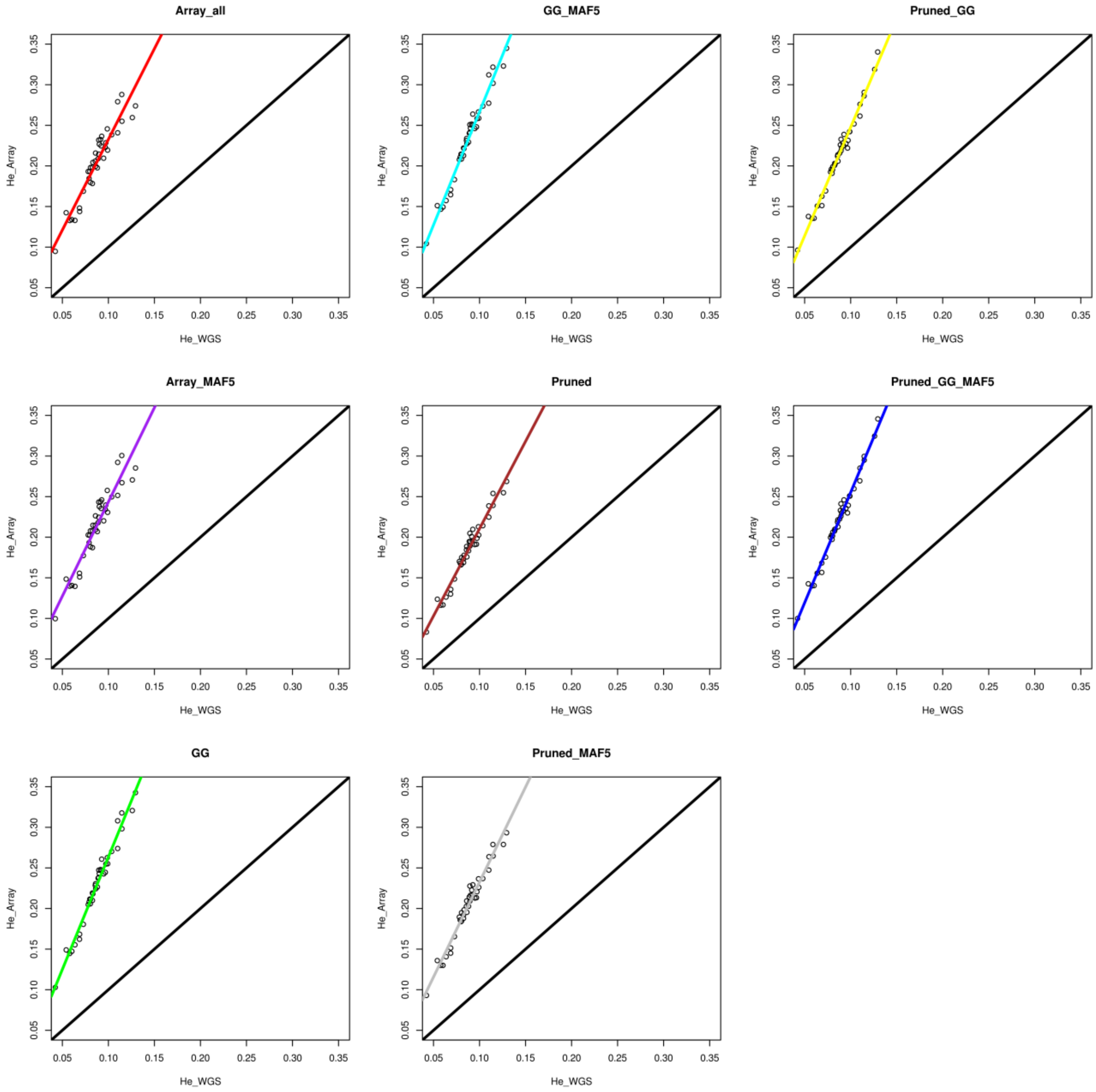

Figure S3. Expected heterozygosity (H_e) estimated with array vs. WGS data for the 39 populations.

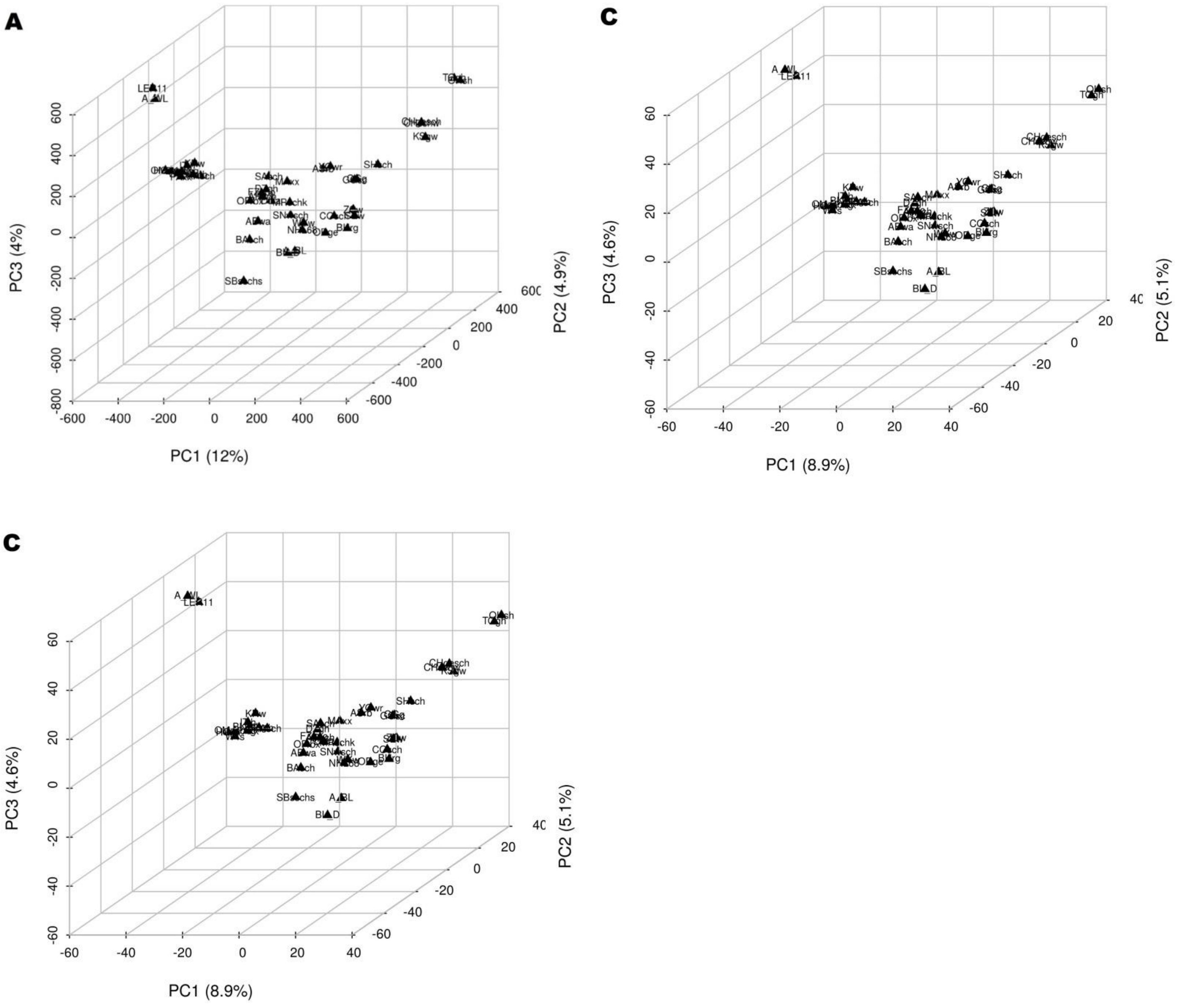

Figure S4. Three dimensional PCA plot of A) WGS, B) GG and C) Pruned array data.
